# Developing efficient mechanochemical routes to porous metal–organic polyhedra, supported by 3D electron diffraction

**DOI:** 10.1039/d6sc02926d

**Published:** 2026-08-03

**Authors:** Megan G. Wilkinson, Calum S. Sangster, Jeremiah P. Tidey, Martin R. Ward, Simon Parsons, Ashleigh J. Fletcher, Gavin A. Craig

**Affiliations:** a Department of Pure and Applied Chemistry, University of Strathclyde Glasgow G1 1RX UK gavin.craig@strath.ac.uk; b EaStCHEM, School of Chemistry and Centre for Science at Extreme Conditions, University of Edinburgh Edinburgh EH9 3FJ UK; c Department of Physics, University of Warwick Gibbet Hill Road Coventry CV4 7AL UK; d Centre for Continuous Manufacturing and Advanced Crystallisation (CMAC), University of Strathclyde Glasgow G1 1XW UK; e Department of Chemical and Process Engineering, University of Strathclyde Glasgow G1 1XJ UK

## Abstract

Metal–organic polyhedra (MOPs) are the porous molecular counterparts to metal–organic frameworks (MOFs). While mechanochemistry has been successfully applied to a wide range of MOFs, this synthetic approach has found more limited use for MOPs. Herein, two mechanochemical routes have been developed for a series of MOPs presenting a lantern geometry. The cages can be formed through: Route 1, grinding with DMA, and therefore achieving a reduction in solvent usage of 90%; or through Route 2, grinding with MeOH to completely eliminate the use of non-volatile, highly toxic solvents. The development of Route 2 is facilitated by 3D electron diffraction, which proved the formation of discrete cages that would otherwise be inaccessible through direct solvothermal synthesis. In all cases, the degree of crystallinity of the mechanochemical products is equal or superior to material obtained through solvothermal methods, and exhibits comparable porosity. As well as presenting a method to improve the sustainability of the synthesis of these cages, this work extends the range of porous cages to which mechanochemistry can be applied. Given the known dependence of the porosity of MOPs on the crystal packing of their parent solvated forms, Route 2 suggests that future work could probe the formation of new solvated phases that display distinct gas sorption behaviour.

## Abstract 2

Mechanochemistry enables the elimination of highly toxic solvents in the synthesis of porous metal–organic polyhedra.

## Graphical abstract

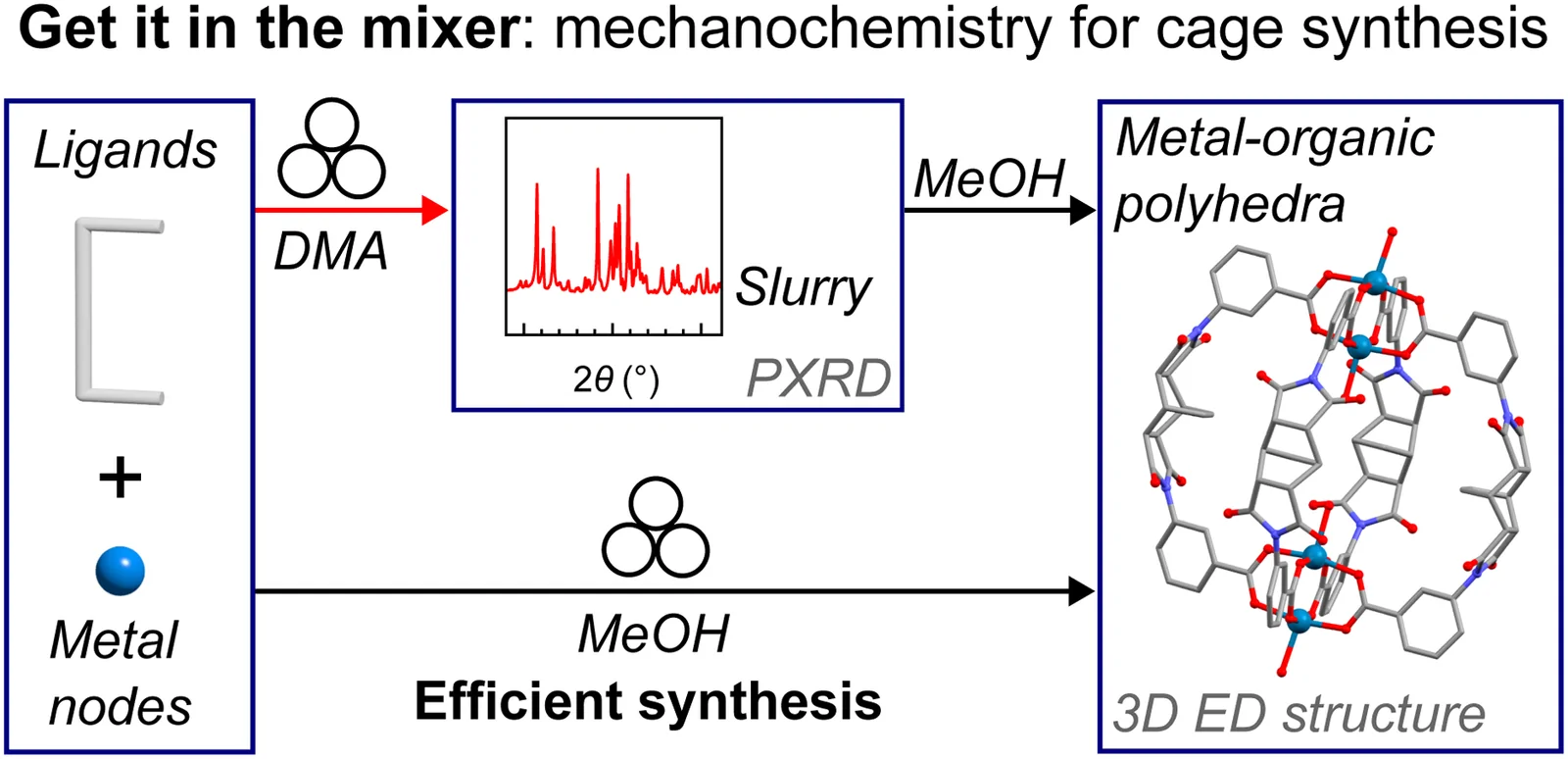

## Introduction

Mechanochemical synthesis offers a reduction in the use of solvent and, in turn, costs and waste, that has spurred its widespread use in organic chemistry^1–4^ and crystal engineering.^5–8^ It is also effective in the synthesis of metal–organic frameworks (MOFs), coordination polymers that are proposed for a wide range of applications arising from their potential porosity. It was initially found that a range of 1D coordination polymers could be prepared using mechanochemistry, without proving their porosity,^9–12^ while the first 3D microporous MOF to be synthesised through mechanochemistry was reported by Pichon *et al.* in 2006.^13^ The authors made Cu(INA)_2_ (where INA = isonicotinate) by ball milling the reactants neat at 25 Hz for 10 minutes. Since then, there have been many MOFs made using mechanochemistry, including archetypal frameworks such as HKUST-1, MOF-5, UiO-67 and ZIF-8.^14–20^

Porous metal–organic polyhedra (MOPs) are the molecular counterparts to MOFs, and share many of the same synthetic features and potential applications.^21–23^ However, there are limited reports of mechanochemical synthesis of MOPs. Although powder X-ray diffraction (PXRD) is an obvious technique to probe the mechanochemically-driven formation of complexes, work on Pd(ii) and Pt(ii)-based square metallocycles and octahedral cages uses solution-phase NMR spectroscopy: first the reactants are milled, before the resulting solids are dissolved and the NMR spectrum run.^24–27^ This raises the possibility that complex formation occurs in the solution phase, where the reduced particle size brought about by milling enables faster reaction. The limitations of PXRD for identifying cages were highlighted by Bloch and co-workers in work on cuboctahedral MOPs.^28,29^ They initially found that, by grinding copper acetate and isophthalic acid in a ball mill in a 1 : 1.1 ratio, it was possible to obtain MOP-1 ([Cu_24_(bdc)_24_], where bdc = 1,3-benzene dicarboxylate),^29^ before extending this method to functionalised cuboctahedral MOPs.^28^ Nonetheless, if the obtained diffractogram does not match the phases of known materials then obtaining unambiguous proof of the cage formation can be impossible; a problem that is exacerbated by the ability of cages to present a range of solvent dependent packings in the solid state.^30,31^ A potential solution to these problems is the use of 3D electron diffraction (3D ED) to obtain the structures of the ball-milled materials, where crystallites are usually too small for conventional single-crystal approaches.^32^ 3D ED has been applied to elucidate the structures of MOFs where only small crystallites are available,^33–35^ and has successfully identified the products of mechanochemically-synthesised coordination polymers.^36–39^

Here, we demonstrate the applicability of mechanochemistry and 3D ED to develop more efficient routes in the synthesis of porous MOPs. In the first instance, we show that the volume of dimethylacetamide (DMA) that is needed for the synthesis of porous cages can be reduced 10-fold, when comparing the mechanochemical synthetic approach to solution-based synthesis (Fig. 1). Reducing the use of reprotoxic solvents such as DMA is an important step in the future of green chemistry, particularly relevant after a recent update to the European Union's regulations, where the use of DMA will now be heavily restricted.^40^ These materials can be solvent-exchanged with methanol (MeOH), prior to activation and measurement of their porosity. We then demonstrate a more efficient synthesis that eliminates the use of DMA entirely, by grinding the reactants in the presence of MeOH, using 3D ED to prove the formation of discrete cages that cannot be obtained using direct solvothermal synthesis in MeOH. These materials are at least, if not more, crystalline than the cages obtained *via* other routes. The porosity of all of these materials is probed with measurement of N_2_ and CO_2_ sorption, validating this mechanochemical approach to obtain porous cages.

**Fig. 1:**
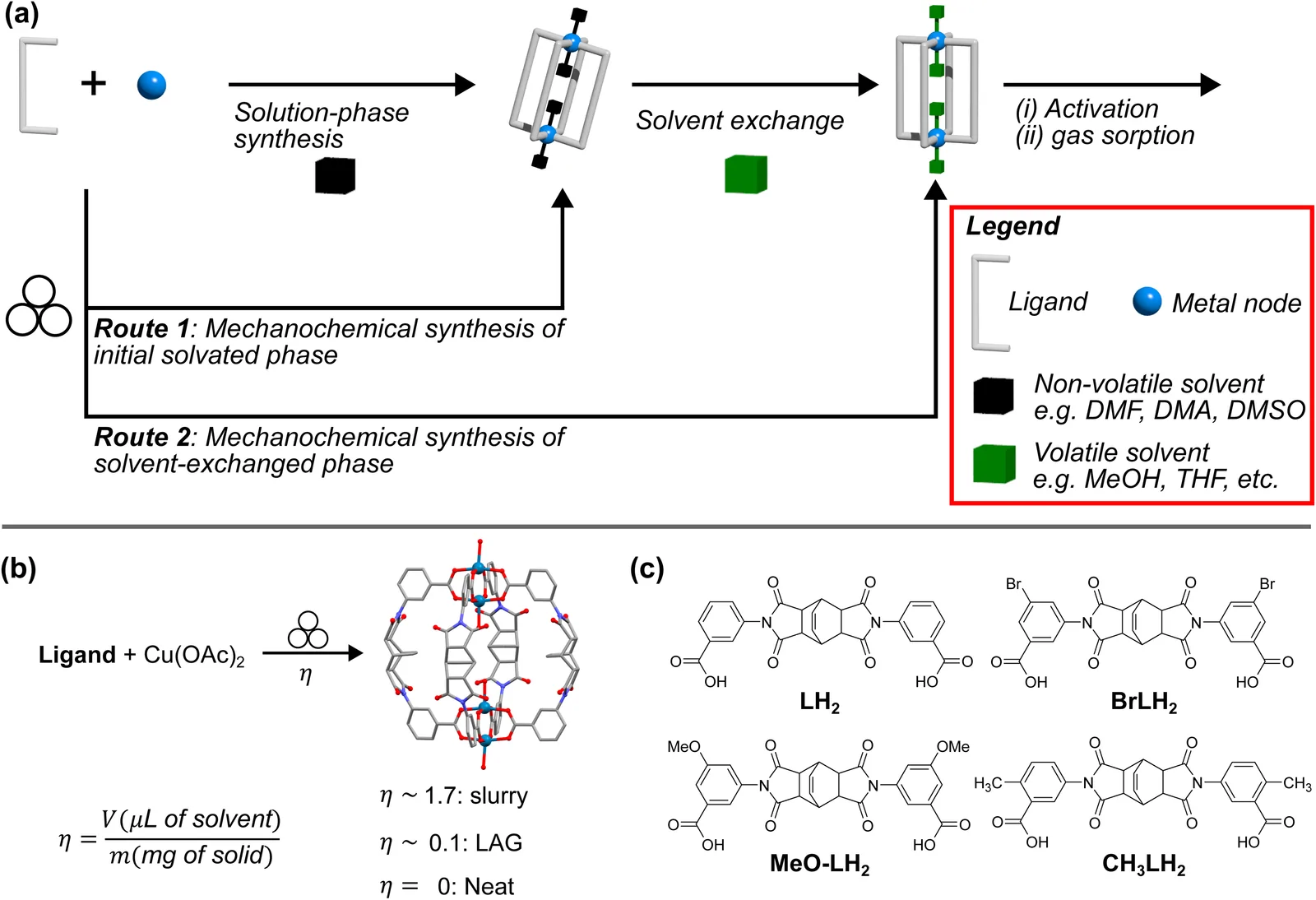
(a) Mechanochemical routes to porous MOPs. Conventional methods involve solution-based syntheses in solvents such as DMA or DMF, confirming structure with single crystal X-ray diffraction, prior to solvent exchange. Here, we describe Route 1, which involves grinding in the presence of a non-volatile solvent, while Route 2 targets direct formation of the solvent-exchanged phase that is used for activation and gas sorption measurements. (b) Synthetic parameters for the mechanochemical synthesis in this paper, with definition of the values of *η* taken from ref. 43. (c) Ligands used for the mechanochemical synthesis of the MOPs.

## Results and discussion

We targeted the mechanochemical formation of four lantern-type MOPs formed from ligands containing bicyclo[2.2.2]oct-7-ene units – LH_2_, CH_3_LH_2_, MeO-LH_2_, and BrLH_2_ (Fig. 1). The reported solvothermal route to these cages involves heating DMA solutions of copper(ii) acetate with the corresponding ligand at 80 °C, leading to the formation of crystals that are suitable for single crystal X-ray diffraction.^41,42^ Our initial motivation was to use mechanochemistry to significantly reduce the volume of DMA required for the formation of the cages, following Route 1 outlined in Fig. 1, where the reported crystallographic data are a convenient handle to prove material formation. Mechanochemical synthetic procedures can be characterised by the parameter *η*, which is defined as the ratio of the volume of liquid, in microlitres, to the total mass of solids used, in milligrams. Broadly, previous research has established four regimes for *η*: *η* ≈ 10 corresponds to the solution phase; *η* ≈ 1 is termed a slurry; *η* ≈ 0.1 is considered as liquid-assisted grinding (LAG); and the complete absence of liquid is neat grinding, with *η* = 0.^43^ We probed slurry-based, LAG, and neat approaches to the mechanochemical synthesis of the target MOPs, grinding the relevant ligand and copper(ii) acetate in a 1 : 1 ratio in the presence of the requisite volume of DMA at 30 Hz for 60 minutes using a mixer mill (see SI for full experimental details). The labelling for the products follows the system X-MOP^R1^_η_, where X denotes the ligand used; the super-script R1 refers to Route 1, grinding with DMA; and *η* gives the mechanochemical regime of the synthesis. The obtained solids were characterised using infra-red (IR) spectroscopy and PXRD in the first instance to determine the success of a given synthesis through comparison with samples made using solvothermal synthesis.^41,42^

The IR spectra and PXRD data for the samples using the ligand CH_3_LH_2_, where the targeted cage is [Cu_4_(CH_3_L)_4_(DMA)_2_(H_2_O)_2_]⋅*solv.*, are shown in Fig. 2. In the IR spectra, the key regions for determining the formation of the MOP are between 1800 and 1550 cm^−1^, and between 850 and 600 cm^−1^. These regions correspond to the C=O stretches in the carboxylate groups that coordinate to the copper sites, and to deformations from the substitution on the benzene ring in each of the ligands, respectively. When CH_3_-MOP is synthesised solvothermally, the formation of the MOP is associated with a clearly resolved pair of peaks at around 690 cm^−1^, attributed to C–H deformations from the unsubstituted positions of the benzene ring, as is observed for CH_3_-MOP^R1^_slurry_. In contrast, the samples yielded by LAG and neat grinding show a broader set of overlapping peaks in this region of the spectra. The PXRD data for CH_3_-MOP samples show CH_3_-MOP^R1^_slurry_ to give a good match with the material obtained through solvothermal synthesis (Fig. 2(b)). Both the neat and the LAG samples, however, are dominated by diffraction from unreacted copper(ii) acetate (Fig. S2). On the basis of the IR spectra and PXRD data, a similar trend was identified for the other three cages – slurry-based mechanochemistry is effective in forming the MOP, while LAG and neat grinding yielded largely unreacted mixtures of the ligand and copper(ii) acetate (the data for the other three cages, MOP, MeO-MOP, and Br-MOP are provided in SI Fig. S3–S5). NMR digestion experiments were used to quantify the amount of DMA that was present in the samples yielded by the slurries (Fig. S6–S9). This was found to be 13 DMA per MOP for MOP^R1^_slurry_; 11 for CH_3_-MOP^R1^_slurry_; 19 for MeO-MOP^R1^_slurry_ and 19 for Br-MOP^R1^_slurry_. The NMR spectra for all four cages contain a peak at *δ* = 1.9 ppm, consistent with the formation of acetic acid as a by-product from the syntheses. The slurry-based MOPs were analysed by scanning electron microscopy (SEM) to assess the morphology and particle size, showing that the mechanochemically synthesised cages present uniform distributions of small particles, consistent with other mechanochemical studies (Fig. S3–S5 and S10).^19^ The success of the mechanochemical approach here expands the reported MOP geometries that have been obtained using mechanochemistry, and represents a reduction in the use of DMA by 90%.

**Fig. 2:**
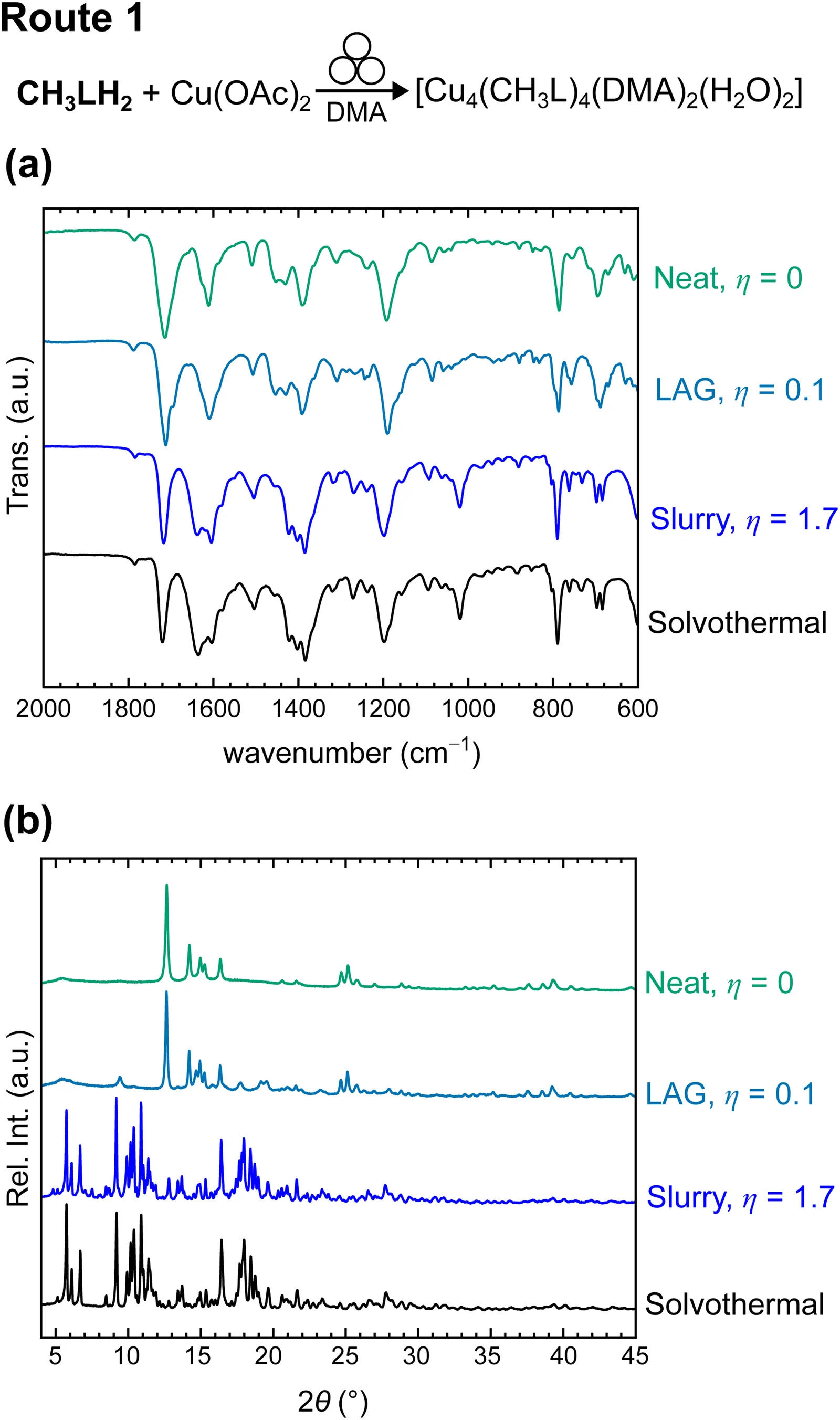
Data for the Route 1 mechanochemical approach using CH_3_LH_2_ as ligand in MOP synthesis (a) IR spectra obtained from the value of *η* used. (b) PXRD data for the products of the reactions performed under the conditions indicated.

A general step in the preparation of MOPs for gas sorption experiments is solvent exchange of the solvent used in the initial synthesis (here, DMA) for one that is more volatile, in this case MeOH (Fig. 1). This process changes the crystal packing of the cages, as observed with PXRD measurements (Fig. S11–S14). For each particular cage, the IR spectra obtained after solvent exchange for these new phases are the same, regardless of the mechanochemical conditions used to synthesise the initial sample: that is, the neat grinding and LAG samples that had not reacted upon initial grinding then converge on the same phase as the slurry-based sample when soaked in MeOH. As for the products of the initial ball-milling step, the results of this process for MOP^R1^_slurry_, CH_3_-MOP^R1^_slurry_, MeO-MOP^R1^_slurry_ and Br-MOP^R1^_slurry_, were compared with the results of the same process performed on solvothermally-obtained materials. For Br-MOP^R1^_slurry_,_MeOH_, the crystallinity is comparable with the solvothermal counterpart (Fig. S14). In contrast, the PXRD data for MOP^R1^_slurry,MeOH_; CH_3_-MOP^R1^_slurry_,_MeOH_ and MeO-MOP^R1^_slurry_,_MeOH_ show that the samples obtained through mechanochemical synthesis followed by solvent exchange present a higher degree of crystallinity than those obtained *via* solvothermal routes. Solvent exchange removes the acetic acid that remains after initial mechanochemical grinding (NMR spectra for the materials are presented in Fig. S6–S9), and these four samples were carried forward for gas sorption measurements (see below).

We then probed if the solvent-exchanged phases could be synthesised directly using ball milling, by grinding the reactants with MeOH (Fig. 1: Route 2), thereby entirely eliminating the use of DMA from the synthetic procedure. This approach produced the samples MOP^R2^, CH_3_-MOP^R2^, MeO-MOP^R2^ and Br-MOP^R2^, where the super-script R2 denotes Route 2. The IR spectra for all four cages (Fig. S11–S14) align with the spectra of cages obtained *via* the sequential solvothermal and solvent exchange method. The ^1^H-NMR digestion data for all of the cages present a peak related to the acetic acid, which is washed out with MeOH (Fig. S6–S9). The PXRD data also show a good match and high crystallinity (Pawley refinements provided in Fig. S15–S18), with noticeably improved crystallinity in the case of MOP^R2^ and CH_3_-MOP^R2^ compared to samples obtained from the sequential solvothermal and solvent exchange method (Fig. 3).

**Fig. 3:**
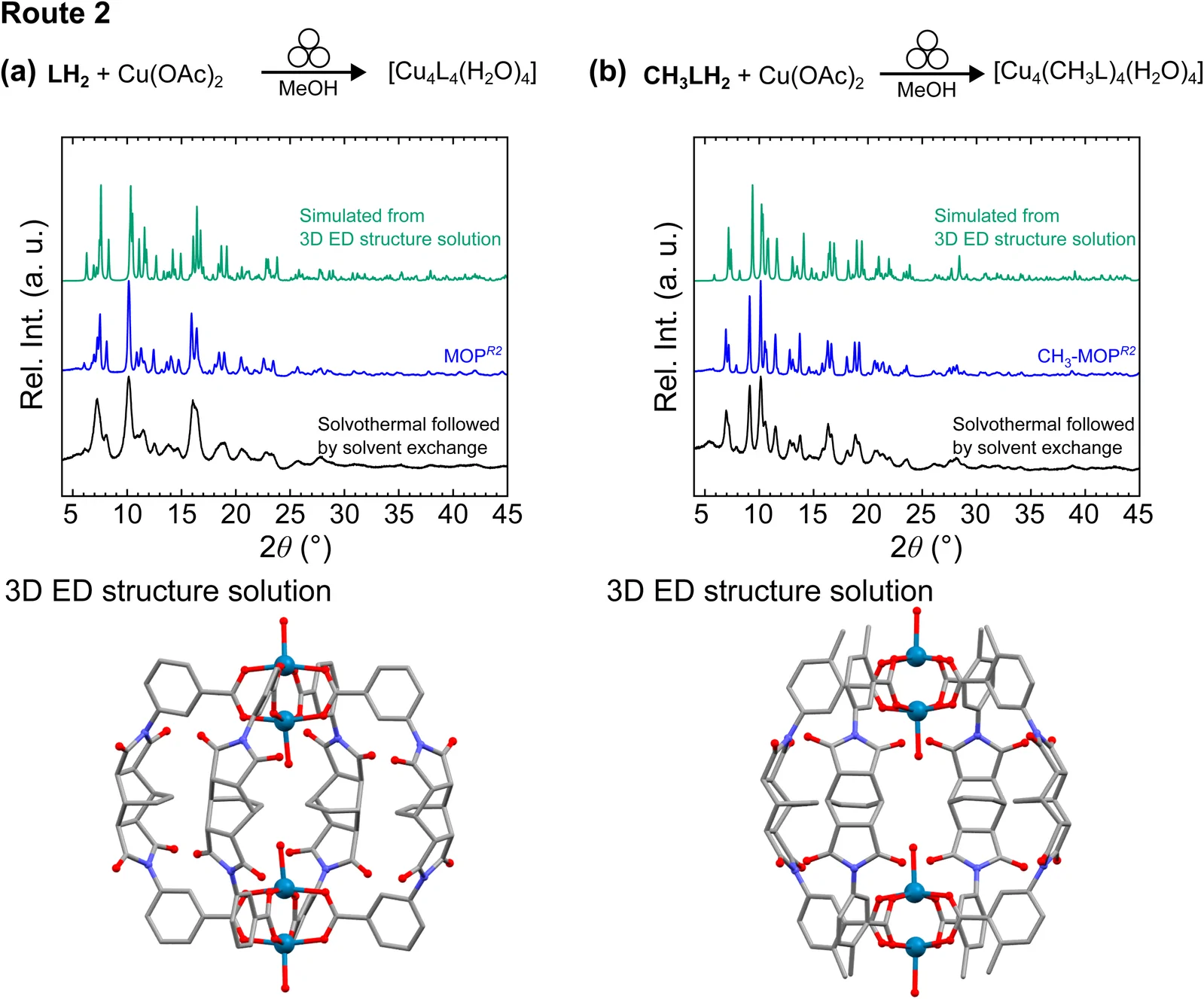
(a, top) Route 2 synthesis, where the ligand and salt are ground in MeOH. (Middle) PXRD data comparing the simulated pattern obtained from the 3D ED structure solution with the experimentally obtained data from Route 2 (MOP^R2^) and the data obtained when the sample is synthesised solvothermally and then solvent exchanged in MeOH. (Bottom) The structure of the cage [Cu_4_L_4_(H_2_O)_4_] obtained using 3D ED. (b) Equivalent data for the cage CH_3_-MOP^R2^.

Inspection of the SEM images obtained for all of these products (Fig. S19), together with the high crystallinity observed with PXRD, prompted us to use 3D ED to determine the structures of the materials yielded by Route 2. 3D ED has been applied extensively to porous framework materials,^44–49^ but its use for MOP-based materials has emerged only recently.^50,51^ Solution and refinement of the data obtained for MOP^R2^ and CH_3_-MOP^R2^ gave structural models that were formulated as [Cu_4_L_4_(H_2_O)_4_]·8(H_2_O)·1(MeOH) for MOP^R2^, and [Cu_4_(CH_3_-L)_4_(H_2_O)_4_]·6.3(H_2_O)·3.7(MeOH) for CH_3_-MOP^R2^ (Fig. 3; details for data collection are provided in SI Table S2). Both cages crystallise in the triclinic space group *P*1̄, where the molecule sits on an inversion centre, such that the asymmetric unit consists of one paddlewheel coordinated by two ligands. The two axial sites of the paddlewheels in both cages are occupied by water molecules. The precise stoichiometry of the non-coordinated solvents is not definitive due to challenges associated with the materials and the nature of 3D ED data collection, but the structures prove that Route 2 leads to the formation of discrete lantern-type cages, rather than polymeric materials. The samples of MeO-MOP^R2^ and Br-MOP^R2^, which showed lower crystallinity in PXRD measurements, did not diffract beyond a resolution of ∼2.0 Å, but were sufficient to obtain unit cell information for the materials. The volume and dimension of the unit cells obtained from these 3D ED experiments and Pawley fits are qualitatively similar to the MOPs MOP^R2^ and CH_3_-MOP^R2^, which would suggest the likely formation of discrete MOPs. Preliminary attempts to solve and refine the structure for both of these materials are included in SI for completeness. These 3D ED data confirm the successful formation of the MOPs through Route 2, and therefore confirm that, by switching to a mechanochemical approach, DMA can be eliminated entirely from the synthesis of these porous cages.

The precise role of liquid additives in mechanochemical reactions is subject to intensive study.^52–54^ Where the solvent additive can solubilise the reagents, it is apparent that this dissolution will be a major driving force in the reaction. This is the case for Route 1, where both ligand and metal salt are soluble to some extent in the DMA additive. While all of the ligands here are fully soluble in DMA when working at the concentrations used for solvothermal synthesis, which correspond to a value of *η* > 20, they show varied solubility when working at the slurry-type concentrations associated with mechanochemistry. MeO-LH_2_ and BrLH_2_ are found to be completely soluble at this concentration by NMR spectroscopy (see Table S3 and Fig. S20), forming a viscous liquid. The ligand LH_2_ is also soluble, but extremely close to saturation – tests on large scales of the order that was used in the mechanochemistry would crash out or not fully solubilise, while small scale tests (*ca*. 40 mg) would dissolve. CH_3_LH_2_ at this concentration of DMA would soak up the solvent, which meant it was challenging to quantify its solubility (see Fig. S21). The metal salt was found to be partially soluble in these conditions – of a maximum possible concentration of 0.80 M, we determined an experimental concentration of 0.16 M in slurry-type conditions (Fig. S24). Therefore, in the case of Route 1 syntheses, we suggest the reaction is taking place in the limited solvent available, with mechanochemistry enhancing the mixing. To test this, we performed two sets of experiments on these systems. We directly added the solid copper(ii) acetate to the slurry concentrations of ligand in DMA, with no mixing. For the reactions involving MeO-LH_2_, BrLH_2_, and CH_3_LH_2_, the solids recovered after 24 hours presented weak peaks in the PXRD data corresponding to the products, which were dominated by other phases (Fig. S25–S27). Using the system based on LH_2_, we performed the same experiment, but varied the value of *η* by reducing the volume of DMA used (Fig. S28). On reduction to a slurry value of *η* = 1.6, the PXRD data are dominated by copper acetate. This supports the suggestion that there is a minimum quantity of solvent required for Route 1 to be successful, with mechanochemistry required to accelerate the reaction and drive it to completion.

For Route 2, where the solvent additive is MeOH, all four ligands are insoluble in this solvent even when diluting to the concentration associated with solvothermal conditions (Table S3). The metal salt was found to be partially soluble in MeOH in the slurry-type conditions. We propose then that the partially dissolved salt is reacting on the surface of the insoluble ligand particles, with mechanochemistry reducing the particle size to increase the reactant surface area and accelerate the reaction.^43,55,56^ This would align with our observation that neatly ground samples can be transformed into the MOP upon prolonged soaking in MeOH.

To assess the effect of the different synthetic routes on the porous properties of the cages, the materials obtained from Route 1 and Route 2 were studied using N_2_ and CO_2_ sorption measurements at −196 °C and 0 °C, respectively, after activation under vacuum at 120 °C. The samples obtained mechanochemically for Br-MOP and MeO-MOP show low uptake of N_2_, regardless of the mechanochemical route, which is consistent with the solvothermally obtained samples (Fig. 4(a); detailed analysis shown in Fig. S29–S32). In contrast, the MOP and CH_3_-MOP samples show modest gas uptakes. The adsorption isotherms for MOP^R2^ and MOP^R1^_slurry_ reach a plateau at *P*/*P*_0_ ≈ 0.05, where the uptakes are 68 and 82 cm^3^ g^−1^, respectively. At higher pressures (*P*/*P*_0_ > 0.9), there is a further increase in the uptake, due to total pore filling from the bulk condensation of N_2_ (Fig. 4(a), S33 and S34). The Brunauer, Emmett and Teller (BET) surface areas calculated according to the Rouquerol criteria yielded values of 283 m^2^ g^−1^ (MOP^R1^_slurry_) and 346 m^2^ g^−1^ (MOP^R2^), surpassing that found for the material obtained from sequential solvothermal synthesis and solvent exchange (MOP, 120 m^2^ g^−1^; Fig. S35). However, this improvement in the surface area through mechanochemical synthesis is not general for these lantern cages: for the CH_3_-MOP samples, both mechanochemical routes lead to materials with appreciable surface areas (CH_3_-MOP^R2^: 409 m^2^ g^−1^; CH_3_-MOP^R1^_slurry_: 444 m^2^ g^−1^; Fig. S36 and S37), but these are lower than found for the solvothermally obtained material (521 m^2^ g^−1^).^41^ The gas sorption data were further analysed using the Dubinin–Radushkevich equation (see Table S4). This analysis shows that the mechanochemical MOP samples display a higher micropore volume than the solvothermal sample; while for the CH_3_-MOP samples, the micropore volume is lower for the ball-milled samples than the solvothermal sample. The forms of the isotherms for all of the compounds are the same regardless of the mechanochemical route, suggesting that the pore shape in the materials is likely the same, and that differences in capacity may arise from slight differences in the extrinsic porosity. This extrinsic porosity will be shaped by packing of the individual molecules, but also defects within particles, as well as pore accessibility. We therefore tentatively attribute the increase in surface area for the MOP samples, where the cage is unfunctionalised, to the higher degree of crystallinity that is observed in the ball milled samples than is observed in the solvothermally-obtained sample (Fig. 3). For the CH_3_-MOP samples, where there is external functionalisation of the cages, it may be the case the porosity is less dependent on the precise degree of crystallinity and that there is a greater contribution from defects and extrinsic porosity generated by surface functionalisation, with all of the samples being close to the limit of porosity for these cages. We also note that differences in surface areas and capacity between solvothermal and mechanochemical samples are also found for MOFs.^57^ All of the mechanochemically synthesised samples show uptake of CO_2_ at 0 °C, including the samples of Br-MOP and MeO-MOP (Fig. 4(b) and S38). There is a consistent trend in that all of the Route 2 samples show improved uptake when compared to Route 1 samples. We attribute the fact that this trend is not observed in the N_2_ measurements to differences in kinetics that arise from the significantly different temperatures at which the data are collected. Post-sorption PXRD diffraction showed that all of the cages retain the degree of crystallinity shown prior to the sorption measurements (Fig. S39). Although the crystallinity of the cages before and after the gas sorption experiments is high, we were unsuccessful in attempts to obtain the structure of the activated phases using 3D ED.

**Fig. 4:**
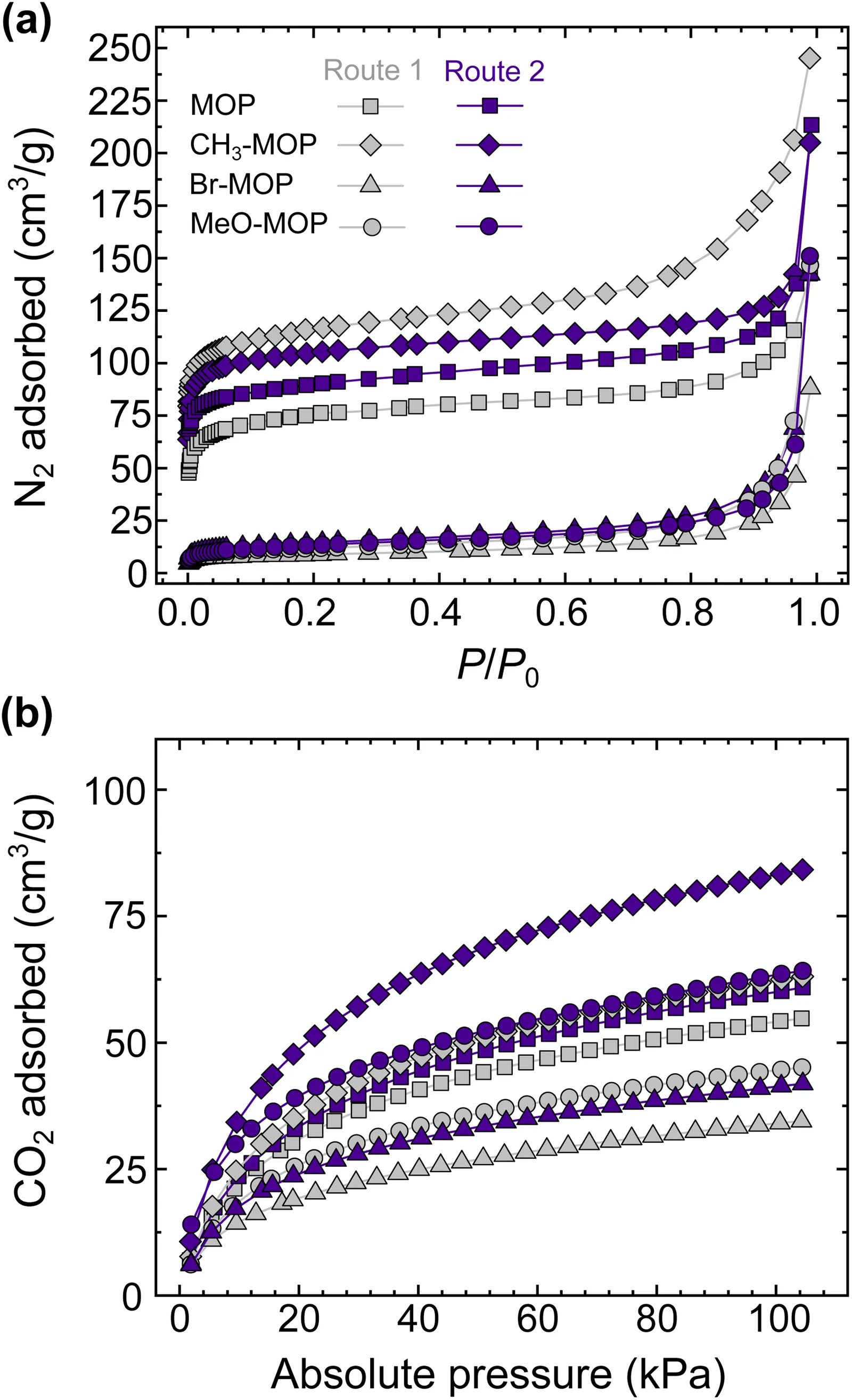
(a) N_2_ and (b) CO_2_ adsorption isotherms measured at −196 °C and 0 °C, respectively.

## Conclusions

In conclusion, we have developed distinct mechanochemical routes to the synthesis of porous metal–organic polyhedra, expanding the range of cage geometries that has been probed through mechanochemistry. In the first instance, we have been able to drastically reduce the volume of DMA required by 90% (Route 1) when compared to solvothermal synthesis. We have then shown it is possible to bypass the use of DMA completely, obtaining cage molecules by grinding the reactants in the solvent that would otherwise be used as the exchange solvent prior to gas sorption (here, MeOH). Due to the poor solubility of the reactants in this solvent, conventional solution-based synthesis to obtain these cages is not possible. Given that the solvation of MOPs influences their crystal packing, which, in turn, influences their gas sorption properties, these results suggest that the approach taken in Route 2 of this paper could be used to access a wide range of molecular packings that might not otherwise be observable. One possibility here would be to explore competitive liquid effects.^52^ Across all of these methods, we found mechanochemistry to yield particles that are at least as crystalline as samples obtained through conventional solvothermal routes, and in some cases significantly higher. This improved crystallinity and relatively small particle size allowed us to use 3D electron diffraction to determine the structures of the materials made in the absence of DMA, confirming the formation of discrete cages, in contrast to other MOPs, where solvent exchange can induce polymerisation.^51^ The gas sorption properties of these cages synthesised through different synthetic routes were compared, showing, in the case of MOP, an increase in BET surface area of the mechanochemical cages over the solvothermal cages; and for the other cages, comparable levels of gas uptake to samples obtained solvothermally. This paper reinforces the ease of mechanochemical synthesis in producing porous metal–organic materials, and underscores its environmental benefits in the synthesis of functional materials.

## Author contributions

MGW performed all synthetic procedures and collected IR, NMR, SEM, PXRD, and N_2_ and CO_2_ sorption data, as well as carrying out initial data interpretation. CSS, JPT, and SP collected and interpreted the 3D electron diffraction data. MRW collected and fitted PXRD data in capillary. AJF proposed suitable models and supervised the interpretation of the gas sorption data. GAC and AJF conceived and supervised the project. All authors discussed the results and commented on the drafts of the manuscript written by MGW and GAC.

## Conflicts of interest

There are no conflicts to declare.

## Supplementary Material

SC-017-D6SC02926D-s001

SC-017-D6SC02926D-s002

## Data Availability

CCDC 2523507 and 2523508 contain the supplementary crystallographic data for this paper.^58*a*,*b*^ The raw data that support the findings of this study are openly available from the University of Strathclyde KnowledgeBase at https://doi.org/10.15129/d303db24-341b-45ff-a35d-45bd6dd84bbc. Supplementary information (SI): detailed synthetic and physical characterisation procedures; additional spectroscopic and crystallographic data; and additional gas sorption data and analyses. See DOI: https://doi.org/10.1039/d6sc02926d.
