# Supplementary material for: Developing efficient mechanochemical routes to porous metal–organic polyhedra, supported by 3D electron diffraction
Source: Chem Sci. 2026 Aug 3;17(37):17815–23. doi: 10.1039/d6sc02926d (PMC13454935; doi:10.1039/d6sc02926d)
Supplement: Supplementary file 1 [file SC-017-D6SC02926D-s001.pdf]

Supplementary Information (SI) for Chemical Science.
This journal is © The Royal Society of Chemistry 2026

Developing efficient mechanochemical routes to porous metal-organic polyhedra, supported by 3D electron diffraction.

Megan G. Wilkinson,[1] Calum S. Sangster,[2] Jeremiah P. Tidey,[3] Martin R. Ward,[4] Simon Parsons,[2] Ashleigh J. Fletcher,[5] and Gavin A. Craig[1]*

[1]Department of Pure and Applied Chemistry, University of Strathclyde, Glasgow, United Kingdom, G1 1RX. gavin.craig@strath.ac.uk

[2]EaStCHEM, School of Chemistry, University of Edinburgh, Edinburgh, United Kingdom, EH9 3FJ.

[3]Department of Physics, University of Warwick, Gibbet Hill Road, Coventry, United Kingdom, CV4 7AL.

[4]Centre for Continuous Manufacturing and Advanced Crystallisation (CMAC), University of Strathclyde, Glasgow, United Kingdom, G1 1XW.

[5]Department of Chemical and Process Engineering, University of Strathclyde, Glasgow, United Kingdom, G1 1XJ.

Experimental details

Reagents were bought from commercial sources and used without further purification. The ligands LH_2, CH_3LH_2, MeO-LH_2 and $BrLH_2$ were synthesised following previously reported protocols, which were also followed to obtain reference data for solvothermally-synthesised materials.[1,2]

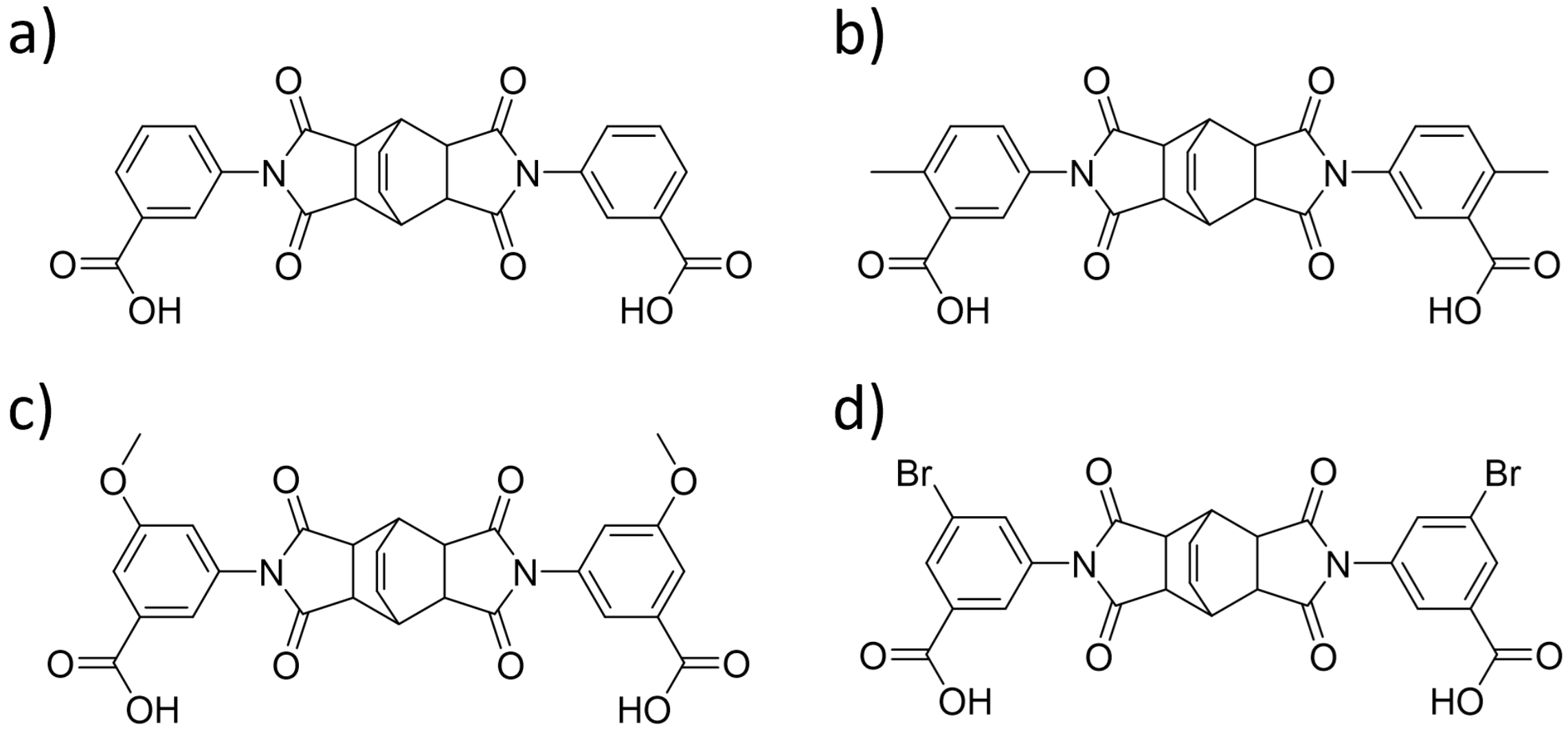

Fig. S1. Chemical structures of ligands LH_2, CH_3LH_2, MeO-LH_2 and $BrLH_2$ in **a**-**d**), respectively.

Synthesis.

One of the bicyclo[2.2.2]oct-7-ene ligands, LH_2, CH_3LH_2, MeO-LH_2 or $BrLH_2$ was mixed with $Cu(OAc)_2{\cdot}H_2O$ in a 1:1 molar ratio in a 2 mL Eppendorf tube with a given volume of dimethylacetamide (DMA) (Route 1, as shown in Fig. 1 of the main manuscript) or MeOH (Route 2, as shown in Fig. 1 of the main manuscript) and a 5 mm stainless steel ball. Detailed quantities are provided in Table S1. The Eppendorf tube was mounted on a Retsch Mixer Mill MM400 and milled at a frequency of 30 Hz for 60 minutes. The resulting materials were allowed to dry in air overnight to obtain blue solids, prior to characterisation by powder X-ray diffraction; infra-red spectroscopy; and NMR spectroscopy of acid-digested samples. To obtain the solvent-exchanged phases, these powders were soaked in methanol (MeOH), which was replaced with fresh solvent approximately 10 times over the course of 5 days.

Nomenclature for compounds.

For compounds synthesised using Route 1 in Fig. 1 of the main manuscript, the compounds are labelled **X-MOP**$^{R1}_{\eta}$, where X denotes the ligand used, and η is either slurry, LAG for liquid-assisted grinding, or NG for neat grinding. The corresponding values of η are given in Table S1. The samples obtained after

the MeOH exchange step have MeOH added in the subscript, e.g. **CH_3-MOP$^{R1}_{slurry, MeOH}$**. Compounds synthesised using Route 2 – grinding with MeOH – are labelled **X-MOP^{R2}** and are all slurries.

Table S1: Details of quantities used in the mechanochemical procedures described.

Sample	Ligand (mg)	$Cu(OAc)_2{\cdot}H_2O$ (mg)	Solvent	Solvent (μL)	*η*
**MOP$^{R1}_{slurry}$**	240	100	DMA	625	1.84
**CH_3-MOP$^{R1}_{slurry}$**	260	100	DMA	625	1.74
**MeO-MOP$^{R1}_{slurry}$**	270	100	DMA	625	1.69
**Br-MOP$^{R1}_{slurry}$**	320	100	DMA	625	1.49
**MOP$^{R1}_{NG}$**	98	40	n/a	0	0
**CH_3-MOP$^{R1}_{NG}$**	103	40	n/a	0	0
**MeO-MOP$^{R1}_{NG}$**	109	40	n/a	0	0
**Br-MOP$^{R1}_{NG}$**	129	40	n/a	0	0
**MOP$^{R1}_{LAG}$**	98	40	DMA	15	0.11
**CH_3-MOP$^{R1}_{LAG}$**	103	40	DMA	15	0.10
**MeO-MOP$^{R1}_{LAG}$**	109	40	DMA	15	0.10
**Br-MOP$^{R1}_{LAG}$**	129	40	DMA	15	0.09
**MOP^{R2}**	240	100	MeOH	625	1.84
**CH_3-MOP^{R2}**	260	100	MeOH	625	1.74
**MeO-MOP^{R2}**	270	100	MeOH	625	1.69
**Br-MOP^{R2}**	320	100	MeOH	625	1.49

Physical characterisation.

Fourier transform infra-red (FT-IR) spectroscopy was performed on neat samples using a Thermo Scientific spectrometer, model NICOLET iS5, with 48 scans and a resolution of 4 cm^{-1}. Powder X-ray diffraction (PXRD) data were collected on a Bruker d8 Discover in $\theta/2\theta$ Bragg-Brentano reflection geometry using a Cu K_{α} source (λ = 1.54056 Å). The samples were prepared using a flat plate sample holder, with silicon grease and Kapton film (available in the Centre for Continuous Manufacturing and Advanced Crystallisation (CMAC), at the University of Strathclyde). Capillary PXRD data were collected on a Bruker d8 Discover in $\theta/2\theta$ Debye-Scherrer transmission geometry using a Cu source with Johanssen monochromator providing $K_{\alpha 1}$ radiation (λ = 1.54056 Å). The samples were lightly ground using an agate mortar and pestle before loading into 0.7 mm OD borosilicate glass capillary tubes and then flame sealed for data collection (available in CMAC). Data were collected over a 2ϑ range of 4-50°

2ϑ using a step size of 0.01°. Analysis of PXRD was performed using TOPAS-Academic.[3] ^{1}H nuclear magnetic resonance (NMR) spectra were obtained at room temperature using a Bruker AVANCE 400 NMR spectrometer at 400.13 MHz. Acid digestion experiments were performed by suspending *ca.* 10 mg of sample in 600 µL DMSO-d_6 before adding 20 µL DCl. Scanning electron microscopy (SEM) samples were prepared using a small amount of solid sample on a metal stud covered with a conductive carbon sticker. The studs were sputter coated with 20 nm of gold using a Leica Em ACE200 coating system under an Ar environment (available in CMAC). SEM images were taken using a JEOL JSM-IT100 with a Tungsten source operating at 15 or 20 kV and 60 mA, or a FEI Quanta 250 FEG-ESEM operating at 30 kV (available in the Department of Physics at the University of Strathclyde). Prior to gas sorption measurements, samples were activated *in situ* by heating at 120 °C for 10 hours under vacuum. N_2 uptake was measured at −196 °C using a Micromeritics ASAP 2420 (available in the Department of Chemical and Process Engineering at the University of Strathclyde), using oxygen free nitrogen gas at pressures from 0 to 760 mmHg. CO_2 uptake was measured at 0 °C, using a Micrometrics ASAP 2425 (available in the Department of Chemical and Process Engineering, at the University of Strathclyde), using pressures in the range 0 t0 760 mmHg.

3D electron diffraction

In all cases, blue powder samples were lightly ground between two glass slides and dispersed dry onto a copper-supported holey carbon TEM grid. These were loaded into the Rigaku XtaLAB Synergy-ED electron diffractometer using a Gatan Elsa model 698 high-tilt cryo holder and into the sample chamber at ~215 K to sublime the ice before cooling to collection temperatures of 100(5) K. The instrument was operated at 200 kV and equipped with a Rigaku HyPix-ED hybrid pixel array area detector.

Data for a range of crystallites were then surveyed using continuous rotation electron diffraction employing selected area apertures of ~1 or 2 µm diameter at the image plane using CrysAlisPRO (version 1.171.44.100a).[4] The reported data for are taken from merges of 12 and 9 viable and suitably matching datasets for **MOP**$^{\boldsymbol{R2}}$ and **CH_3-MOP**$^{\boldsymbol{R2}}$, respectively, from crystallites with dimensions largely in the order of 0.1–0.5 µm and using frame scan widths of 0.25°. The data were indexed, integrated, and scaled using CrysAlisPRO (version 1.171.44.134a)[4] and SCALE3 ABSPACK, implemented therein; no absorption corrections were applied. The results presented are representative of all specimens assessed, ranging up to regular blocks of dimensions in excess of 1 µm.

The structures were solved using ShelXT[5] and refined in the kinematic approximation, employing extinction corrections to broadly account for the effects of multiple scattering, using Olex2.refine[6] as

implemented in Olex2 (version 1.5-ac7-020, compiled 2025.12.04 svn.r37a0dd52 for Rigaku Oxford Diffraction, GUI svn.r7421)[7] and published scattering factors.[8]

Both refinements proceeded well to convergence. Chemically equivalent bonds were refined in both models with distance similarity restraints (SADI). All non-H atoms of the main molecules were treated as having anisotropic displacements with rigid bond restraints (RIGU) to better model displacement parameters and provide resilience to multiple scattering effects; in **MOP*R2***, a small region of disorder is modelled in the ligand, requiring additional proximal similarity restraints on the ADPs. C-H hydrogen atoms are placed at geometrically constrained positions at neutron bond distances. Pore methanol C-O distances employ set distance restraints (DFIX) and equivalent displacement parameters (EADP) per molecule while additional proximal similarity restraints (SIMU) are applied over the whole content. Methanolic protons and those for Cu-bound waters internal with respect to the cage pore are also set geometrically at neutron distances; external Cu-bound waters are refined in the presence of 1,2 and 1,3-distance similarity restraints (SADI) which include the constrained sites. Plausible H-atoms positions could be defined only for some atoms of the molecules comprising the pore content. Refinement of adps for all atoms in the pores also proved to be unstable, and modelling of the displacement parameters is at the isotropic level in some cases. All hydrogen atoms were modelled with isotropic displacement parameters equal to 1.5 or 1.8 times the equivalent isotropic displacement parameter of their parent atoms.

Additionally, we report experimental data for **MeO-MOP*R2*** and **Br-MOP*R2***, taken from merges of 2 and 4 datasets, for which the experiment, integration and merging were performed in the same manner as above. These specimens diffracted very poorly, yielding weak data with resolution barely exceeding 2.0 Å *d*-spacing. In both cases, the unit cell parameters and volume are broadly consistent with those of the other two phases, indicating they may form similar phases. For **MeO-MOP*R2***, isomorphous replacement adapting the model from **MOP*R2*** supports this inference (approx. stats: R_1 ~35% for 63% complete data at d_{min} 1.78 Å), but we note that all efforts to do so with **Br-MOP*R2*** fail to provide any reasonable starting fit (*i.e.*, R_1 >>50%) despite testing for potential apparent switching of relative cell axes and the data having similar global statistics.

Complete experimental and refinement information for **MOP*R2*** and **CH_3-MOP*R2*** are contained within the deposited CIF along with structure factors and an embedded .RES file. These are deposited in the CSD with CCDC reference codes CCDC 2523507-2523508. Experimental information, including structure factors, and embedded .RES for the tested isomorphous replacement solutions for **MeO-MOP*R2*** and **Br-MOP*R2*** are additionally made available with the SI for completeness. Experimental and refinement parameters are reported in Table S2.

Table S2. Experimental data table for all merged 3D ED experiments, and key refinement metrics for the reported structures of **MOP^{R2}** and **CH_3-MOP^{R2}** and that tested via isomorphous replacement for **MeO-MOP^{R2}** and **Br-MOP^{R2}**.

**Crystal data**				
**Compound**	**MOP^{R2}**	**CH_3-MOP^{R2}**	**MeO-MOP^{R2}**	**Br-MOP^{R2}**
**CCDC deposition**	2523507	2523508	*N.A.*	*N.A.*
**Chemical Formular (nominal)**	$Cu_4C_{104}H_{72}N_8O_{36}\cdot 8(H_2O)\cdot MeOH$	$Cu_4C_{112}H_{88}N_8O_{36}\cdot 6.271(H_2O)\cdot 3.729(MeOH)$	$Cu_4C_{112}H_{88}N_8O_{44}$	$C_{104}H_{50}Br_8Cu_4N_8O_{34}$
**Chemical Formular (as refined)**	$Cu_4C_{104}H_{72}N_8O_{36}\cdot 8(O)\cdot CO$	$Cu_4C_{112}H_{88}N_8O_{36}\cdot 6.271(O)\cdot 2(CH4O)\cdot 1.729(CO)$	$Cu_4C_{112}H_{88}N_8O_4$	*N.A.*
**M_r (as refined)**	2419.93	2589.00	2540.13	*N.A.*
**Crystal system, space group**	Triclinic, *P*−1	Triclinic, *P*−1	Triclinic, *P*−1	Triclinic, *N.A.*
**Temperature (K)**	100(5)	100(5)	100(5)	100(5)
***a*, *b*, *c* (Å)**	13.2027(13), 14.2806(11), 16.1888(13)	13.6461(11), 14.0771(17), 17.0238(13)	13.71(4), 14.71(3), 16.75(4)	15.182(18), 14.94(3), 16.23(3)
**α, β, γ (°)**	108.604(8), 107.689(11), 101.874(8)	100.785(8), 111.770(8), 103.521(9)	99.94(19), 109.6(3), 102.8(2)	101.24 (17), 113.95(14), 109.41(14)
***V* (Å^3)**	2596.5(5)	2813.8(6)	2988(15)	2932(13)
**Z**	1	1	1	*N.A.*
**Radiation type**	Electron, λ = 0.0251 Å			
**Data collection**				
**No. component datasets**	12	9	2	4
**Scan range (°)**	See deposited or SI CIFs		See CIFs in SI deposition	
**No. reflns (meas., indep., obs.)***	70647, 5643, 3439	62287, 6944, 4121	1951, 836, 260	2992, 662, 242
**R_{int}**	0.253	0.251	0.418	0.449
**Resolution ($(\sin(\vartheta/\lambda)_{max}$ (Å^{-1}), *d*-spacing (Å))**	0.521, 0.96	0.542, 0.92	0.278, 1.78	0.278, 1.78
**Refinement**				
**R_1, wR_2 ([F^2 >2σ(F^2)])**	0.1574, 0.3848	0.1459, 0.3433	0.3565, 0.6169	
**R_1, wR_2 (all)**	0.2117, 0.4174	0.2029, 0.3775	0.4236, 0.6742	
**GoF(*S*) (inc., excl. restraints)**	1.0074, 1.0986	1.0239, 1.0954	1.0251, 1.9837	
**No. reflns indep., obs., reject., completeness**	5541, 3408, 102, 90.3%	6839, 4072, 105, 90.0%	668, 254, 176, 61.4%	*N.A.*
**No. parameters**	748	826	344	
**No. restraints**	908	854	367	
**$\Delta\varphi_{max}$, $\Delta\varphi_{min}$ (eÅ^{-1})**	1.21, −0.79	1.03, −0.81	1.40, −1.24	

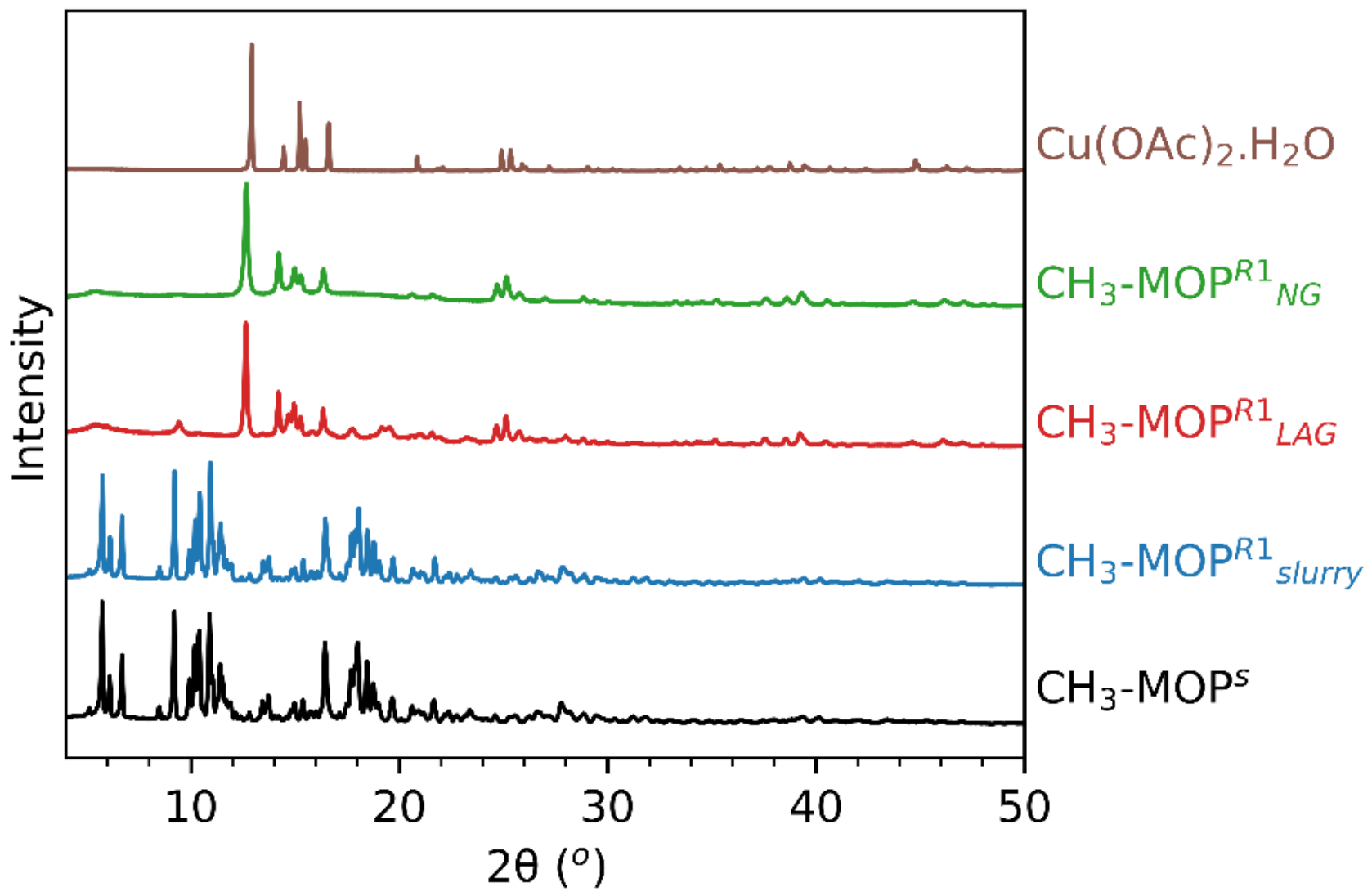

Fig. S2. Comparison of the PXRD data obtained for the various mechanochemical regimes of **CH_3-MOP** with the diffractogram for copper(II) acetate, proving the failure of the LAG and neat grinding syntheses.

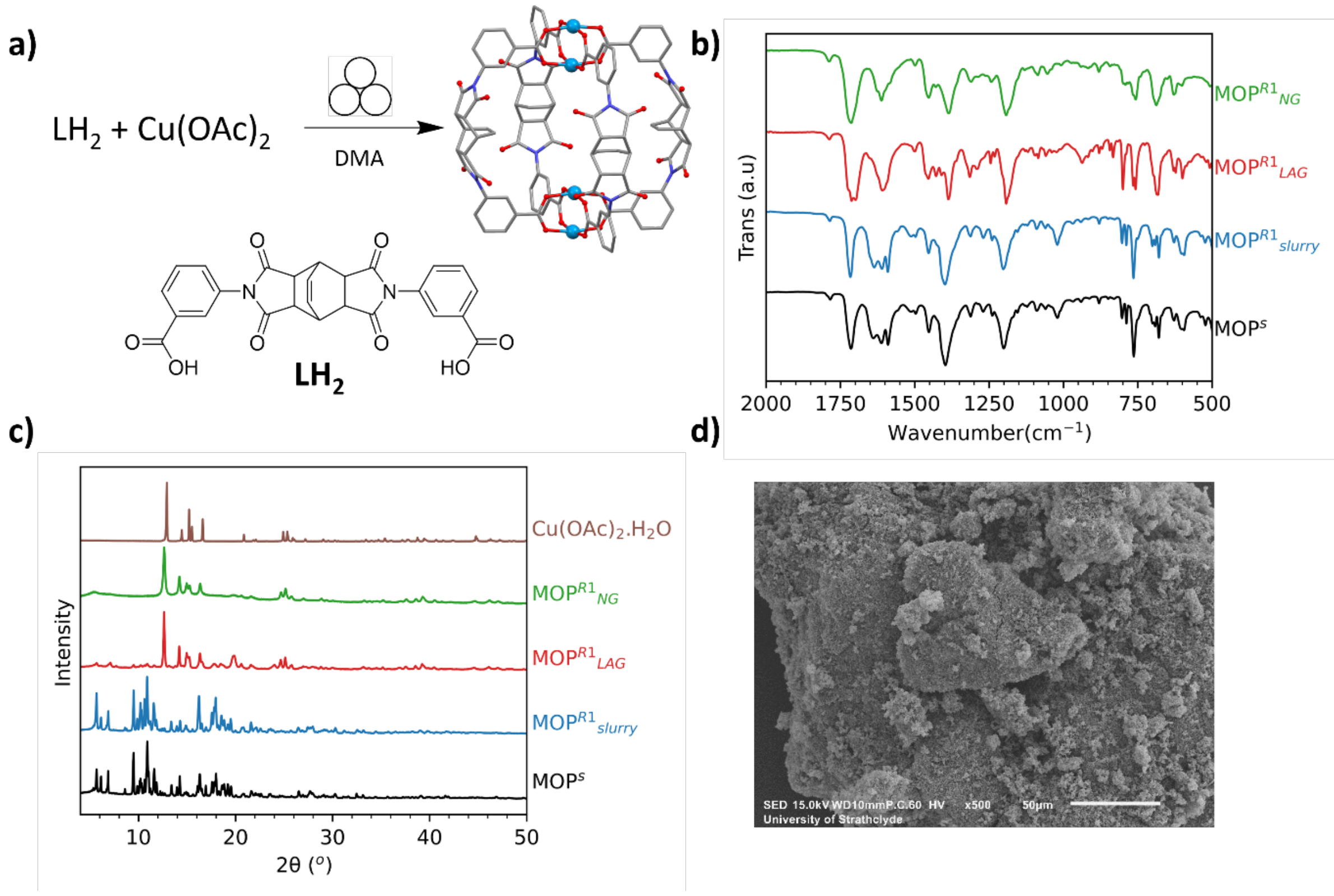

Fig. S3. (a) Reaction scheme showing the mechanochemical approach, with the intended product structure taken from the .cif available in the CSD[1] and structure of the ligand **LH_2**. (b) and (c) IR spectra and PXRD data, respectively, for **MOP^{S}**, **MOP^{R1}$_{slurry}$**, **MOP^{R1}$_{LAG}$**, **MOP^{R1}$_{NG}$**, (d) SEM image of the product obtained from mechanochemical synthesis of a slurry, **MOP^{R1}$_{slurry}$**.

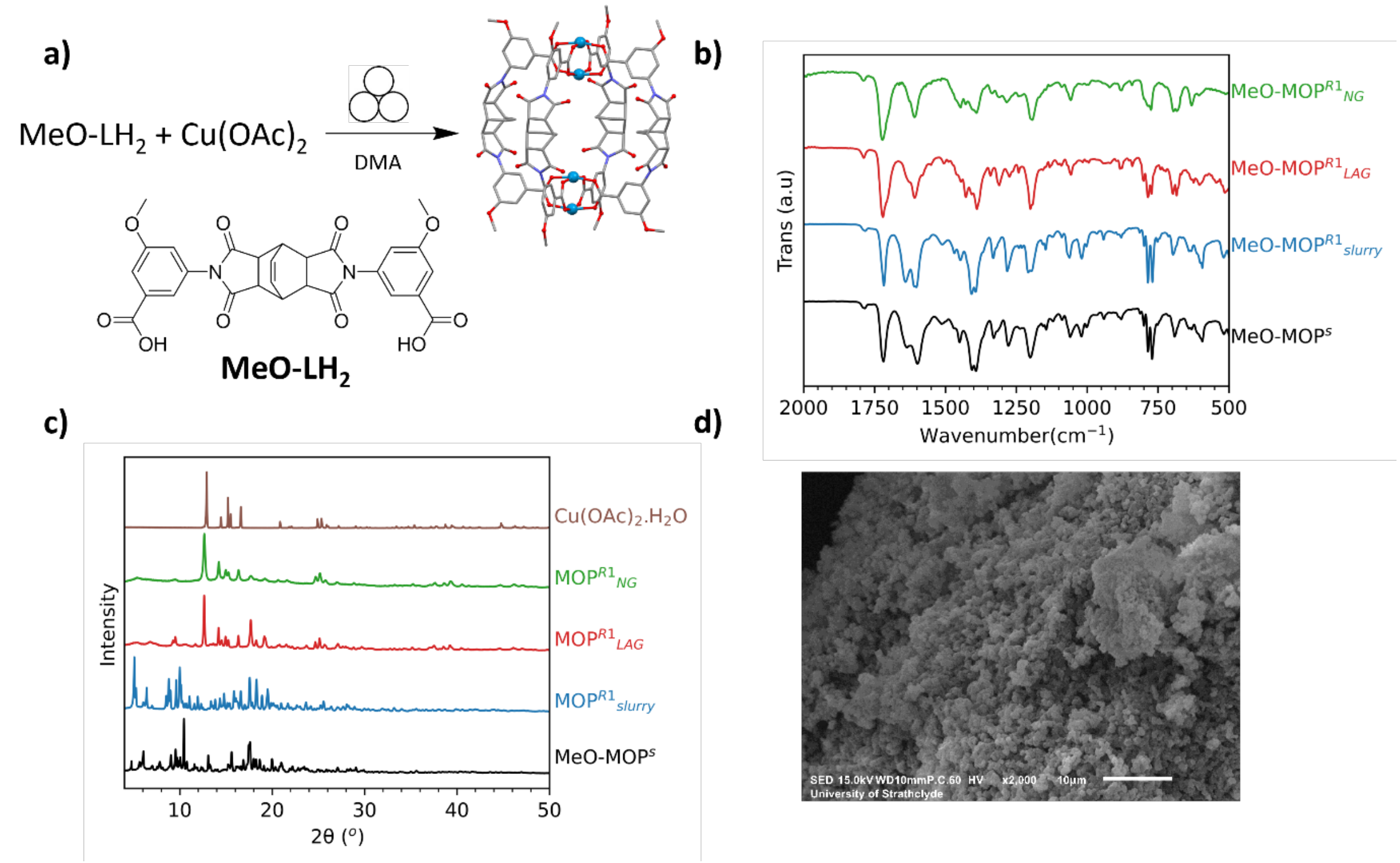

Fig. S4. (a) Reaction scheme showing the mechanochemical approach, with the intended product structure taken from the .cif available in the CSD[2] and structure of the ligand **MeO-LH$_2$**. (b) and (c) IR spectra and PXRD data, respectively, for **MeO-MOP^{S}**, **MeO-MOP$^{R1}_{slurry}$**, **MeO-MOP$^{R1}_{LAG}$**, **MeO-MOP$^{R1}_{NG}$**, (d) SEM image of the product obtained from mechanochemical synthesis of a slurry, **MeO-MOP$^{R1}_{slurry}$**.

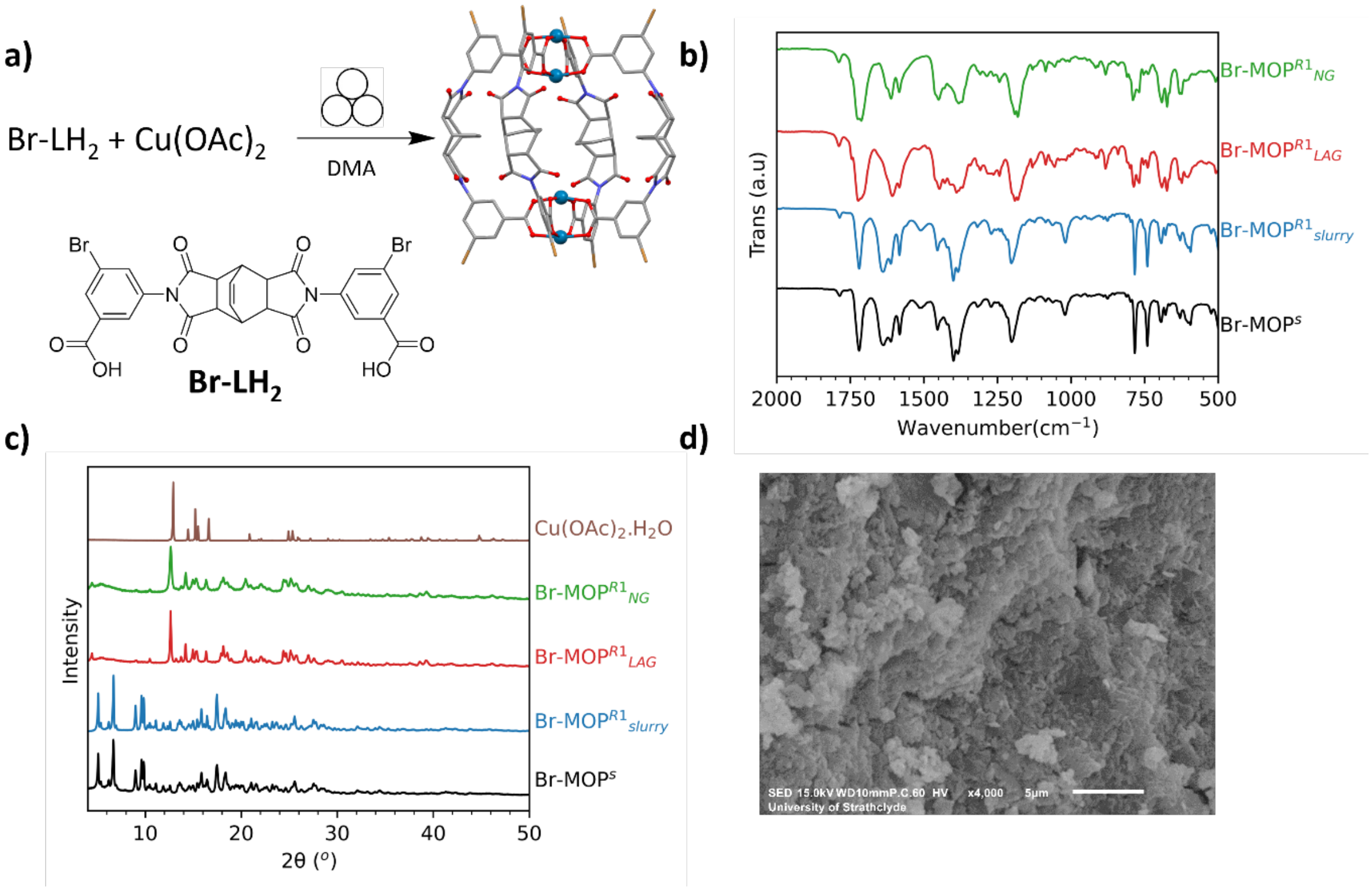

Fig. S5. (a) Reaction scheme showing the mechanochemical approach, with the intended product structure taken from the .cif available in the CSD[2] and structure of the ligand **Br-LH₂**. (b) and (c) IR spectra and PXRD data, respectively, for **Br-MOP^{S}**, **Br-MOP$^{R1}_{slurry}$**, **Br-MOP$^{R1}_{LAG}$**, **Br-MOP$^{R1}_{NG}$**, (d) SEM image of the product obtained from mechanochemical synthesis of a slurry, **Br-MOP$^{R1}_{slurry}$**.

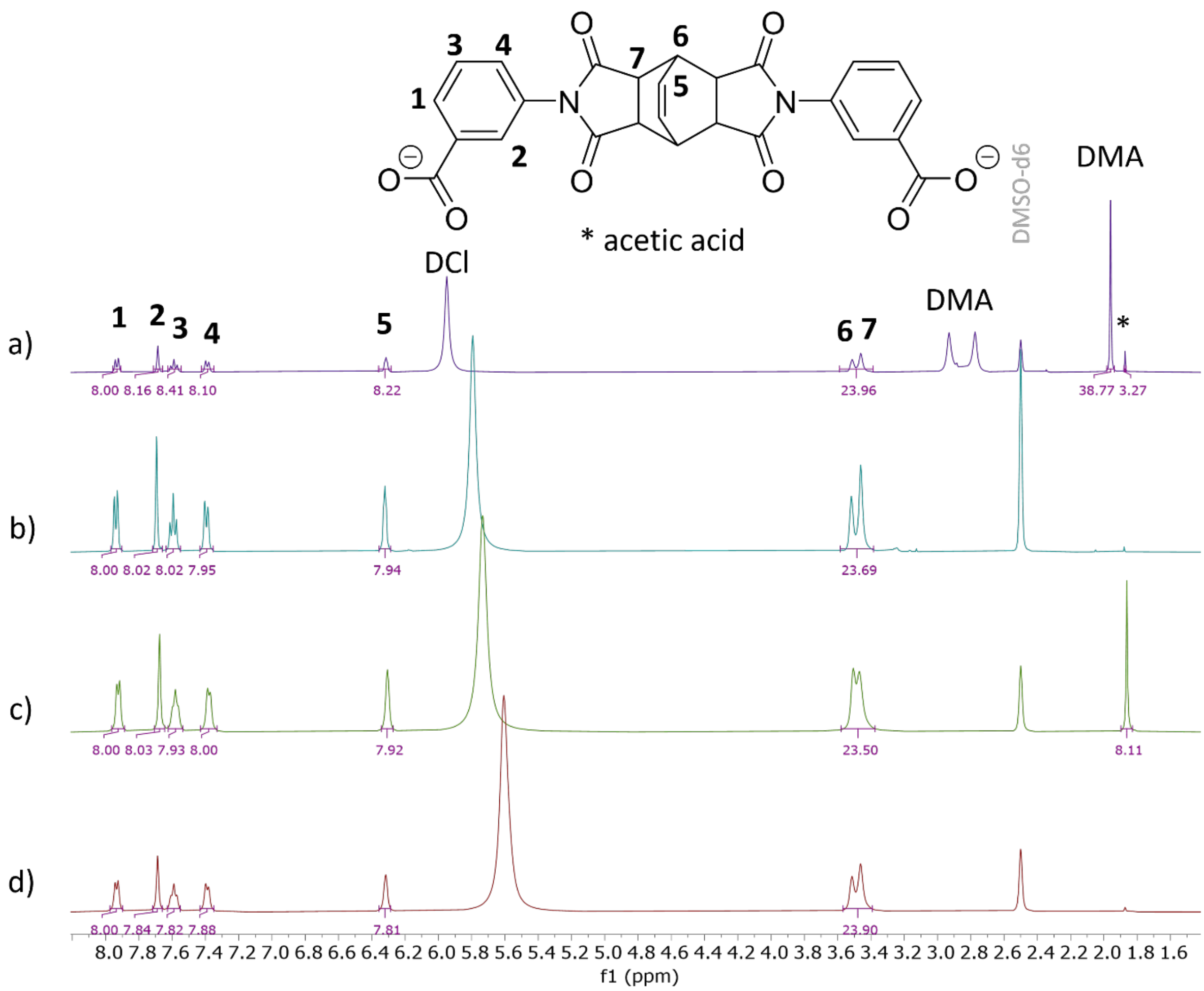

Fig. S6. NMR digestion spectra for a) $\mathbf{MOP^{R1}_{slurry}}$, b) $\mathbf{MOP^{R1}_{slurry, MeOH}}$, c) $\mathbf{MOP^{R2}}$ and d) $\mathbf{MOP^{R2}_{MeOH}}$ showing ligand peaks, and DCl (from digestion).

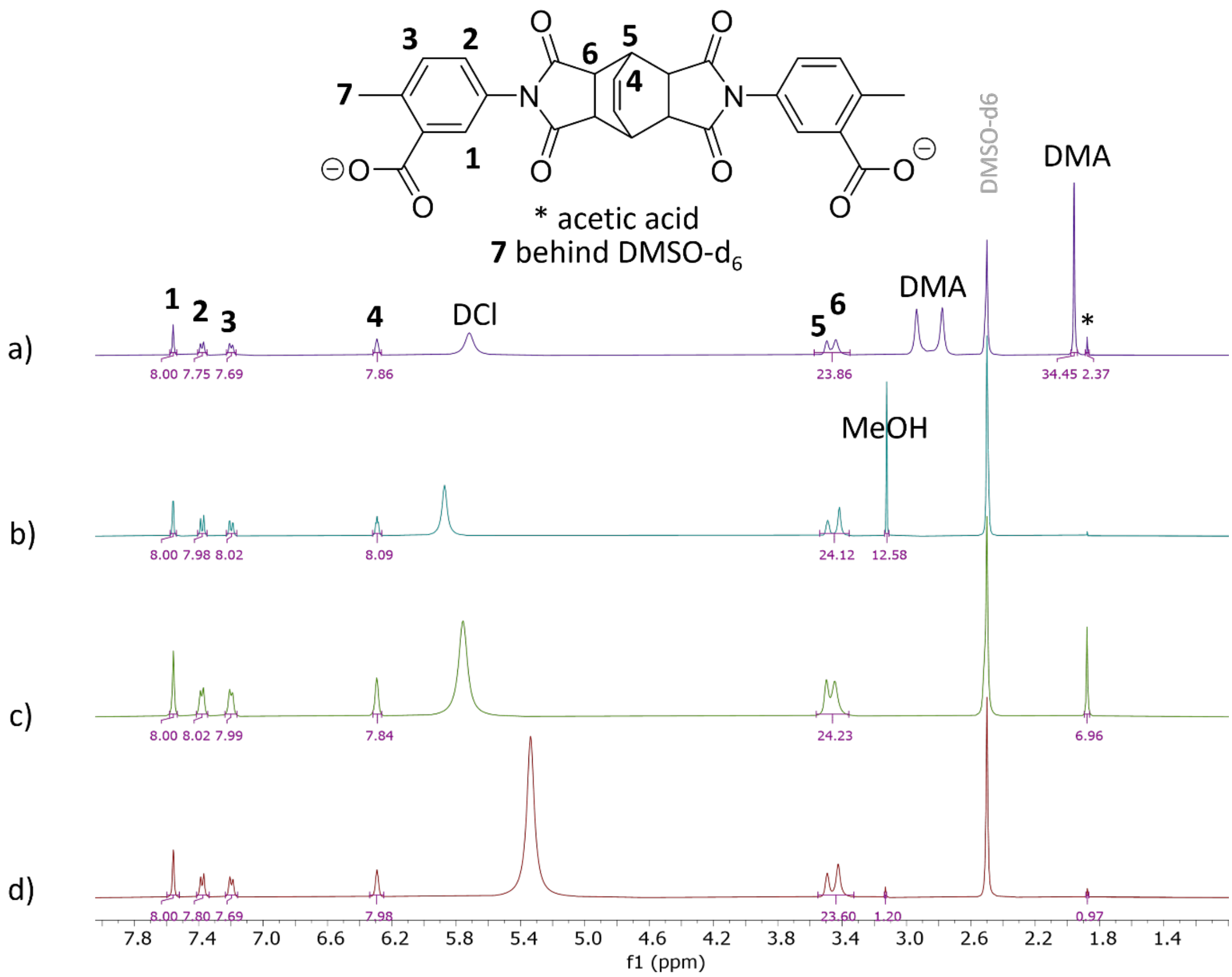

Fig. S7. NMR digestion spectra for a) **CH_3-MOP^{R1}$_{slurry}$**, b) **CH_3-MOP^{R1}$_{slurry,\ MeOH}$**, c) **CH_3-MOP^{R2}** and d) **CH_3-MOP^{R2}$_{MeOH}$** showing ligand peaks, and DCl (from digestion).

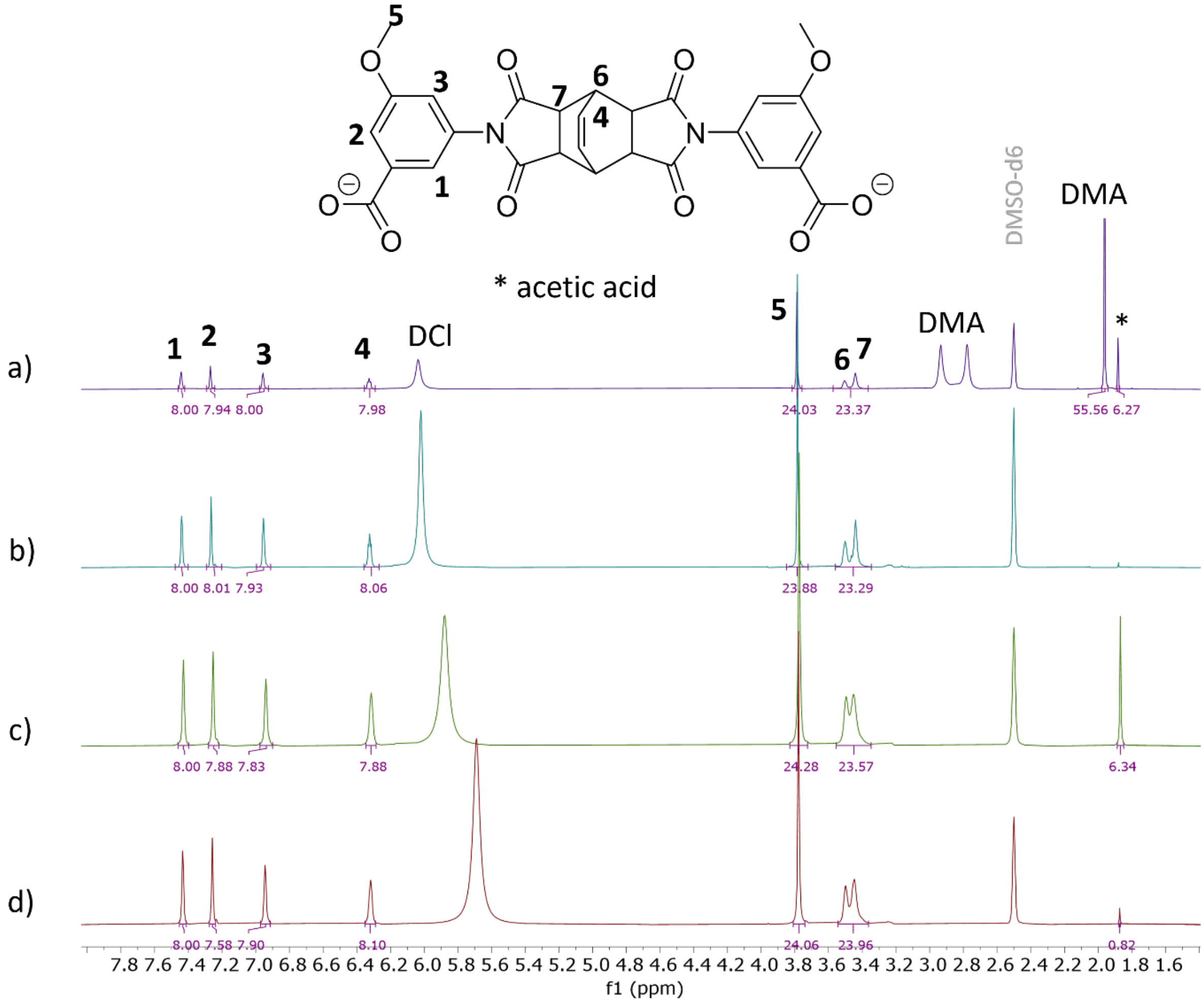

Fig. S8. NMR digestion spectra for a) **MeO-MOP^{R1}$_{slurry}$**, b) **MeO-MOP^{R1}$_{slurry, MeOH}$**, c) **MeO-MOP^{R2}** and d) **MeO-MOP^{R2}$_{MeOH}$** showing ligand peaks, and DCl (from digestion).

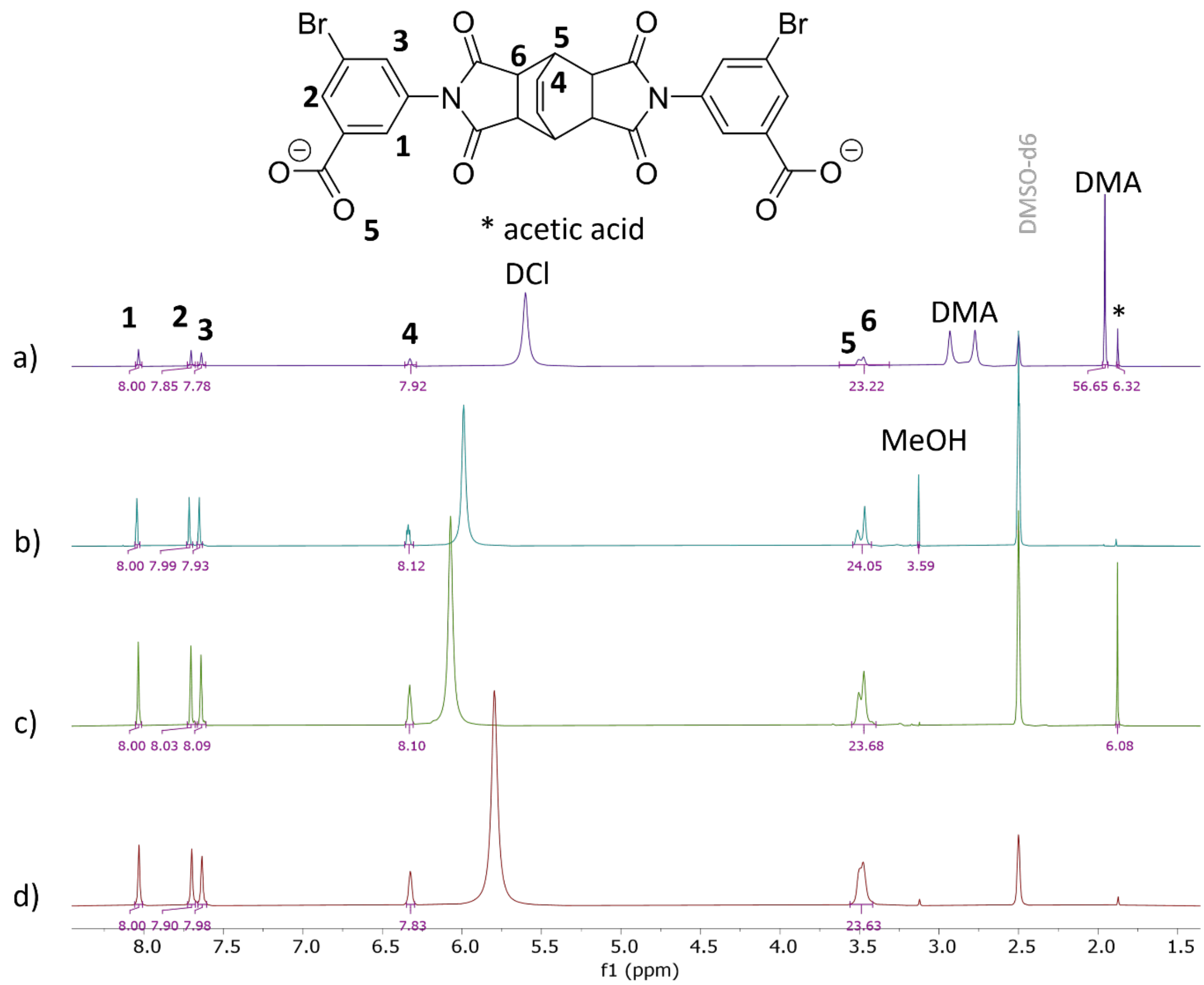

Fig. S9. NMR digestion spectra for a) **Br-MOP^{R1}$_{slurry}$**, b) **Br-MOP^{R1}$_{slurry, MeOH}$**, c) **Br-MOP^{R2}** and d) **Br-MOP^{R2}$_{MeOH}$** showing ligand peaks, and DCl (from digestion).

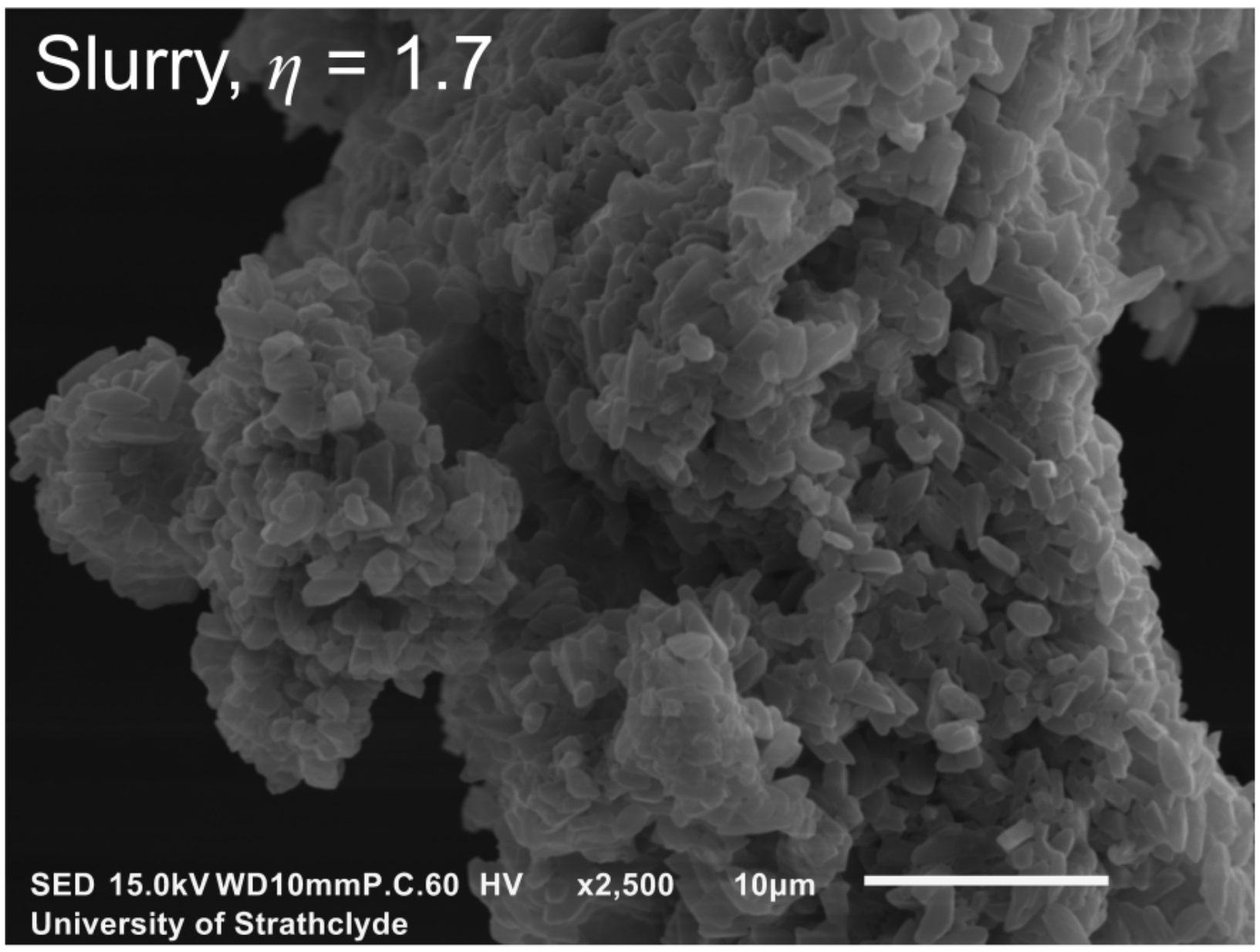

Fig. S10. SEM image of the sample **CH_3-MOP^{R1}$_{slurry}$**.

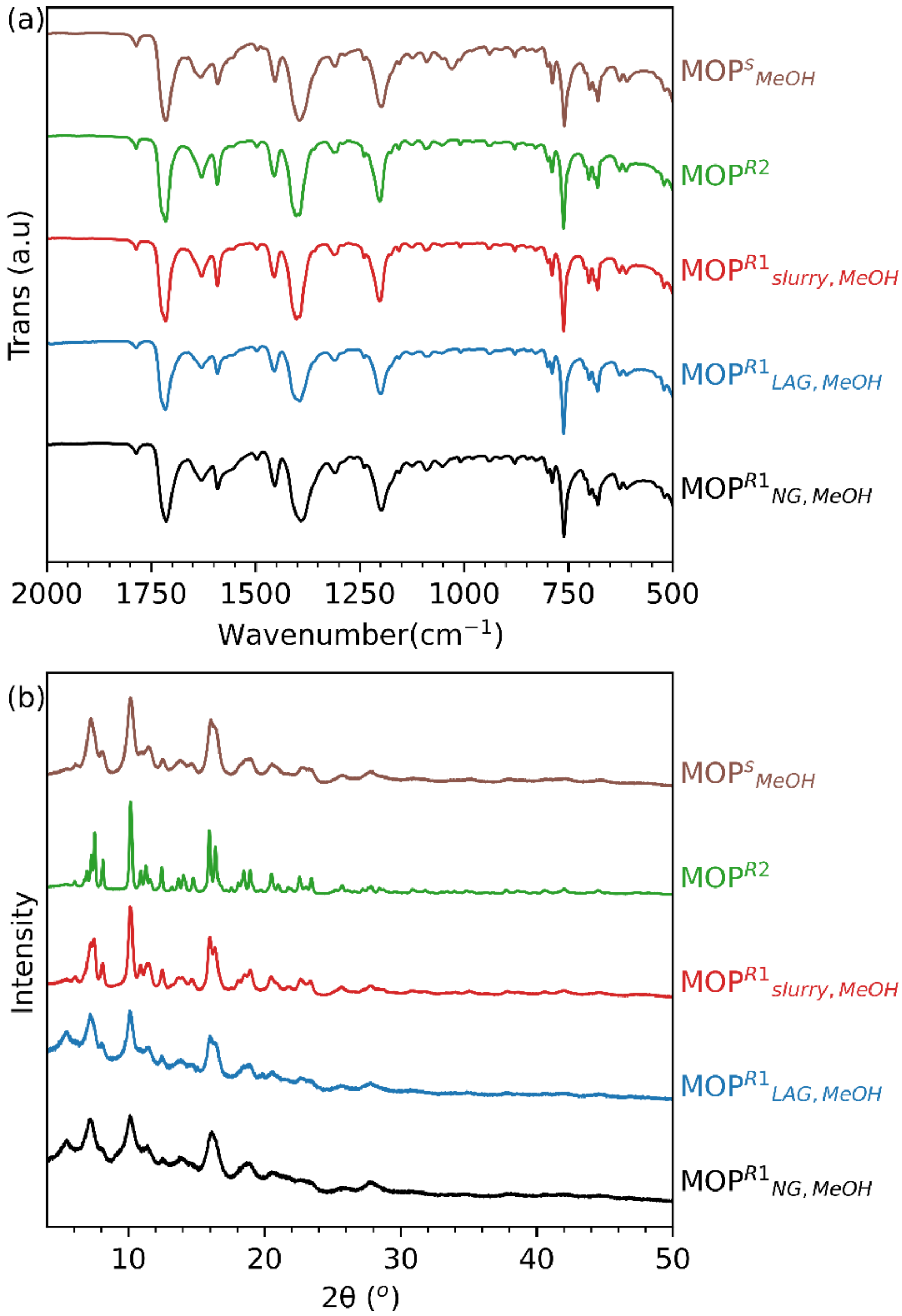

Fig. S11. a) FT-IR spectra and b) PXRD diffractograms for **MOP^{R1}**$_{NG,MeOH}$, **MOP^{R1}**$_{LAG,MeOH}$, **MOP^{R1}**$_{slurry,MeOH}$, **MOP^{R2}** and **MOP^{S}**$_{MeOH}$, showing the same product is produced from all MeOH routes, including NG and LAG samples, where MOP forms upon soaking in MeOH.

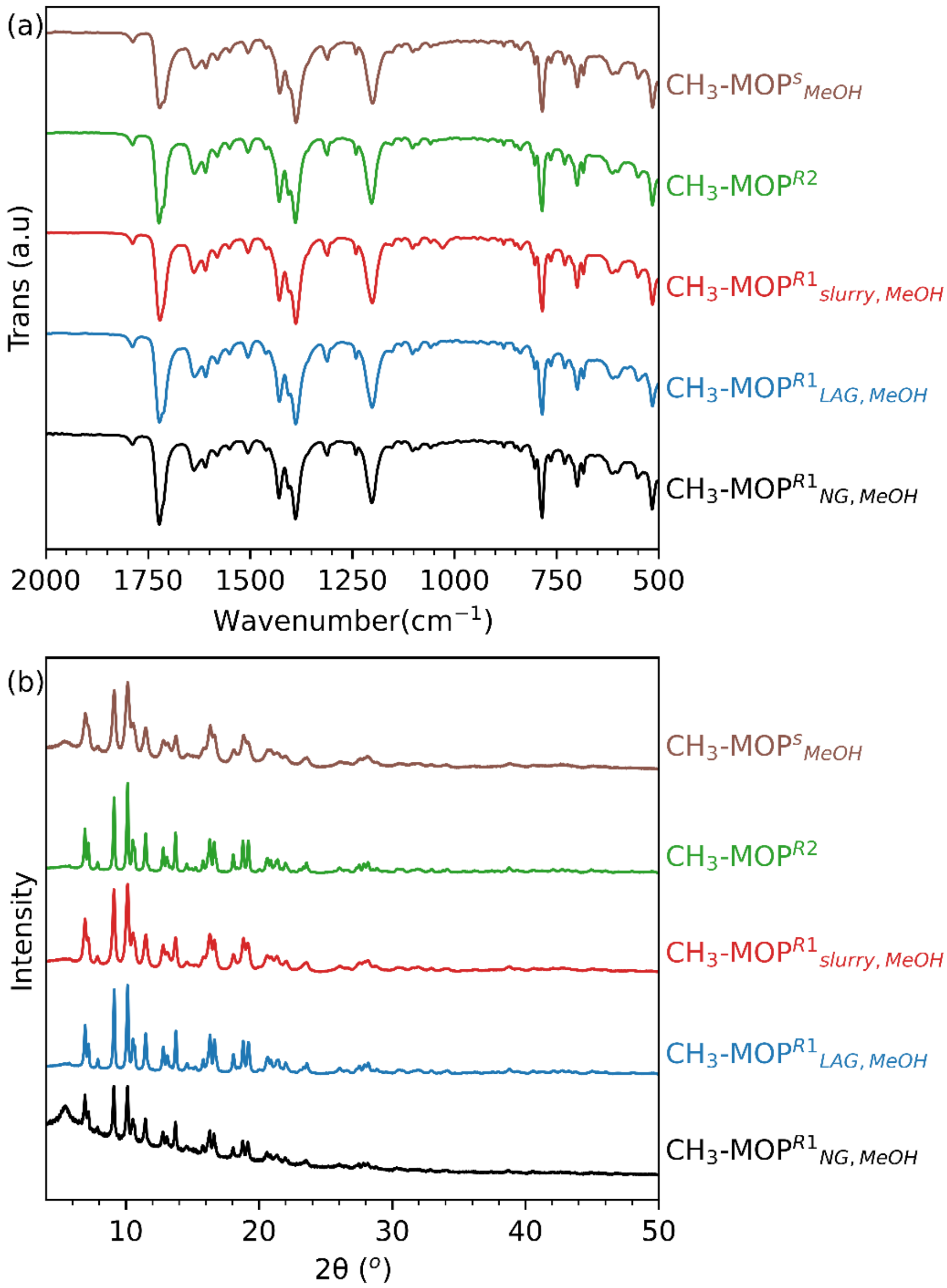

Fig. S12. a) FT-IR spectra and b) PXRD diffractograms for **CH_3-MOP$^{R1}_{NG,MeOH}$**, **CH_3-MOP$^{R1}_{LAG,MeOH}$**, **CH_3-MOP$^{R1}_{slurry,MeOH}$**, **CH_3-MOP^{R2}** and **CH_3-MOP$^{S}_{MeOH}$**, showing the same product is produced from all MeOH routes, including N and LAG samples, where CH_3-MOP forms upon soaking in MeOH.

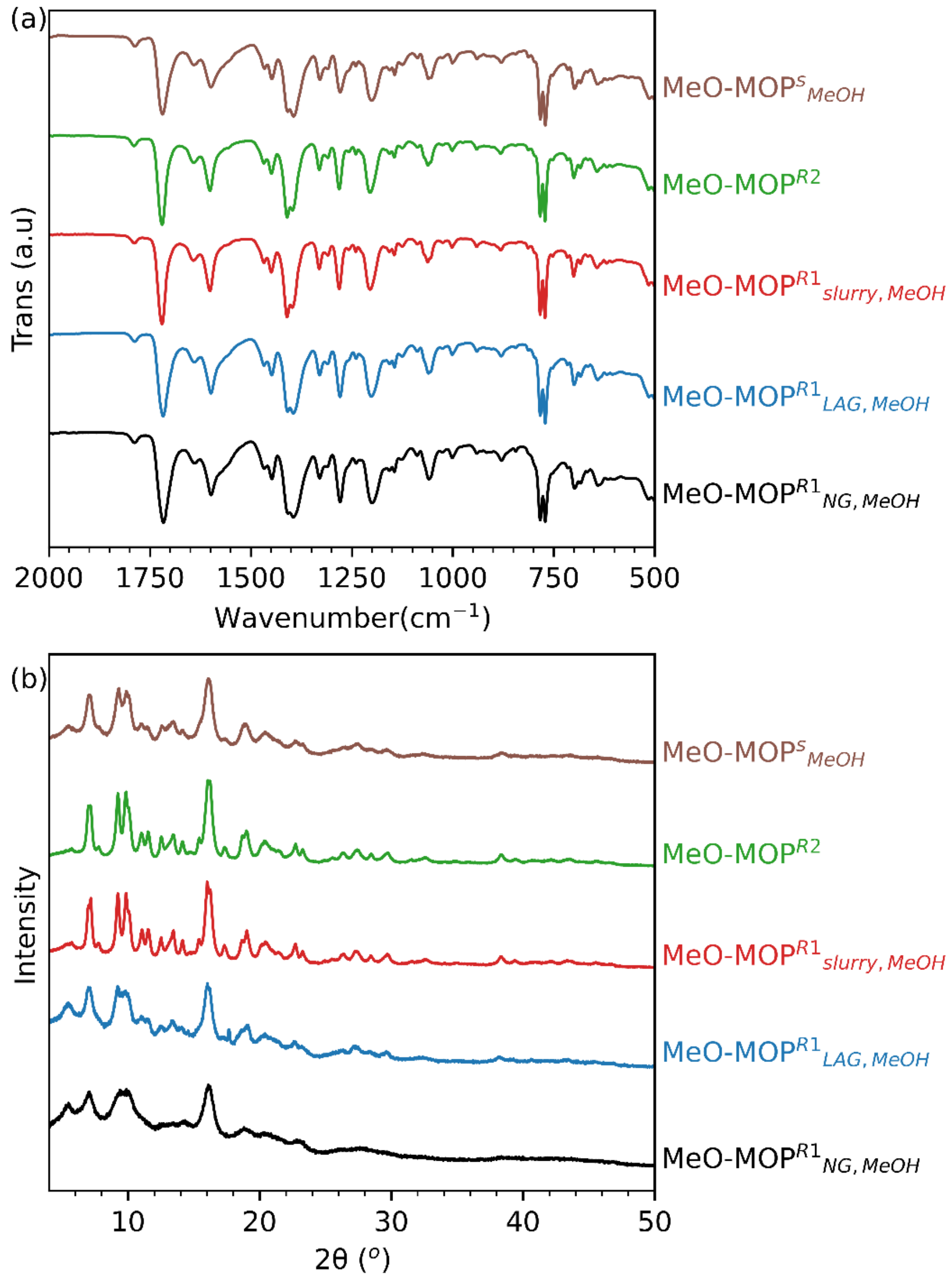

Fig. S13. a) FT-IR spectra and b) PXRD diffractograms for **MeO-MOP$^{R1}_{NG,MeOH}$**, **MeO-MOP$^{R1}_{LAG,MeOH}$**, **MeO-MOP$^{R1}_{slurry,MeOH}$**, **MeO-MOP^{R2}** and **MeO-MOP$^{S}_{MeOH}$**, showing the same product is produced from all MeOH routes, including N and LAG samples, where MeO-MOP forms upon soaking in MeOH.

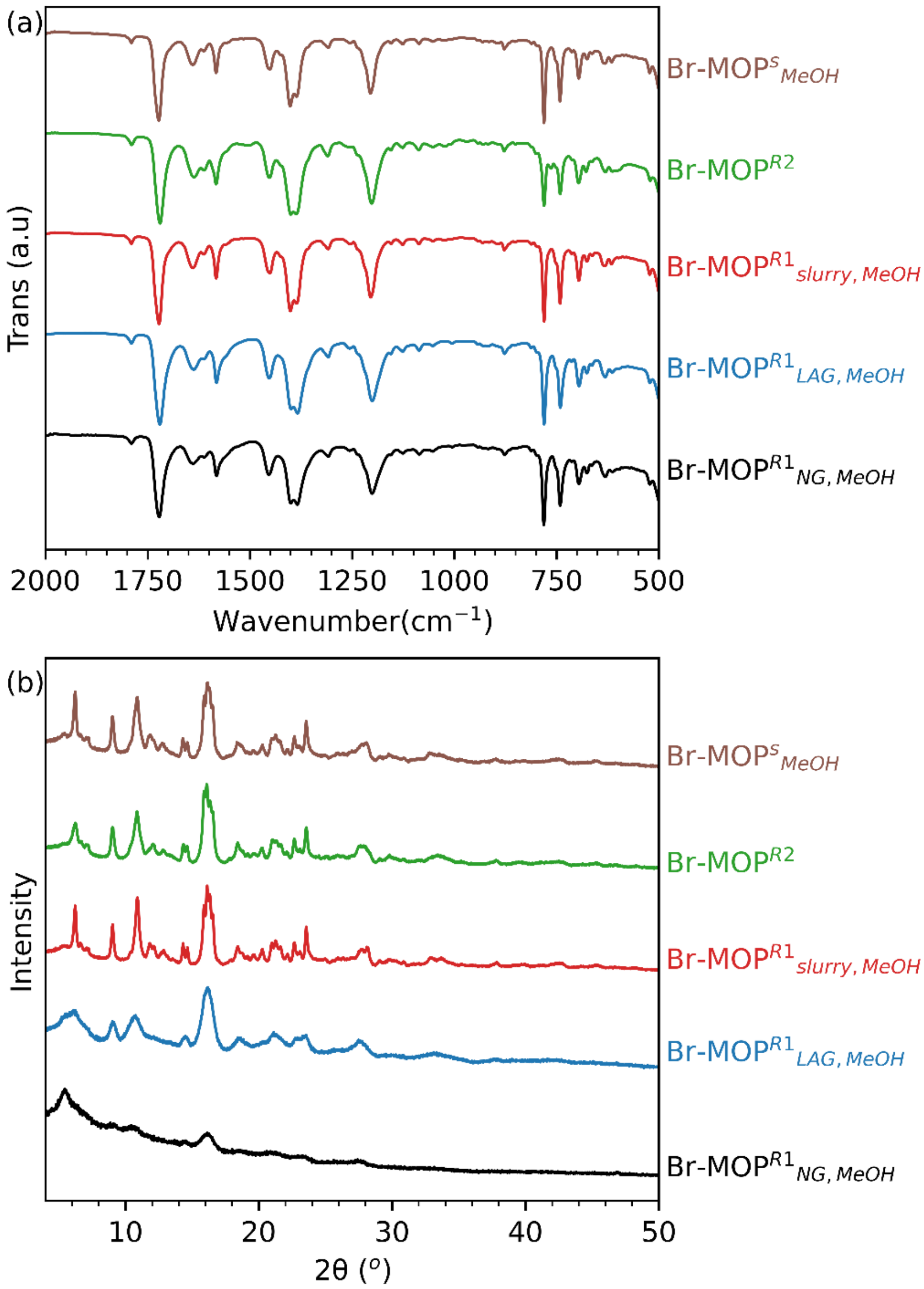

Fig. S14. a) FT-IR spectra and b) PXRD diffractograms for **Br-MOP^{R1}**$_{NG,MeOH}$, **Br-MOP^{R1}**$_{LAG,MeOH}$, **Br-MOP^{R1}**$_{slurry,MeOH}$, **Br-MOP^{R2}** and **Br-MOP^{S}**$_{MeOH}$, showing the same product is produced from all MeOH routes, including N and LAG samples, where Br-MOP forms upon soaking in MeOH including N and LAG

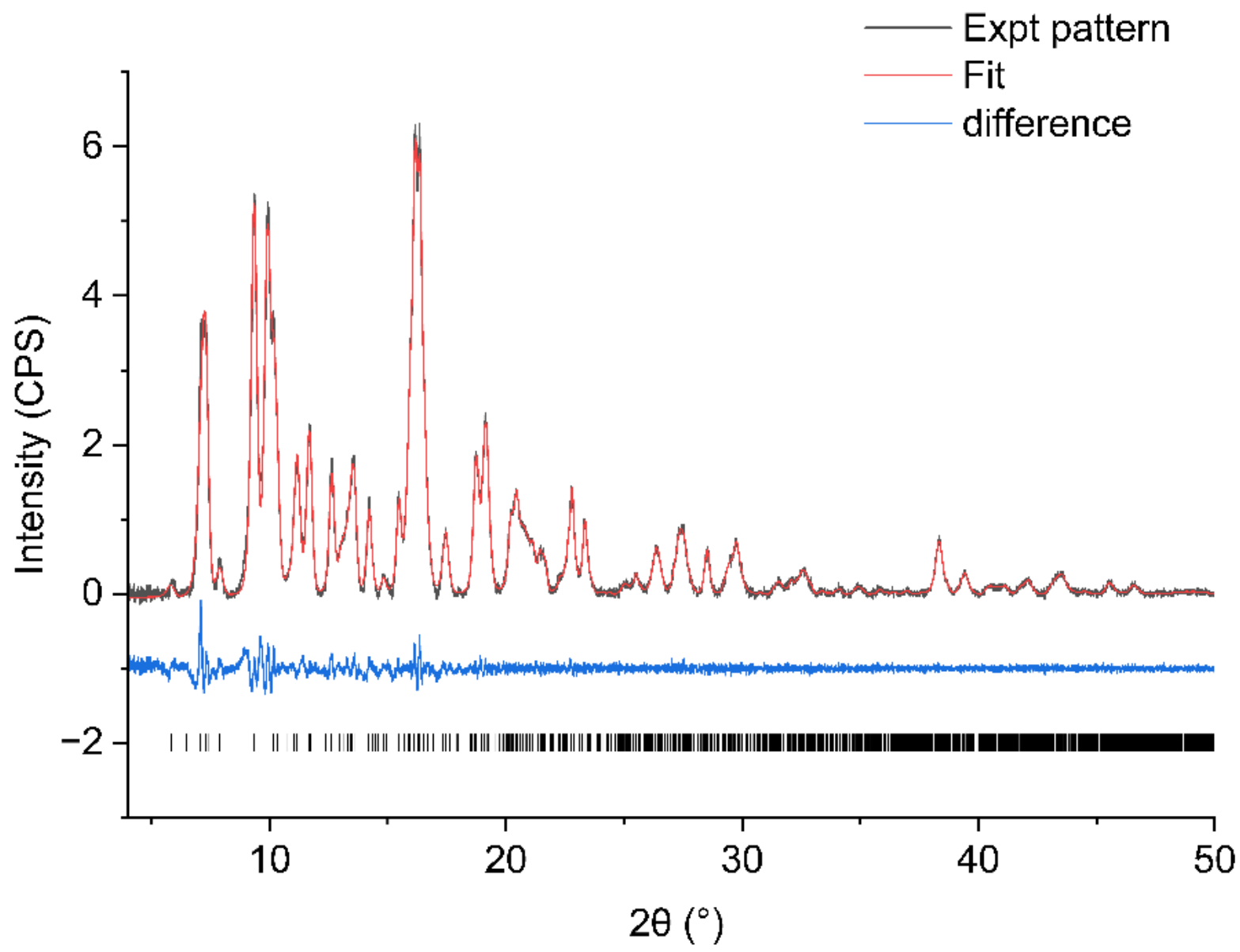

Fig. S15. Pawley fitting of the capillary PXRD data for **MeO-MOP**^{R2} showing a good match with the unit cell parameters collected from 3D ED. *Unit cell parameters: a = 13.816(9) Å; b = 14.596(10) Å; c = 16.539(11) Å; α = 100.868(34)°; β = 108.246(19)°; γ = 103.976(22)°.* R_{wp} *= 8.9%*

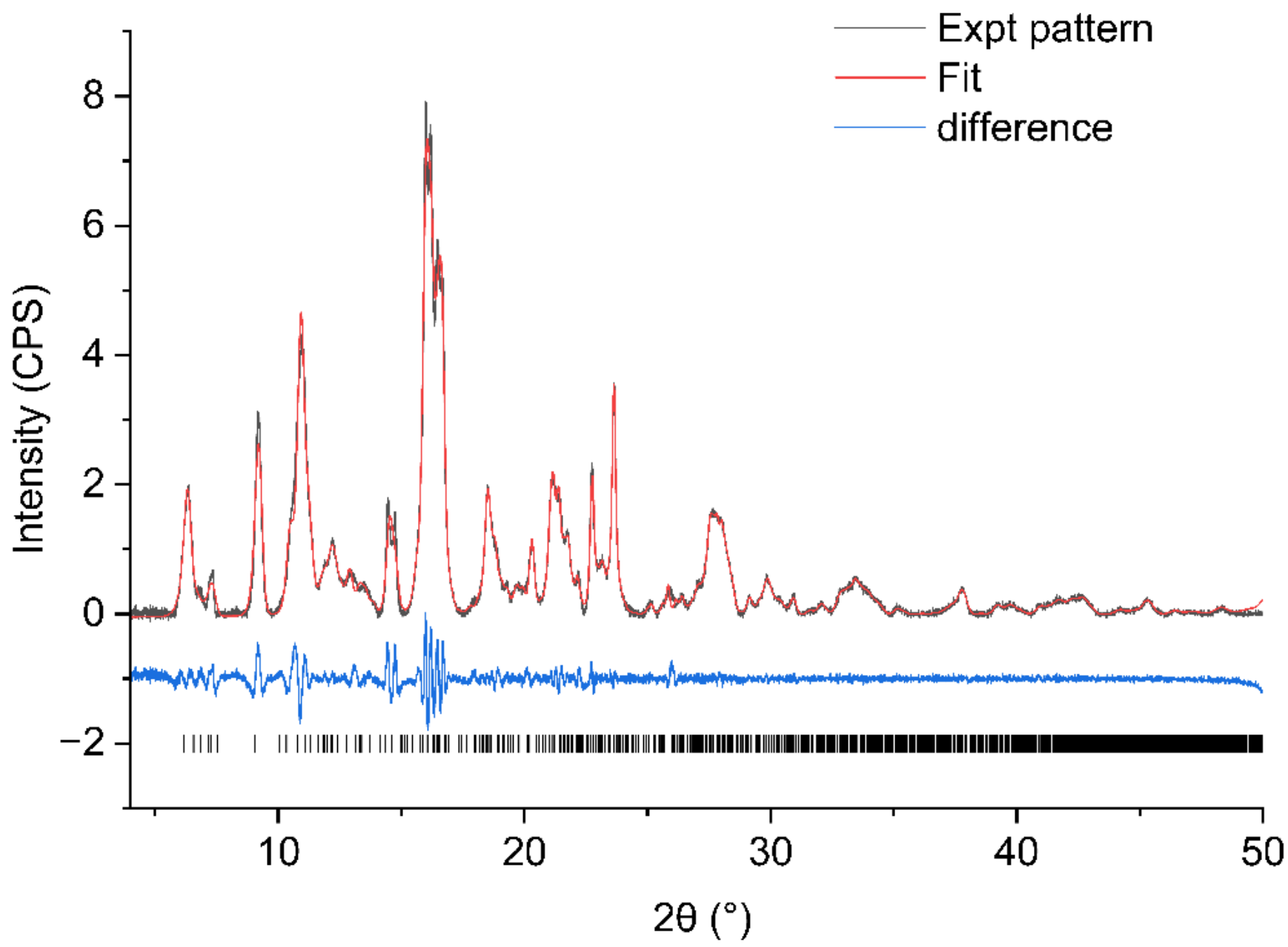

Fig. S16. Pawley fitting of the capillary PXRD data for **Br-MOP**^{R2} showing a good match with the unit cell parameters for the DMA solvated form of Br-MOP, previously reported. *Unit cell parameters: a = 15.847(13) Å; b = 14.108(7) Å; c = 16.719(8) Å; α = 100.491(33)°; β = 113.359(26)°; γ = 110.053(26)°.* R_{wp} *= 11.87%*

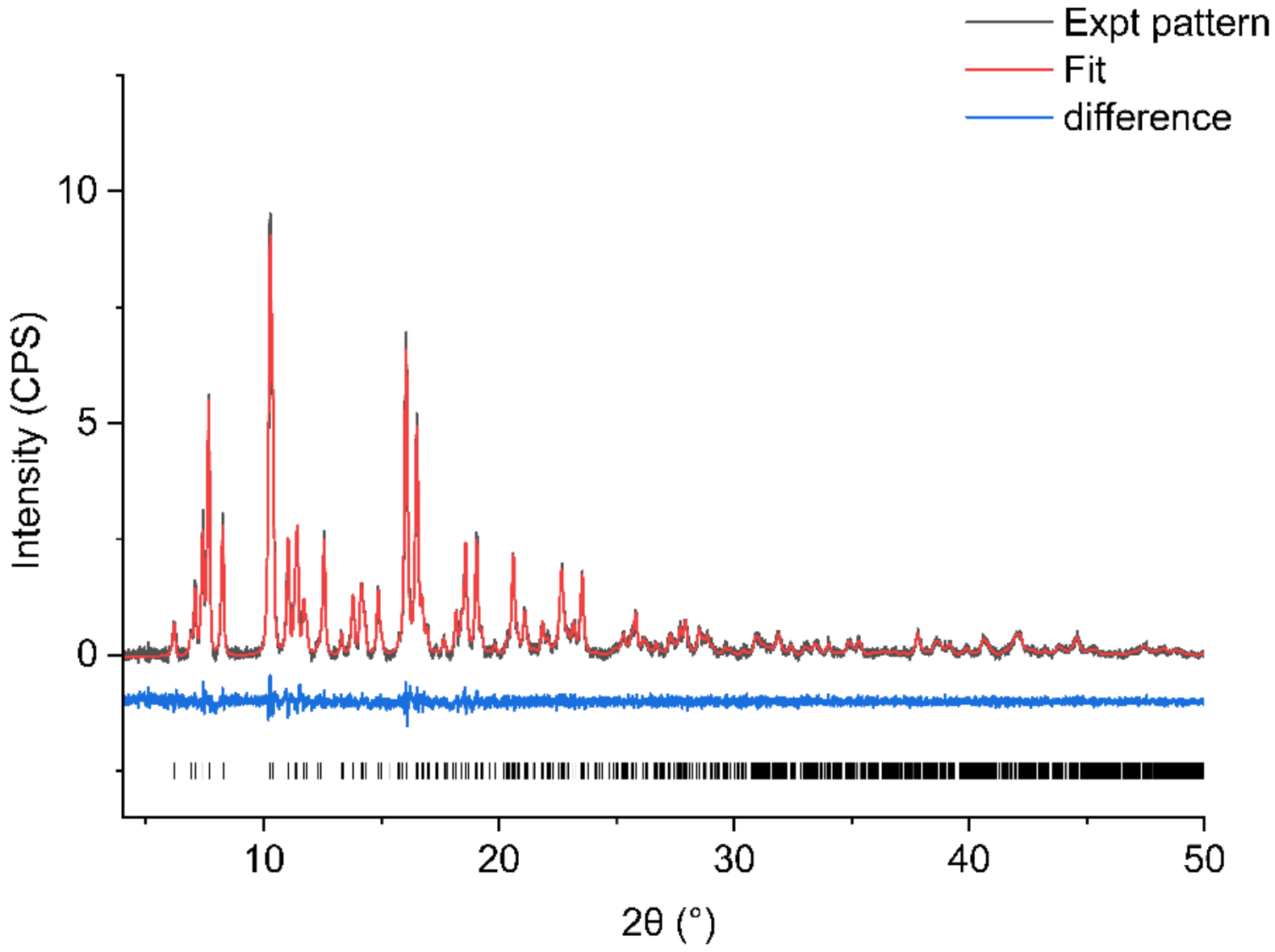

Fig. S17. Pawley fitting of the capillary PXRD diffractogram for **MOP**^{R2} showing a good match with the unit cell parameters collected from 3D ED. *Unit cell parameters: a = 13.143(43) Å; b = 14.398(48) Å; c = 16.280(53) Å; α = 109.416(6)°; β = 106.082(7)°; γ = 102.211(8)°.* R_{wp} *= 12.9%.*

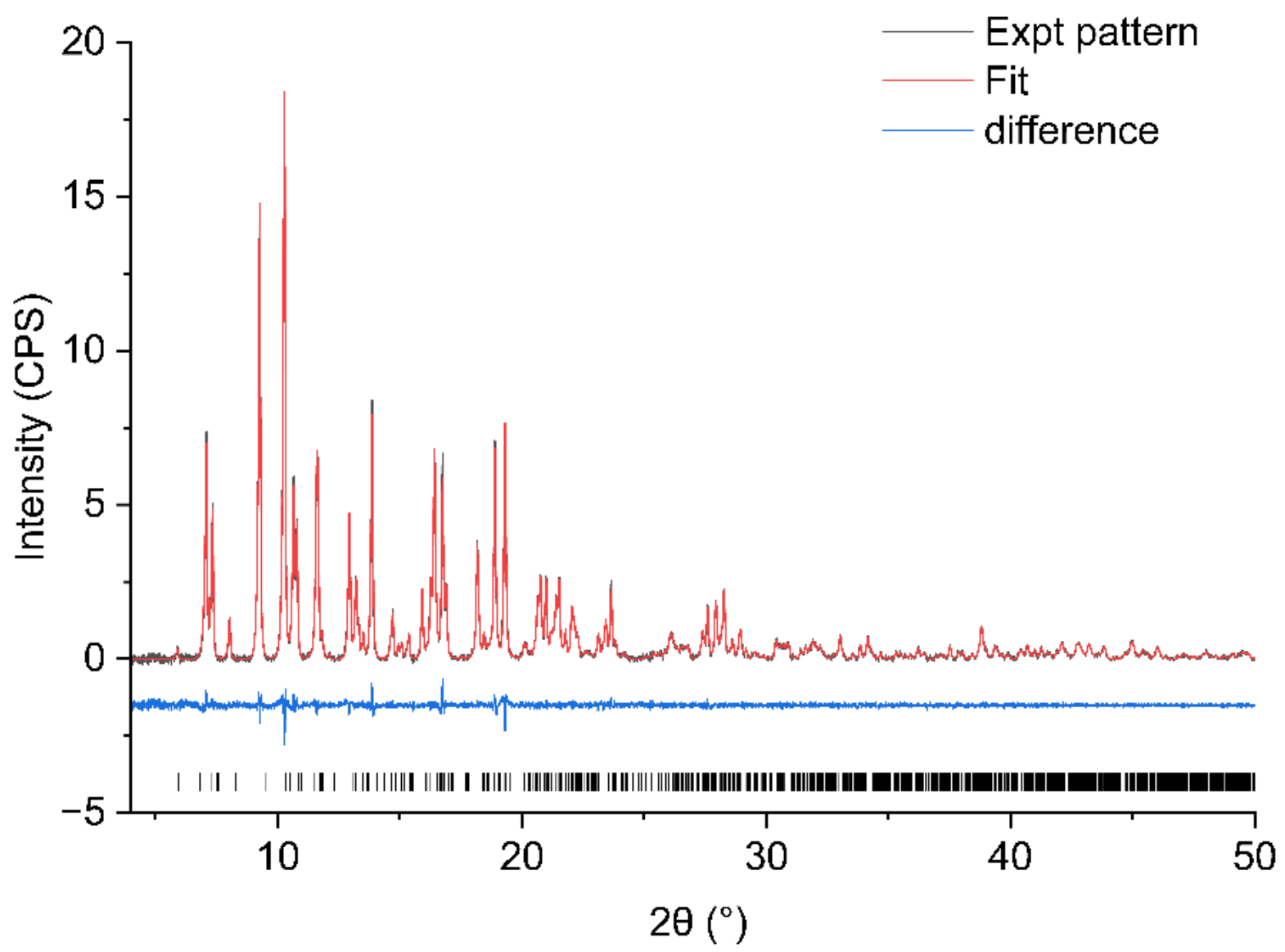

Fig. S18. Pawley fitting of the capillary PXRD diffractogram for **CH_3-MOP**^{R2} showing a good match with the unit cell parameters collected from 3D ED. *Unit cell parameters: a = 13.877(2) Å; b = 14.222(2) Å; c = 16.987(2) Å; α = 100.714(3)°; β = 112.223(3)°; γ = 104.422(3)°.* R_{wp} *= 8.5%.*

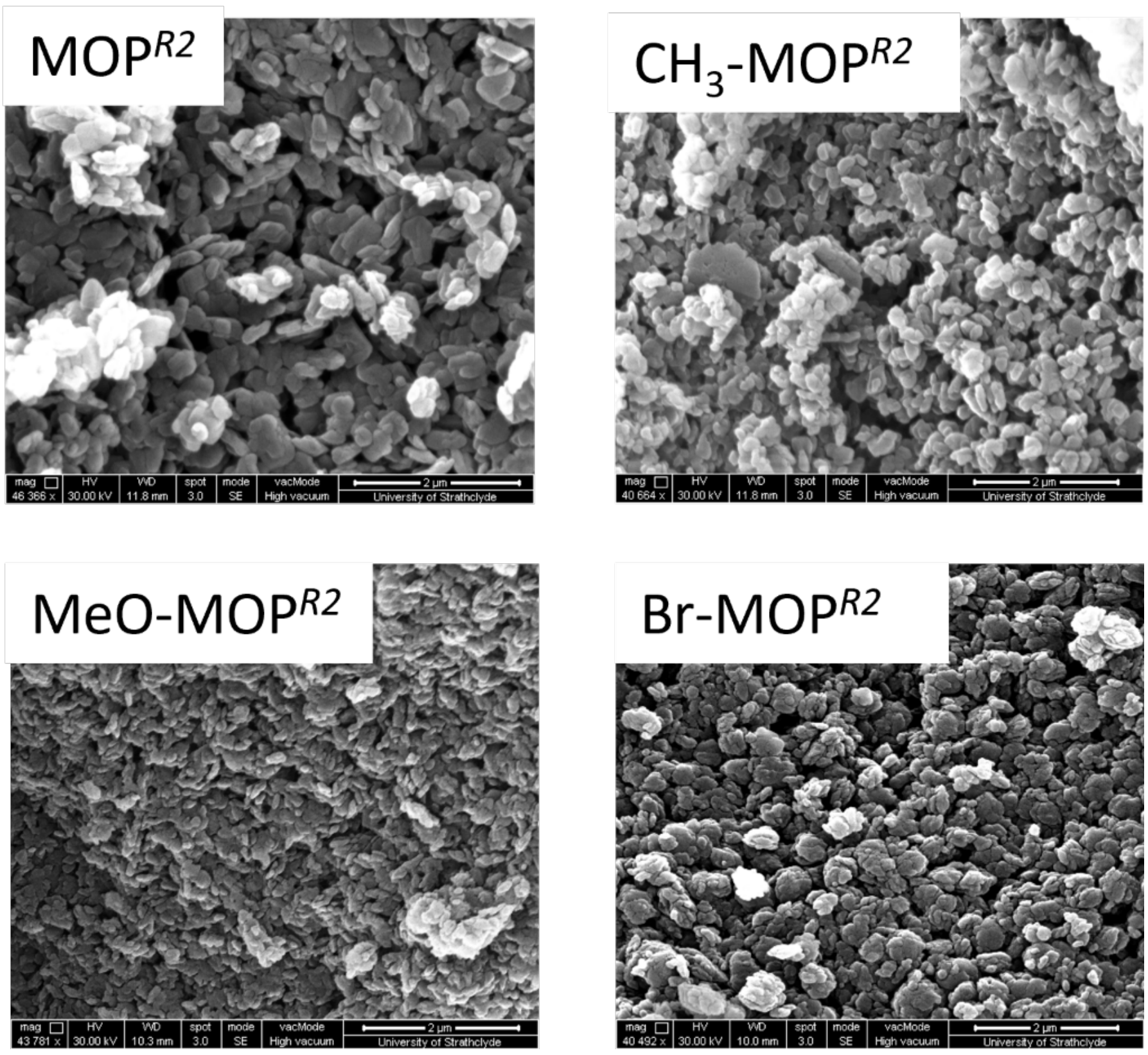

Fig. S19. SEM images of **MOP^{R2}**, **CH$_3$-MOP^{R2}**, **MeO-MOP^{R2}** and **Br-MOP^{R2}**. The scale bar is 2 μm.

Table S3. Solubility of reagents in both DMA and MeOH, calculated from NMR data. Concentration of ~0.07 mmol/mL reflective of solvothermal reaction conditions and ~0.8 mmol/mL reflective of mechanochemical slurry. Note that these slurry conditions are more dilute than those used in the reactions, to facilitate data acquisition. NMR spectra found in Figs S20, S22 – S24.

**Precursor**	**Solvent**	***η* (µL/mg)**	**Visibly soluble?**	**Theor. solvent:ligand Ratio (mol:mol)**	**Solvent NMR Integral**	**Actual solvent:ligand Ratio (mol:mol)**	**Theor. conc./ M**	**Actual conc./ M**	**Comments**
**LH_2**	DMA	29.2	Y	152.59	672	224	0.07	0.048	Fully soluble, NMR shows lower concentration than calculated due to errors in integration at this level of dilution.
**CH_3LH_2**		26.9	Y	148.98	640	213	0.07	0.050	
**MeO-LH_2**		25.9	Y	152.38	552	184	0.07	0.058	
**$BrLH_2$**		21.9	Y	151.57	497	166	0.07	0.065	
**LH_2**		2.6	Y	13.62	43	14	0.79	0.76	Fully soluble by NMR, very close to saturation.
**CH_3LH_2**		2.4	partially				0.81		Partially soluble, but solvent soaked up by solid.
**MeO-LH_2**		2.3	Y	13.61	44	15	0.79	0.740	Full soluble by NMR; yields a viscous solution that is extremely close to saturation.
**$BrLH_2$**		2.0	Y	13.53	40	13	0.80	0.799	
**LH_2**	MeOH	29.2	N	31.27	0	0	0.07	0	Insoluble.
**CH_3LH_2**		26.9	N	30.53	0	0	0.07	0	
**MeO-LH_2**		25.9	N	31.23	0	0	0.07	0	
**$BrLH_2$**		21.9	N	31.06	0	0	0.07	0	
**Copper acetate**	DMA	70.0	Y	150.31	463	154	0.07	0.07	Fully soluble.
		6.3	partially	13.42	202	67	0.80	0.160	NMR confirms partial solubility.
	MeOH	70.0	partially	345.03	2356	785	0.07	0.031	NMR confirms partial solubility.
		6.3	partially	30.81	1800	600	0.80	0.041	

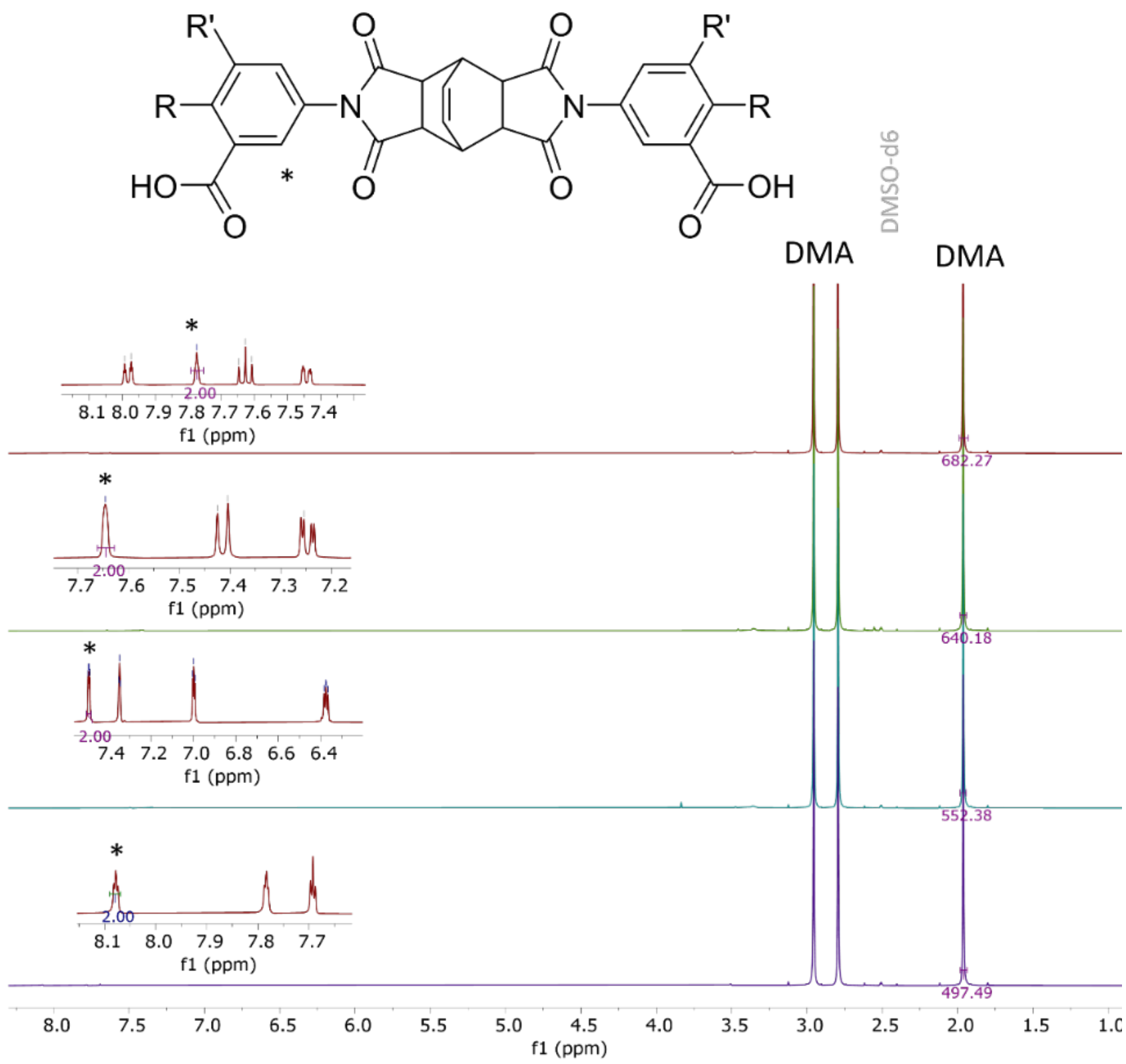

Fig. S20. NMR data showing a) LH_2, b) CH_3LH_2, c) MeO-LH_2 and d) $BrLH_2$ in DMA at solvothermal concentration (0.07 mmol/mL). The insets show a zoom of the aromatic region of the ligands. The relative ratio of solvent to ligand is calculated from the proton signal shown with an asterisk (see Table S3).

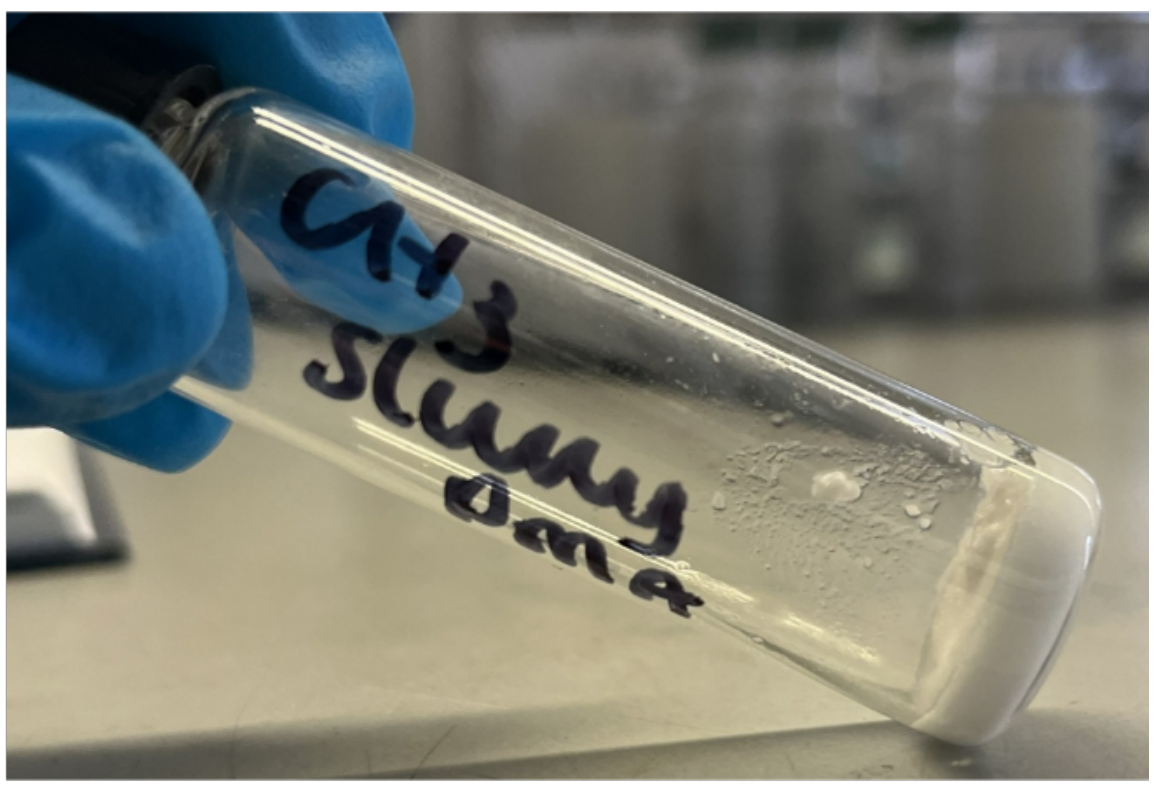

Fig. S21. CH_3LH_2 in DMA at slurry concentration.

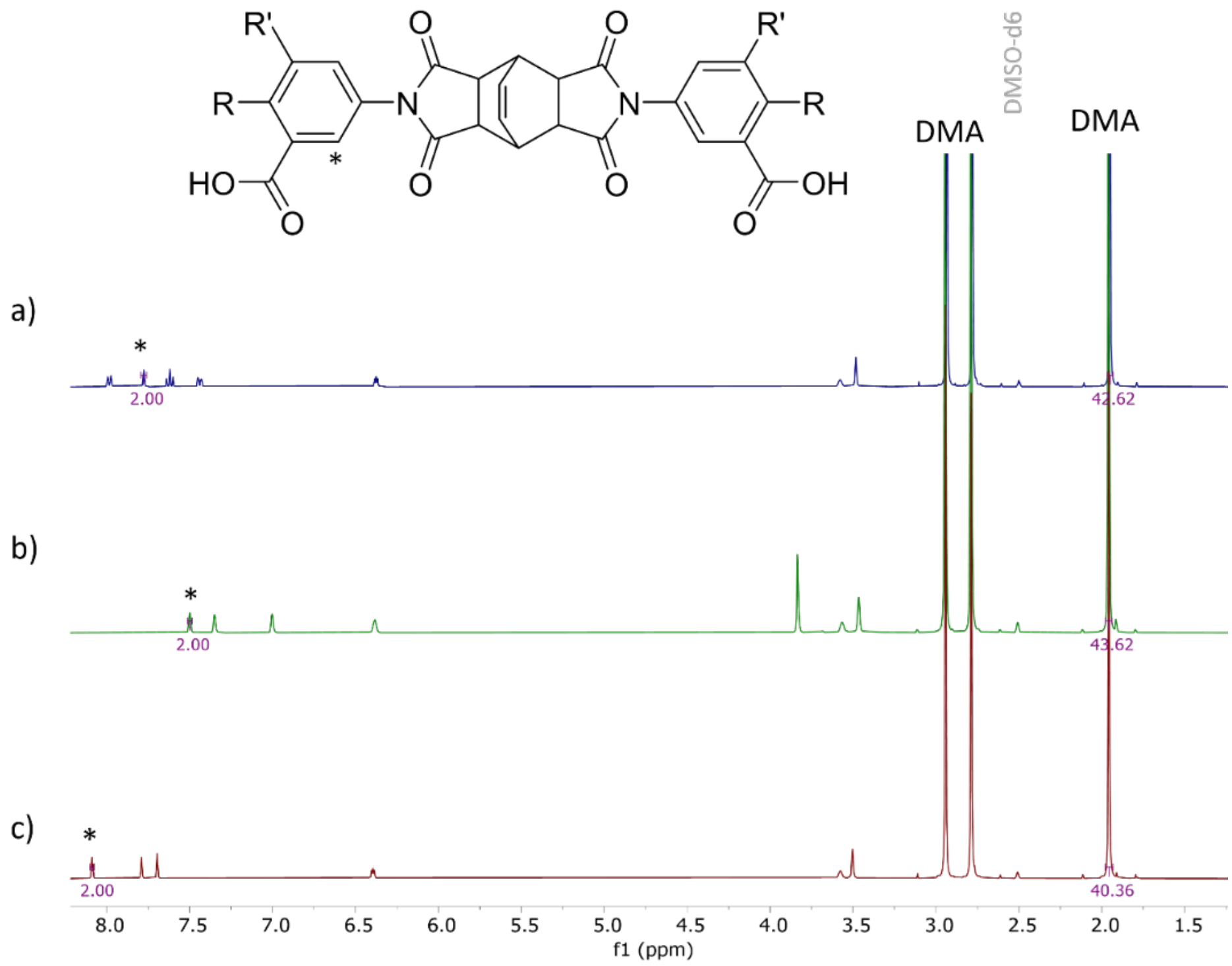

Fig. S22. NMR data showing a) MeO-LH_2 and b) $BrLH_2$ in DMA at slurry concentration (0.8 mmol/mL). The relative ratio of solvent to ligand is calculated from the proton signal shown with an asterisk (see Table S3).

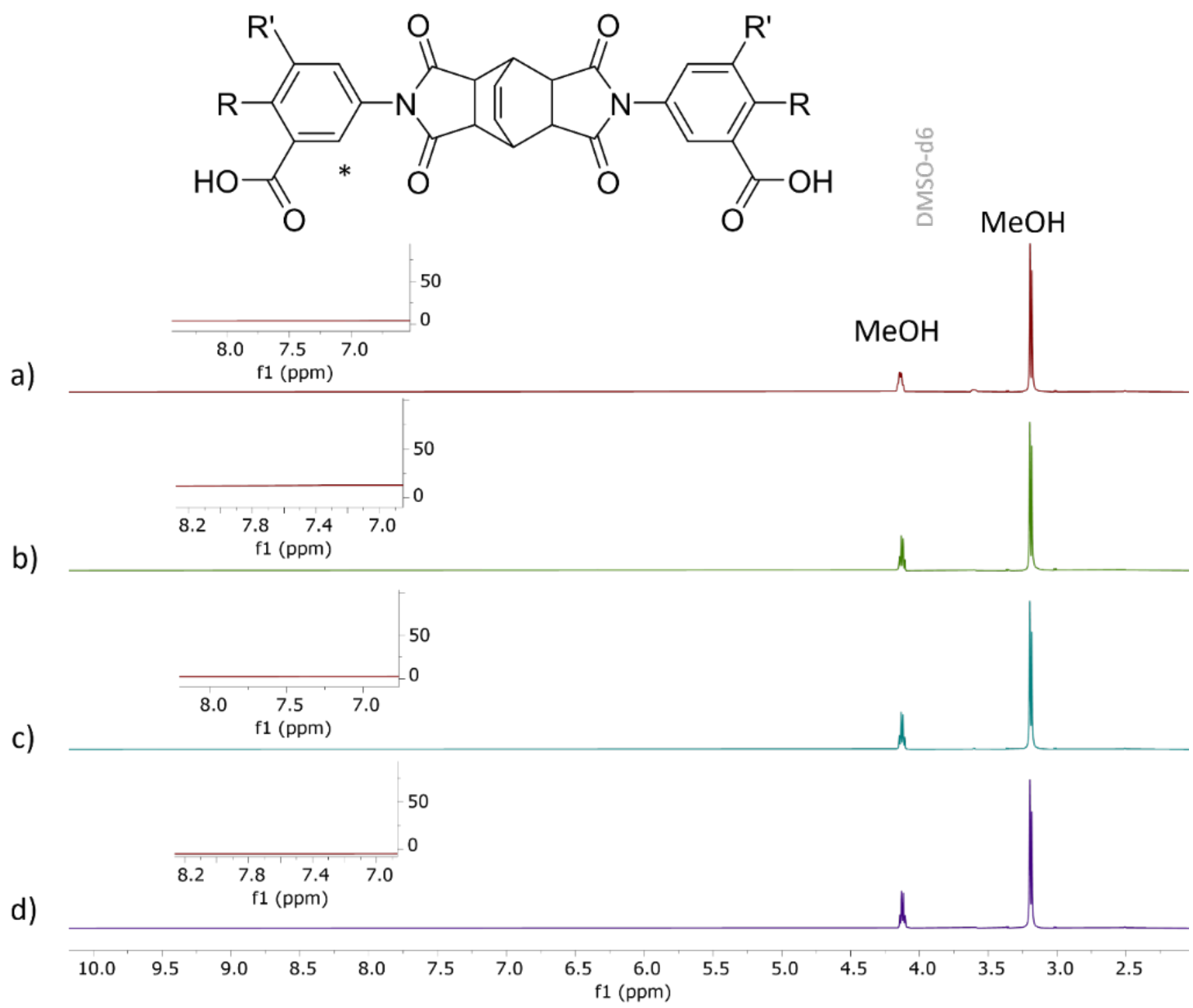

Fig. S23. NMR data showing that a) LH_2, b) CH_3LH_2, c) MeO-LH_2 and d) $BrLH_2$ are insoluble in MeOH, even at solvothermal concentration (0.07 mmol/mL).

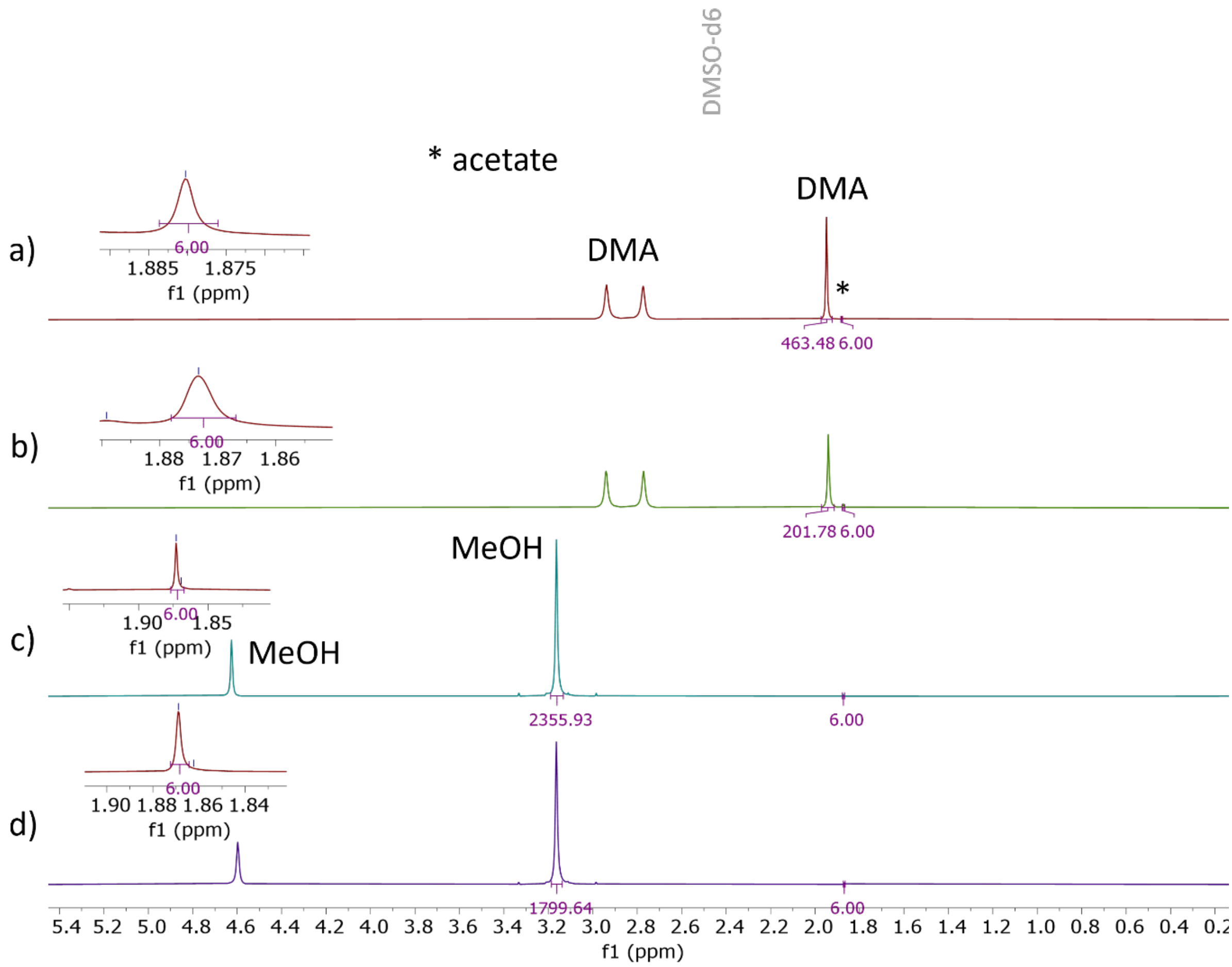

Fig. S24. NMR data showing copper acetate monohydrate in DMA at a) solvothermal concentration (0.07 mmol/mL), b) slurry concentration (0.8 mmol/mL), and in MeOH at c) solvothermal concentration (0.07 mmol/mL) and d) slurry concentration (0.8 mmol/mL).

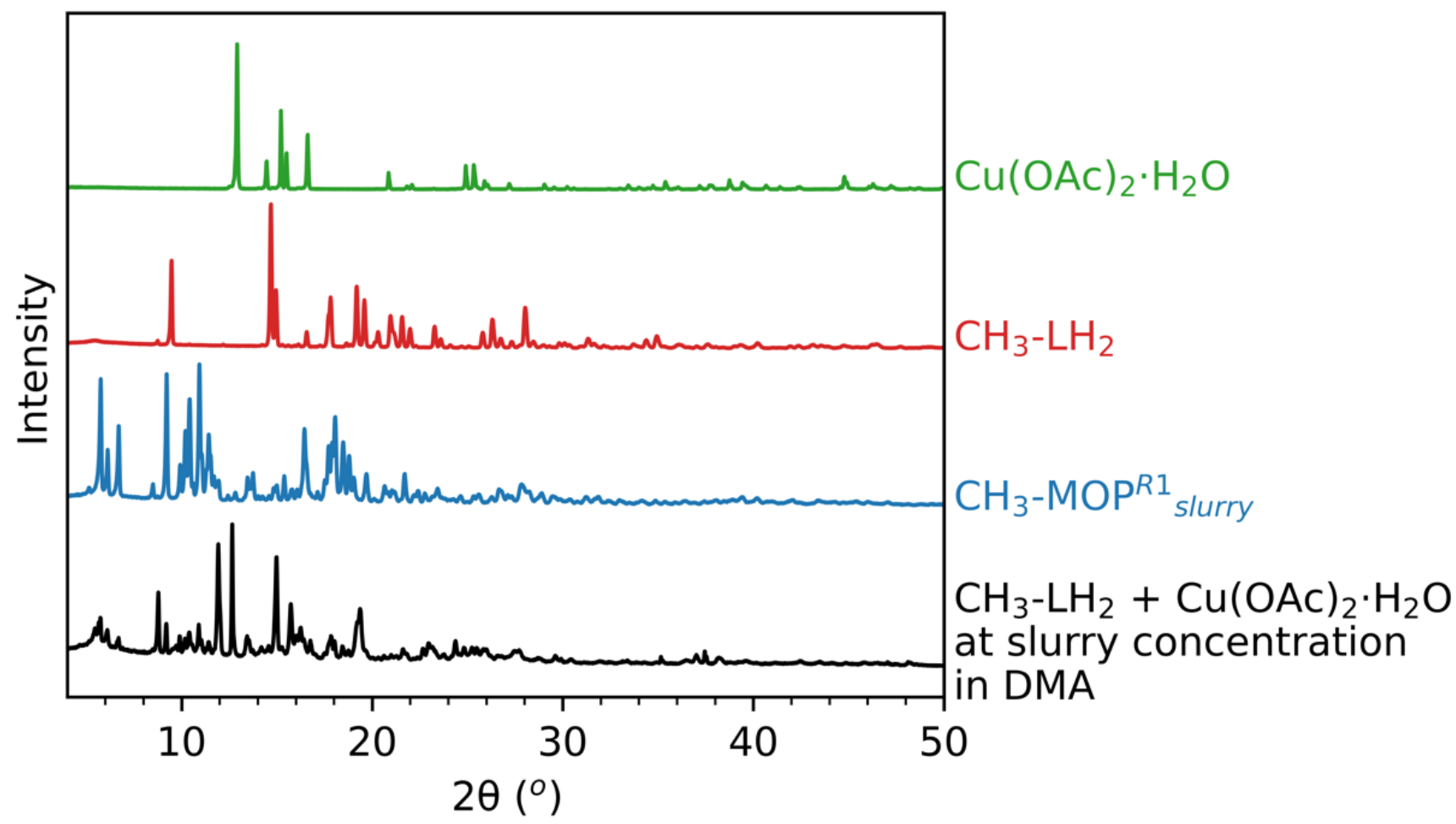

Fig. S25. Comparison of PXRD data obtained for copper acetate, the ligand CH_3LH_2, the Route 1 mechanochemical synthesis of **CH_3-MOP$^{R1}_{slurry}$**, and the solid resulting from mixture of the ligand and salt in slurry conditions in the absence of mechanochemistry.

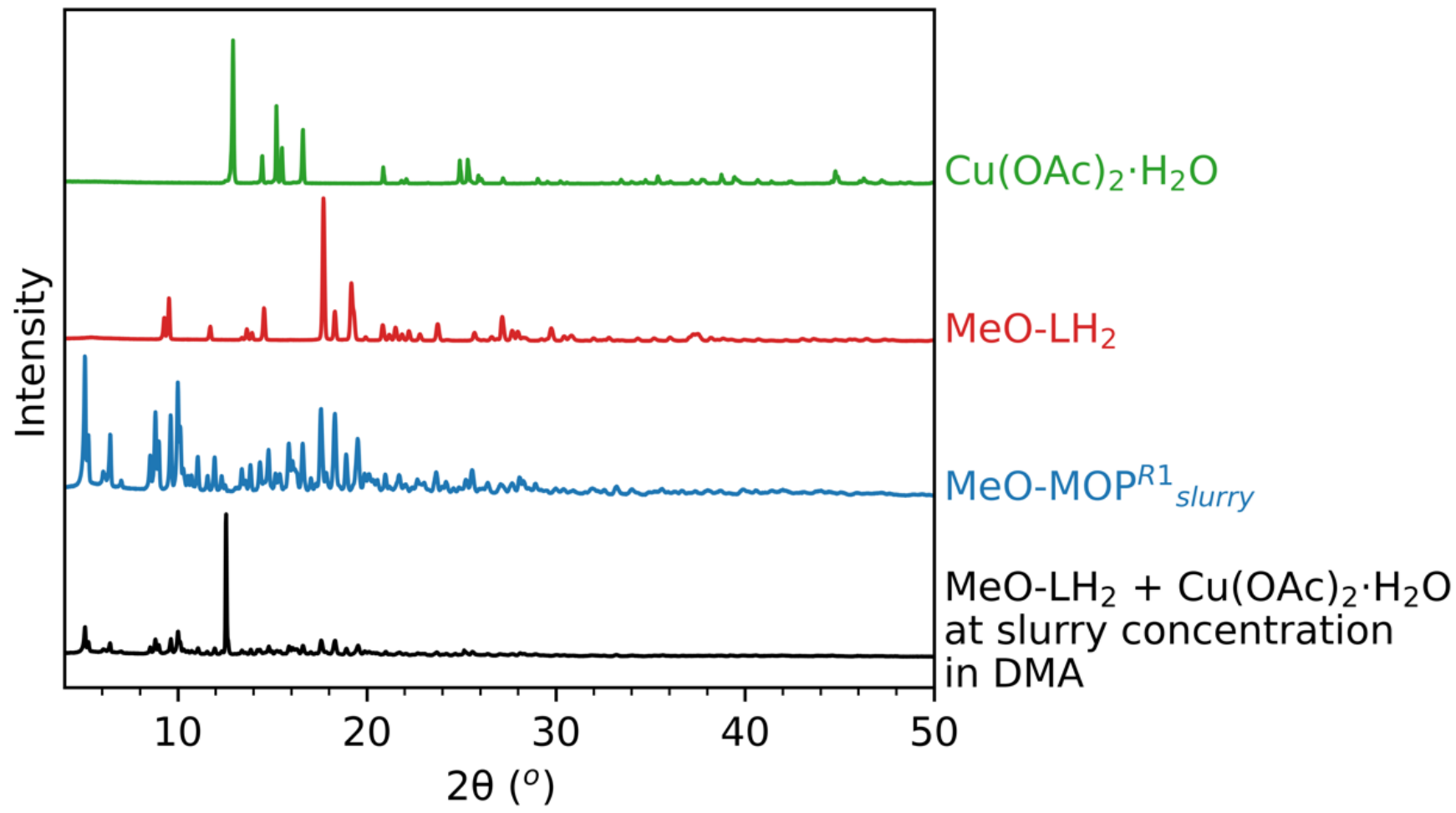

Fig. S26. Comparison of PXRD data obtained for copper acetate, the ligand MeO-LH_2, the Route 1 mechanochemical synthesis of **MeO-MOP^{R1}$_{slurry}$**, and the solid resulting from mixture of the ligand and salt in slurry conditions in the absence of mechanochemistry.

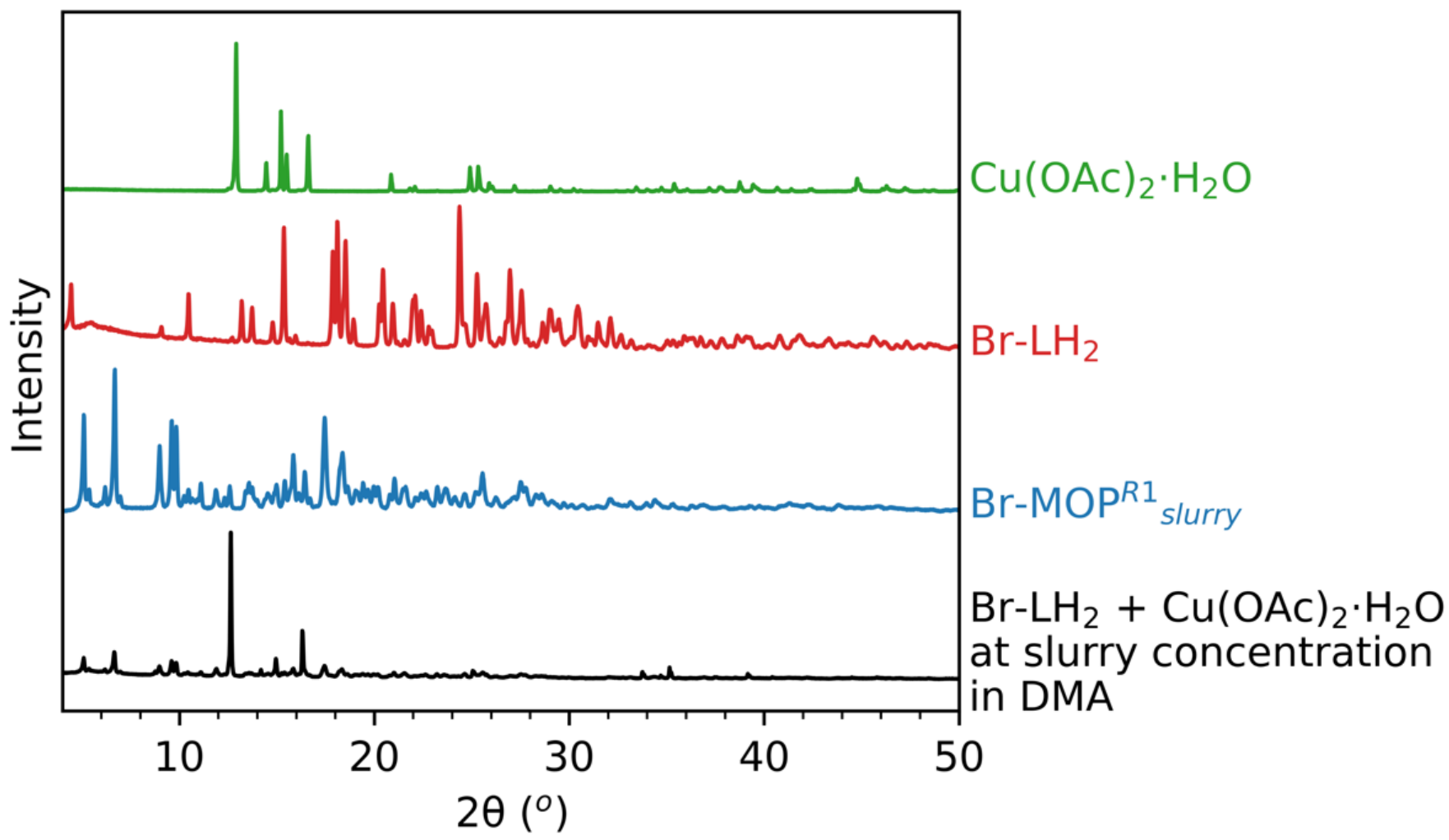

Fig. S27. Comparison of PXRD data obtained for copper acetate, the ligand BrLH_2, the Route 1 mechanochemical synthesis of **Br-MOP^{R1}$_{slurry}$**, and the solid resulting from mixture of the ligand and salt in slurry conditions in the absence of mechanochemistry.

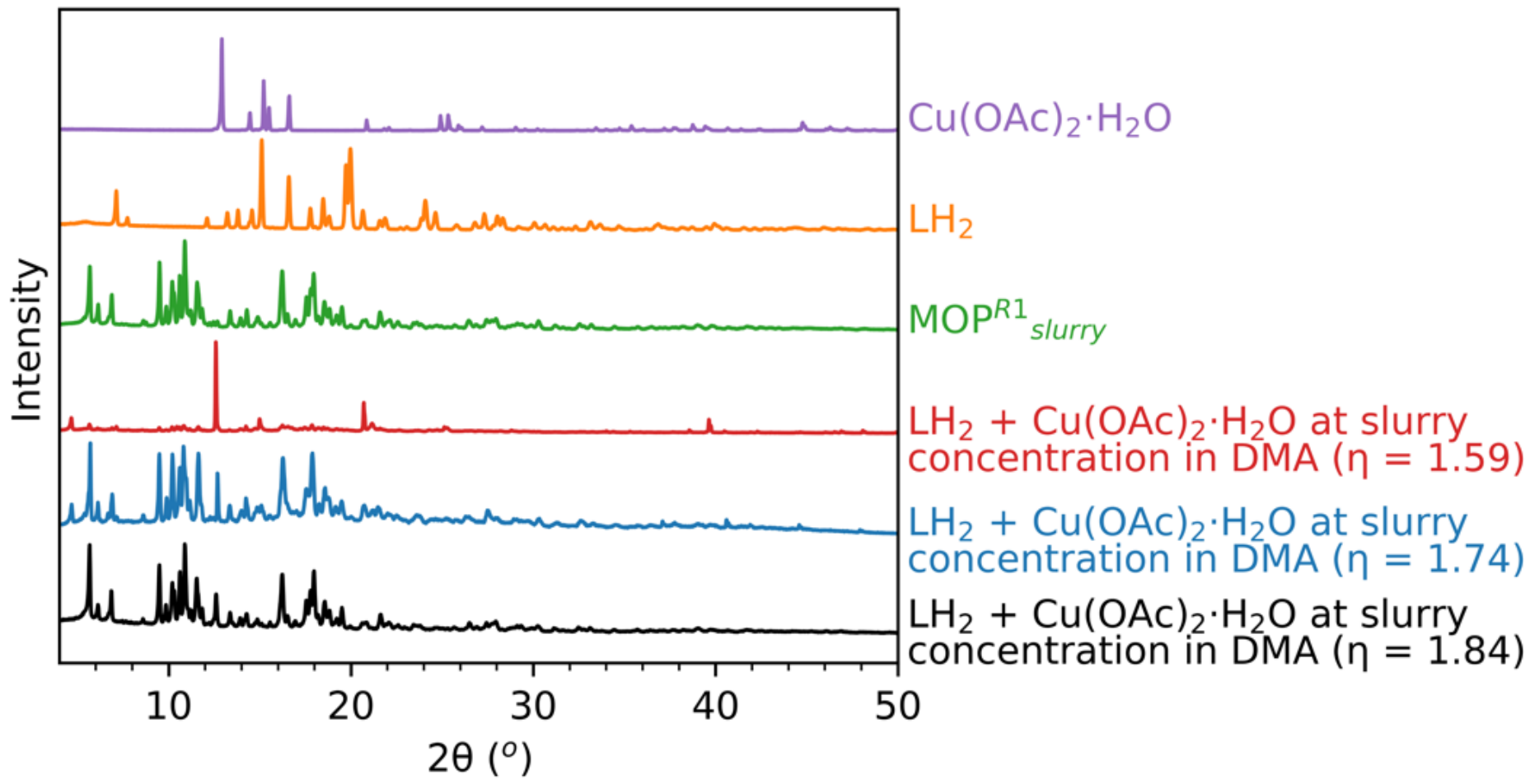

Fig. S28. Comparison of PXRD data obtained for copper acetate, the ligand LH_2, the Route 1 mechanochemical synthesis of **$MOP^{R1}{}_{slurry}$**, and the solid resulting from mixture of the ligand and salt in slurry conditions in the absence of mechanochemistry at varying values of *η*.

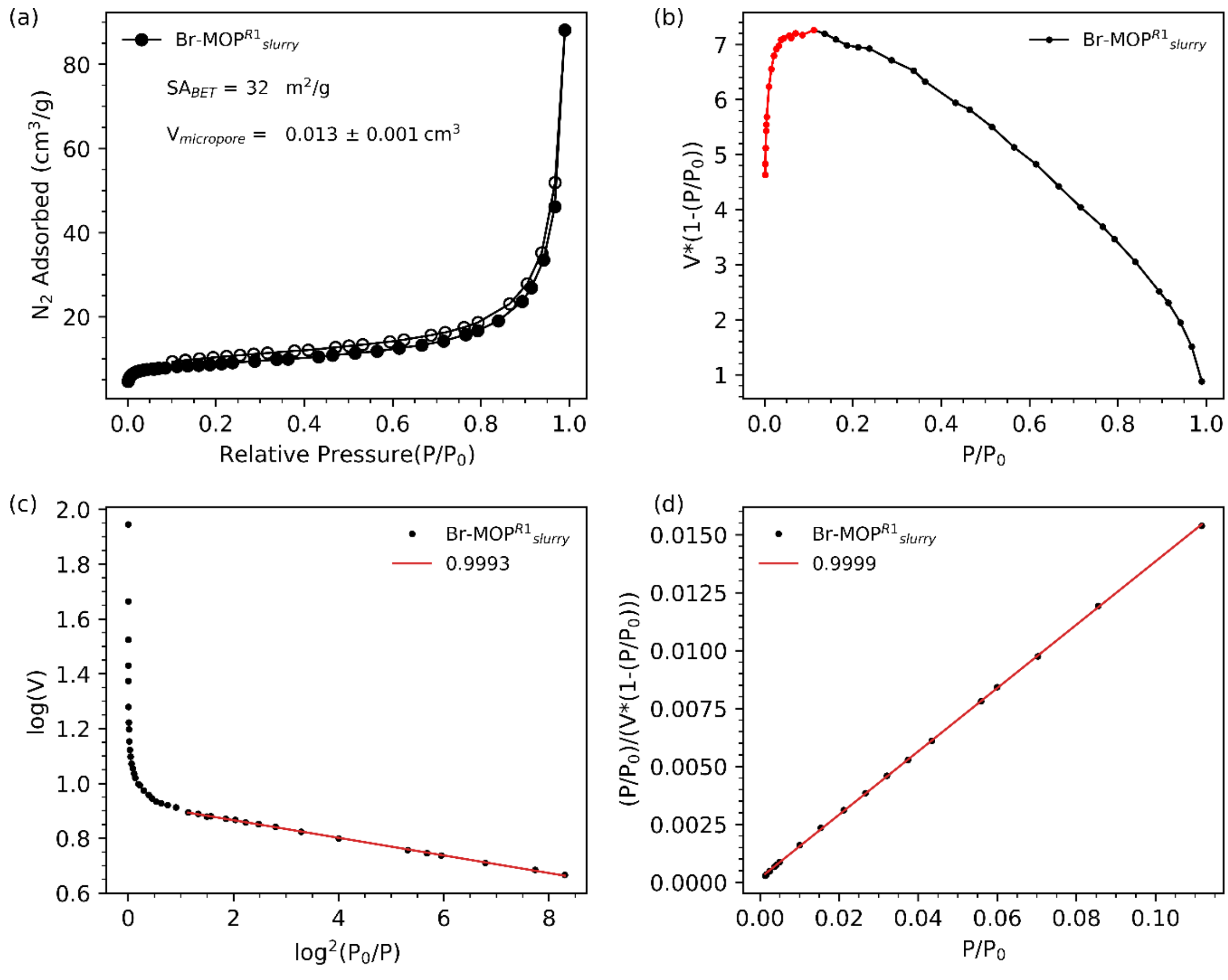

Fig. S29. a) N_2 isotherm of **Br-MOP*^{R1}***, measured at −196 °C, showing adsorption in filled circles and desorption in open circles, with the red point being the plateau value of the uptake. b) is the Rouquerol factor plotted against the relative pressure, where the points in red are those included in the linearisation to find the BET surface area. c) Dubinin-Radushkevich plot and linear region used to calculate the micropore volume in the sample. d) The linear regression plot from which the BET surface area is calculated.

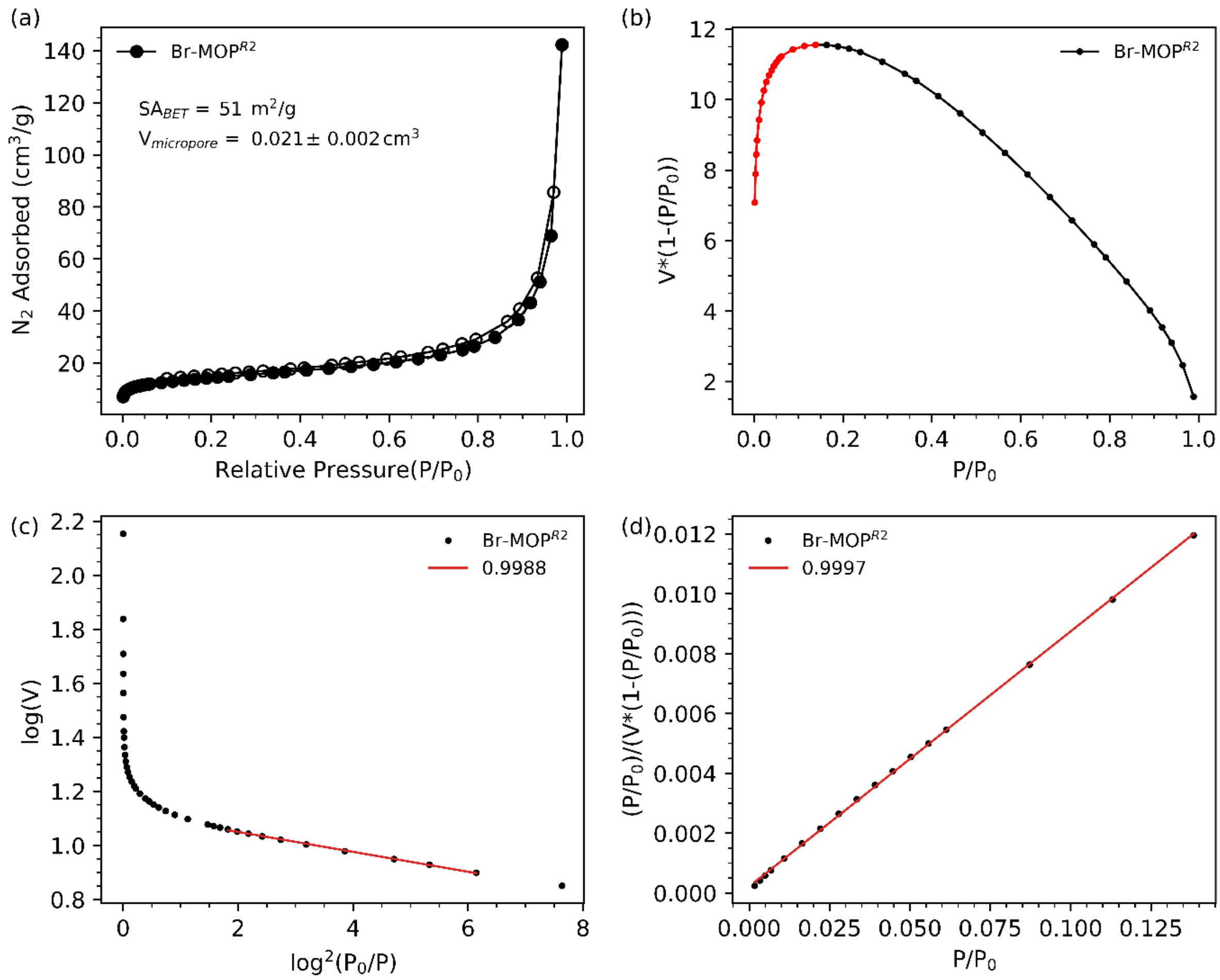

Fig. S30. a) N_2 isotherm of **Br-MOP^{R2}**, measured at −196 °C, showing adsorption in filled circles and desorption in open circles, with the red point being the plateau value of the uptake. b) is the Rouquerol factor plotted against the relative pressure, where the points in red are those included in the linearisation to find the BET surface area. c) Dubinin-Radushkevich plot and linear region used to calculate the micropore volume in the sample. d) The linear regression plot from which the BET surface area is calculated.

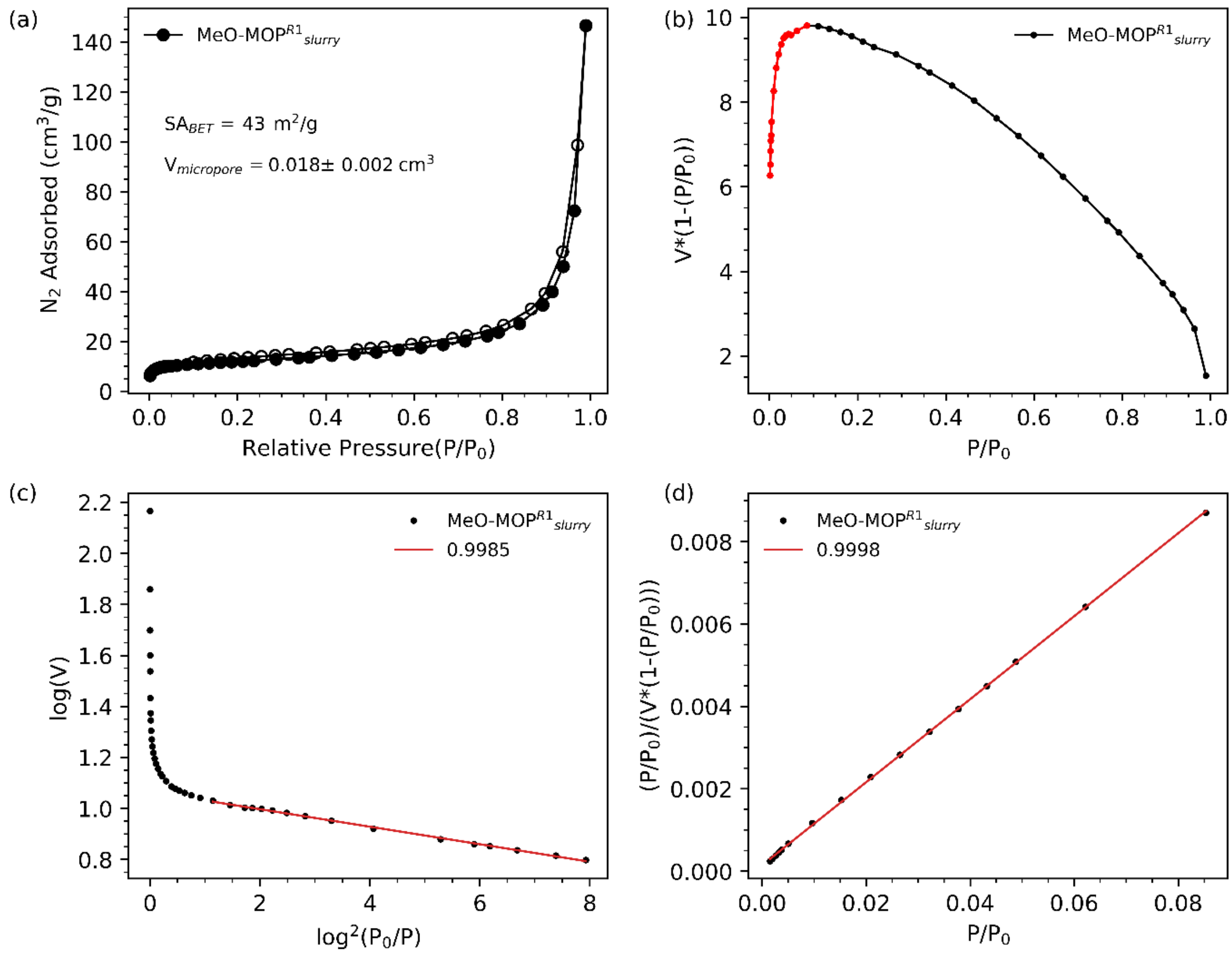

Fig. S31. a) N_2 isotherm of **MeO-MOP^{R1}**, measured at −196 °C, showing adsorption in filled circles and desorption in open circles, with the red point being the plateau value of the uptake. b) is the Rouquerol factor plotted against the relative pressure, where the points in red are those included in the linearisation to find the BET surface area. c) Dubinin-Radushkevich plot and linear region used to calculate the micropore volume in the sample. d) The linear regression plot from which the BET surface area is calculated.

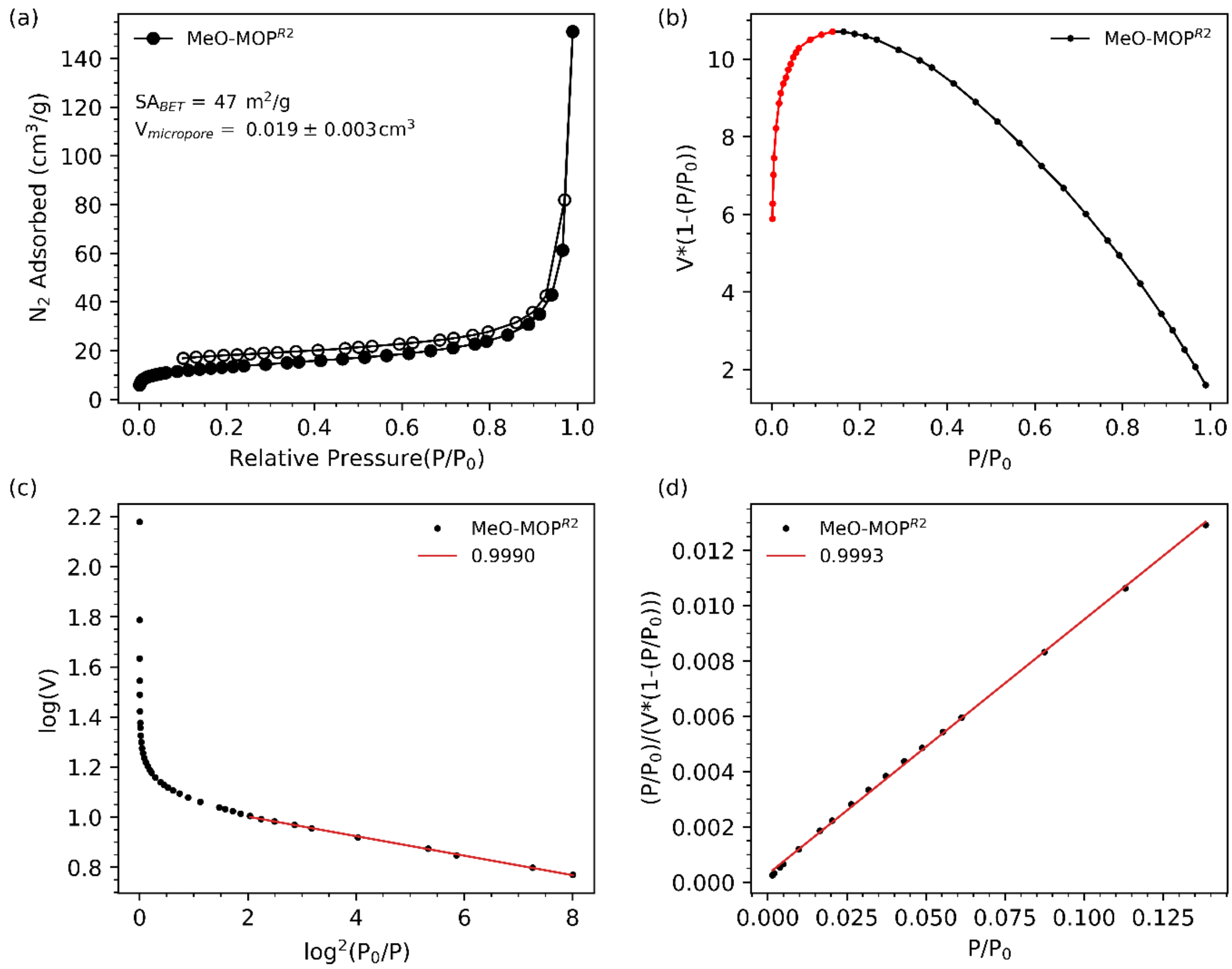

Fig. S32. a) N_2 isotherm of **MeO-MOP^{R2}**, measured at −196 °C, showing adsorption in filled circles and desorption in open circles, with the red point being the plateau value of the uptake. b) is the Rouquerol factor plotted against the relative pressure, where the points in red are those included in the linearisation to find the BET surface area. c) Dubinin-Radushkevich plot and linear region used to calculate the micropore volume in the sample. d) The linear regression plot from which the BET surface area is calculated.

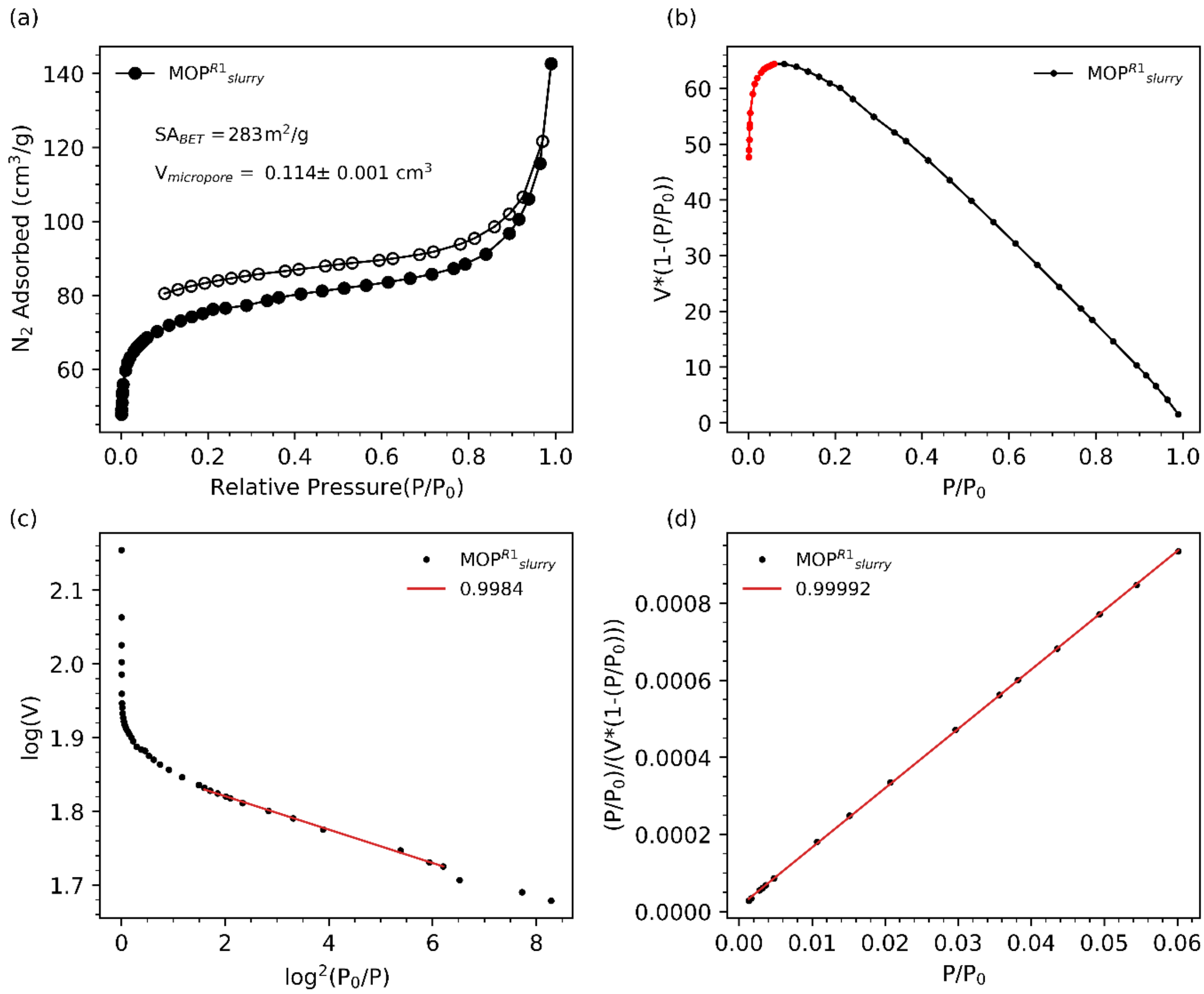

Fig. S33. a) N_2 isotherm of **MOP^{R1}**, measured at −196 °C, showing adsorption in filled circles and desorption in open circles, with the red point being the plateau value of the uptake. b) is the Rouquerol factor plotted against the relative pressure, where the points in red are those included in the linearisation to find the BET surface area. c) Dubinin-Radushkevich plot and linear region used to calculate the micropore volume in the sample. d) The linear regression plot from which the BET surface area is calculated.

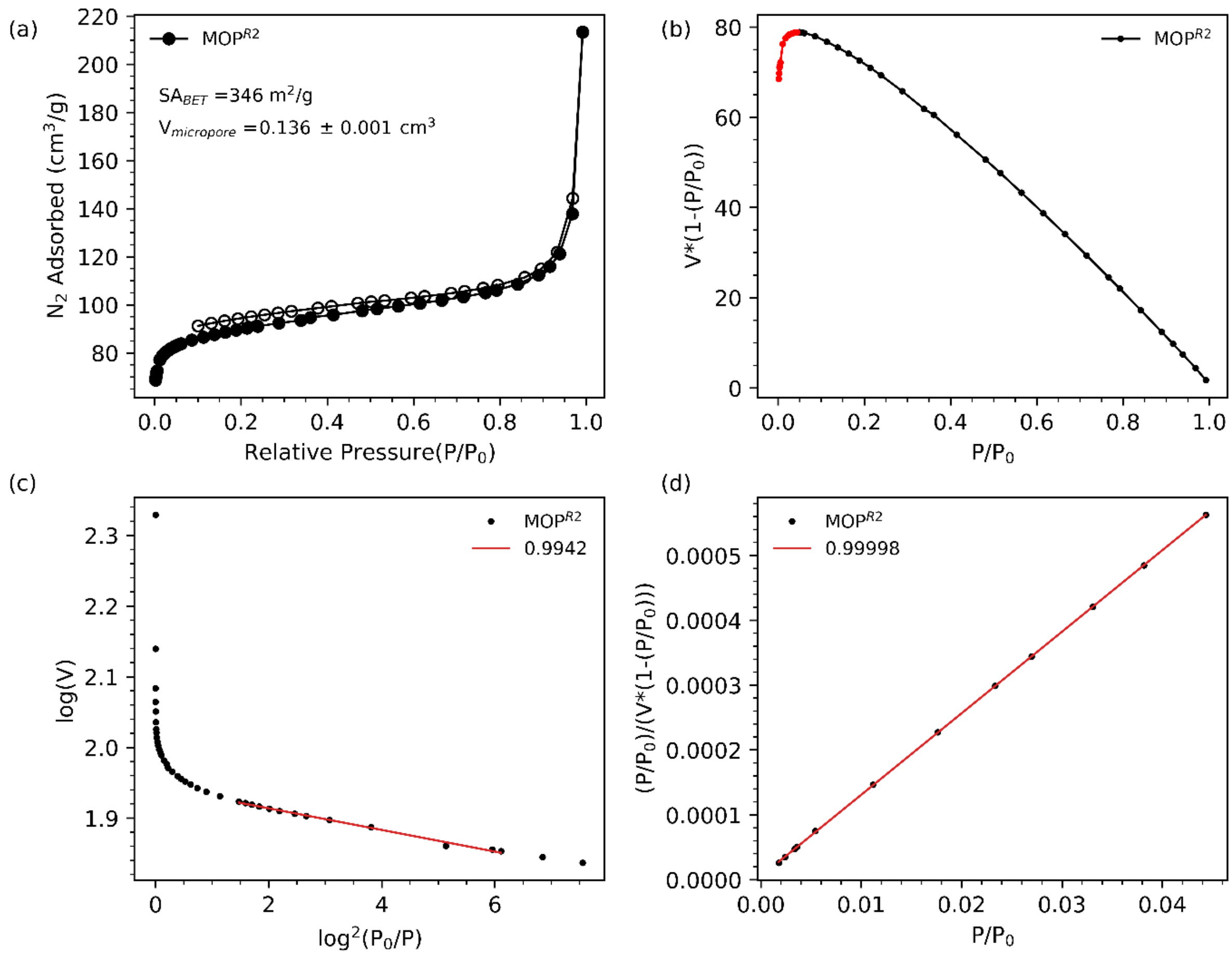

Fig. S34. a) N_2 isotherm of **MOP^{R2}**, measured at −196 °C, showing adsorption in filled circles and desorption in open circles, with the red point being the plateau value of the uptake. b) is the Rouquerol factor plotted against the relative pressure, where the points in red are those included in the linearisation to find the BET surface area. c) Dubinin-Radushkevich plot and linear region used to calculate the micropore volume in the sample. d) The linear regression plot from which the BET surface area is calculated.

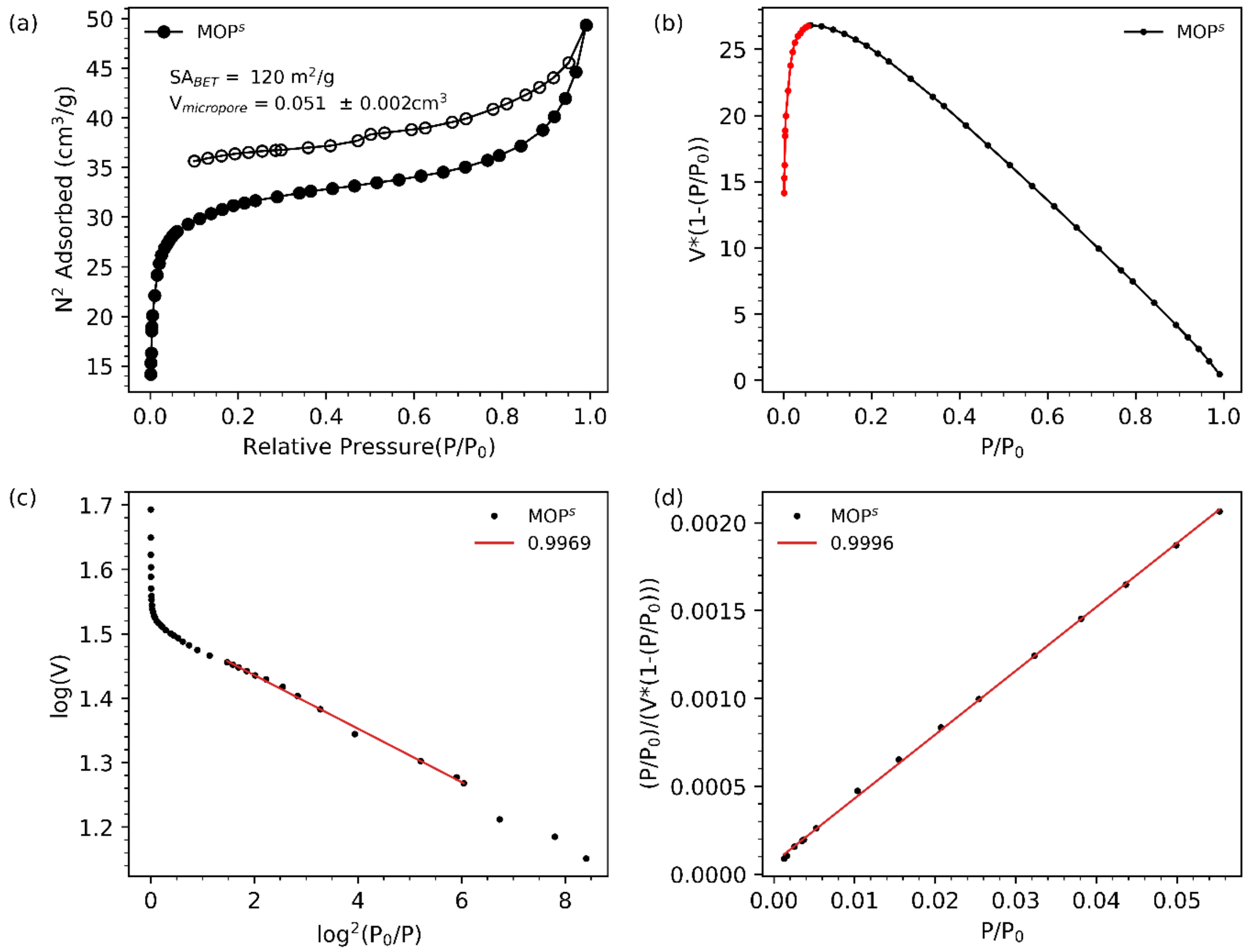

.

Fig. S35. a) N_2 isotherm of **MOP^S**, measured at −196 °C, showing adsorption in filled circles and desorption in open circles, with the red point being the plateau value of the uptake. b) is the Rouquerol factor plotted against the relative pressure, where the points in red are those included in the linearisation to find the BET surface area. c) Dubinin-Radushkevich plot and linear region used to calculate the micropore volume in the sample. d) The linear regression plot from which the BET surface area is calculated.

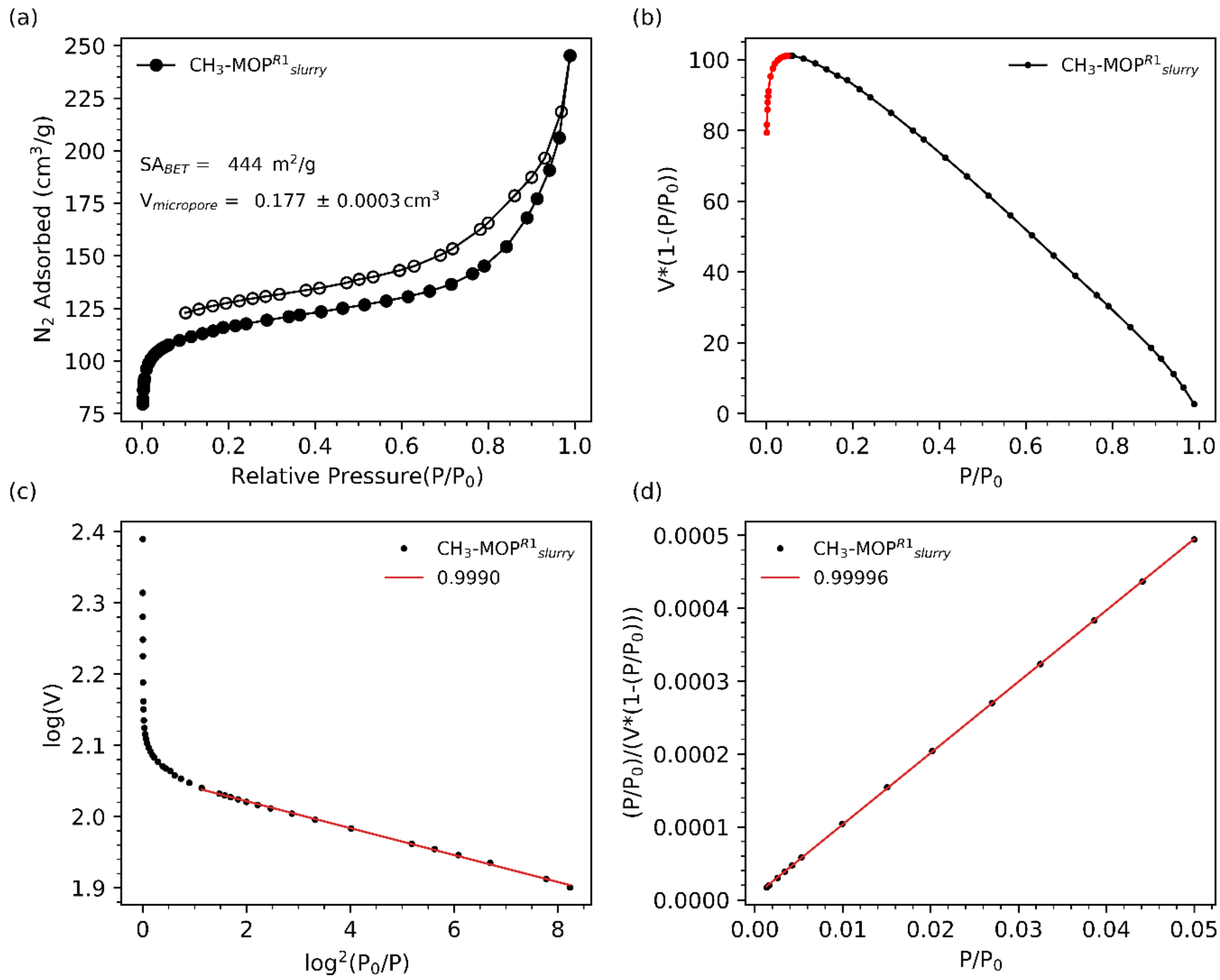

Fig. S36. a) N_2 isotherm of **CH_3-MOP^{R1}**, measured at −196 °C, showing adsorption in filled circles and desorption in open circles, with the red point being the plateau value of the uptake. b) is the Rouquerol factor plotted against the relative pressure, where the points in red are those included in the linearisation to find the BET surface area. c) Dubinin-Radushkevich plot and linear region used to calculate the micropore volume in the sample. d) The linear regression plot from which the BET surface area is calculated.

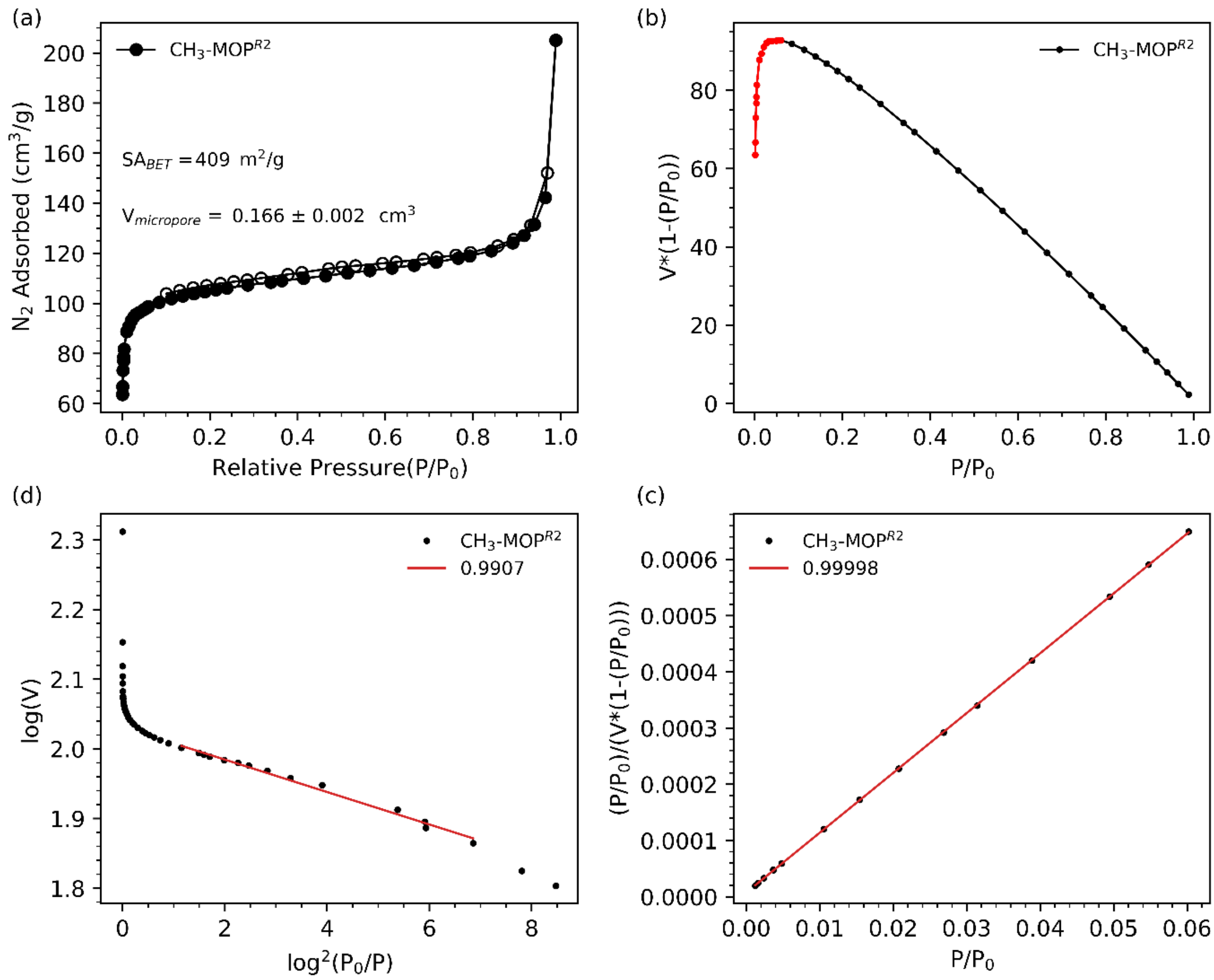

Fig. S37. a) N_2 isotherm of **CH_3-MOP*[R2]***, measured at −196 °C, showing adsorption in filled circles and desorption in open circles, with the red point being the plateau value of the uptake. b) is the Rouquerol factor plotted against the relative pressure, where the points in red are those included in the linearisation to find the BET surface area. c) Dubinin-Radushkevich plot and linear region used to calculate the micropore volume in the sample. d) The linear regression plot from which the BET surface area is calculated.

Table S4. Analysis of pore volumes derived from Dubinin-Radushkevich analysis.

Sample	V micropores (DR) (cm^3/g)	Uptake @ 0.8 *P*/*P*$_0$ (cm^3/g)	V micro+mesopores (cm^3/g)	% micropores
**MOP$^{R1}_{slurry}$**	0.114	88.40	0.136	83
**MOP^{R2}**	0.136	106.05	0.164	83
**MOP^{S}**	0.051	36.19	0.055	91
**CH_3-MOP$^{R1}_{slurry}$**	0.177	145.12	0.224	79
**CH_3-MOP^{R2}**	0.166	118.91	0.183	90
**CH_3-MOP^{S}**	0.209	162.08	0.250	83

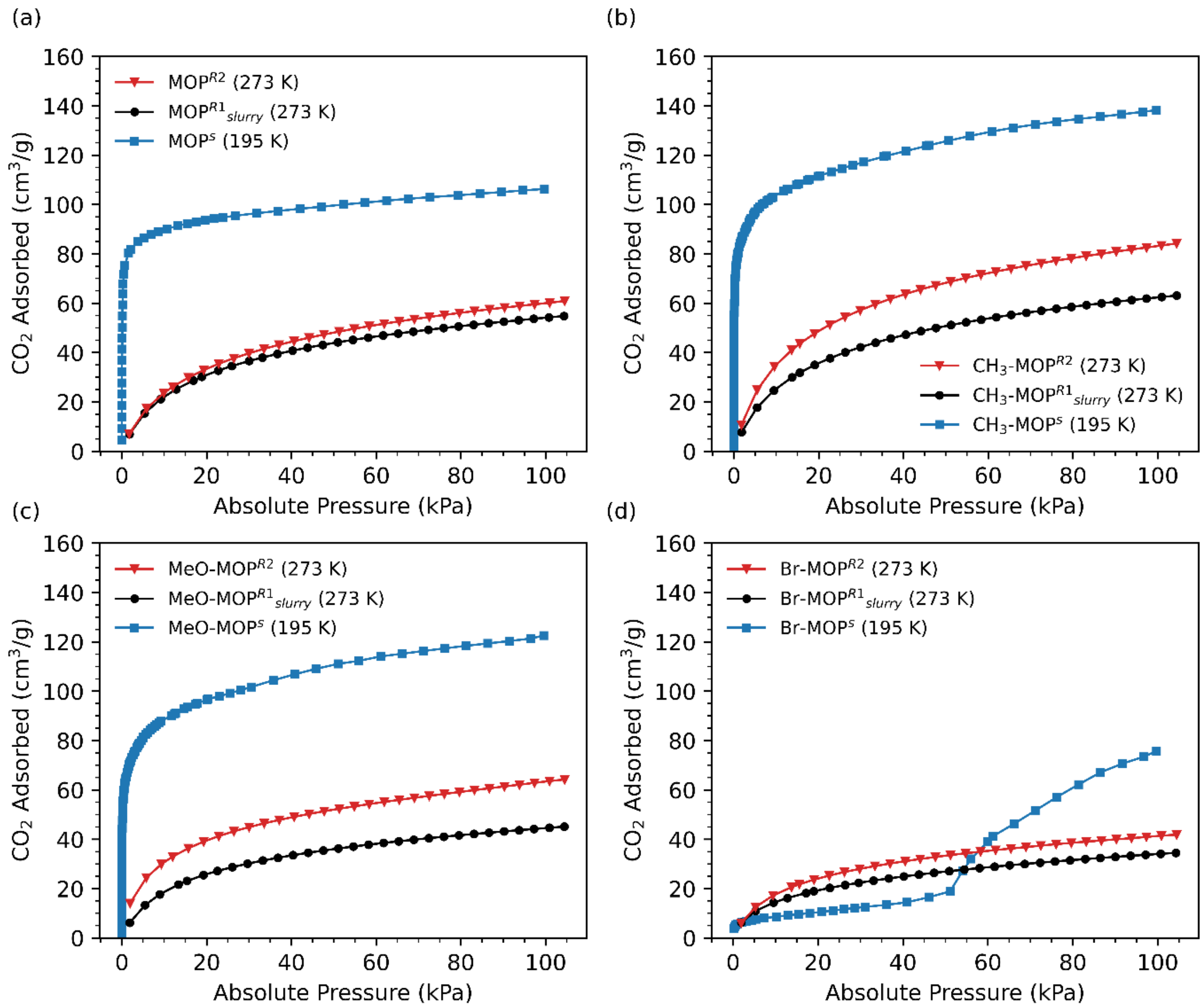

Fig. S38. CO_2 isotherms for $R1_{slurry}$ and R2 cages compared to previously published data collected at −78 °C, for a) **MOP**, b) **CH_3-MOP**, c) **MeO-MOP** and d) **Br-MOP**. Note that for **MOP**, the −78 °C data was collected after the cage was activated at 140 °C instead of 120 °C.

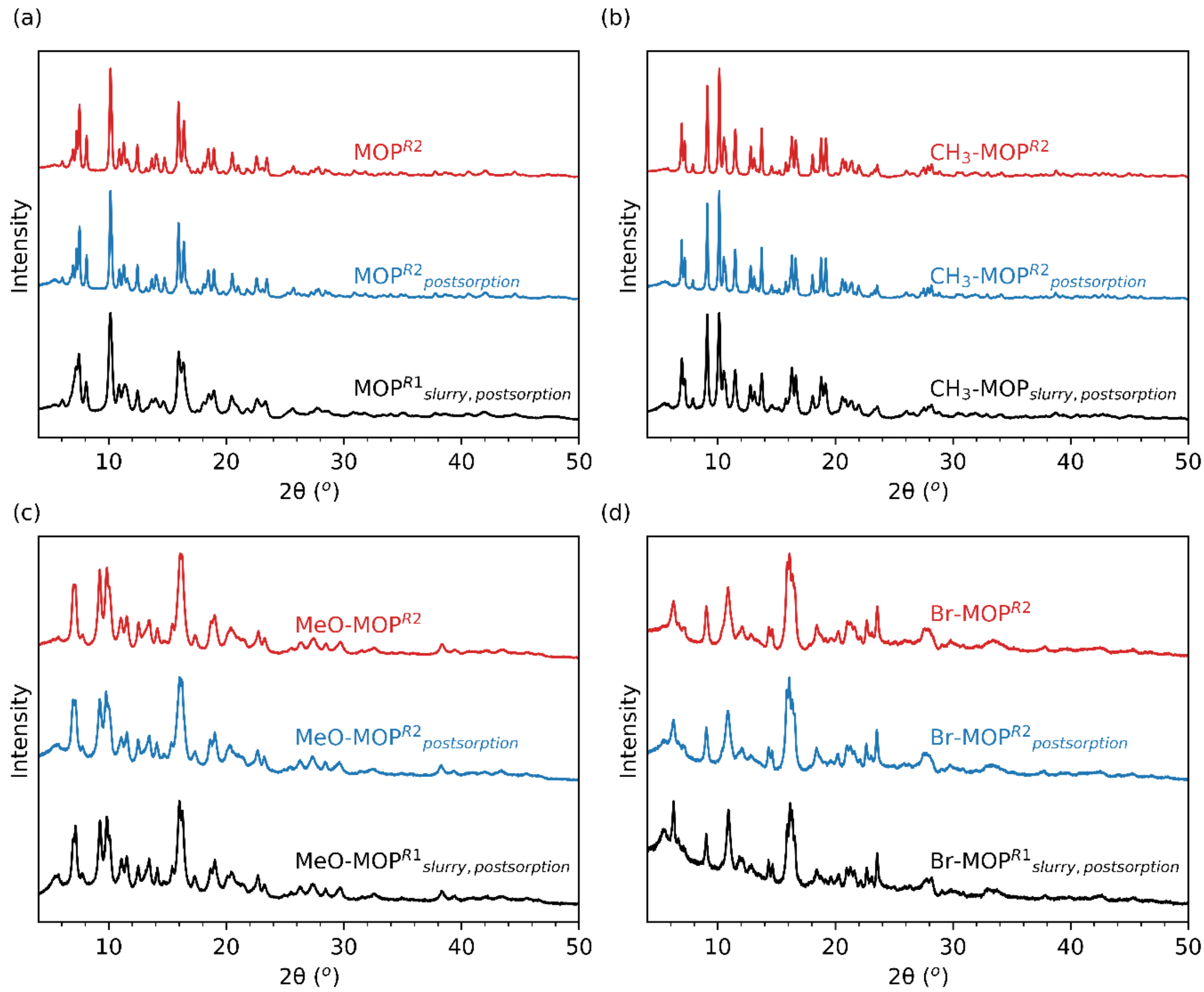

Fig. S39. Post-sorption PXRD diffractograms for a) **MOP**, b) **CH_3-MOP**, c) **MeO-MOP**, and d) **Br-MOP** samples for comparison showing the structure of the MOP is retained after activation and gas sorption.

References

1. B. Doñagueda Suso, Z. Wang, A. R. Kennedy, A. J. Fletcher, S. Furukawa, G. A. Craig, *Chem. Sci.*, 2024, **15**, 2857–2866.

2. B. Doñagueda Suso, A. Legrand, C. Weetman, A. R. Kennedy, A. J. Fletcher, S. Furukawa, G. A. Craig, *Chem. Eur. J.*, 2023, **29**, e202300732.

3. A. A. Coelho, *J. Appl. Cryst.*, 2018, **51**, 210.

4. CrysAlisPRO, Rigaku Oxford Diffraction, 2025.

5. G. Sheldrick, *Acta Crystallogr.*, 2015, **A71**, 3.

6. L. J. Bourhis, O. V. Dolomanov, R. J. Gildea, J. A. K. Howard, H. Puschmann, *Acta Crystallogr.*, 2015, **A71**, 59.

7. O. V. Dolomanov, L. J. Bourhis, R. J. Gildea, J. A. K. Howard, H. Puschmann, *J. Appl. Cryst.*, 2009, **42**, 339.

8. A. Saha, S. S. Nia, J. A. Rodríguez, *Chem. Rev.*, 2022, **122**, 13883.
